# The Insight-Inference Loop: Efficient Text Classification via Natural Language Inference and Threshold-Tuning

**DOI:** 10.1177/00491241251326819

**Published:** 2025-04-18

**Authors:** Sandrine Chausson, Marion Fourcade, David J. Harding, Björn Ross, Grégory Renard

**Affiliations:** 1 School of Informatics, 151022 The University of Edinburgh, Edinburgh, Midlothian, UK; 2 Department of Sociology, 1438 University of California Berkeley, Berkeley, CA, USA; 3 Independent Researcher

**Keywords:** text analysis, natural language processing, computational methods, active learning, few-shot learning, large language models

## Abstract

Modern computational text classification methods have brought social scientists tantalizingly close to the goal of unlocking vast insights buried in text data—from centuries of historical documents to streams of social media posts. Yet three barriers still stand in the way: the tedious labor of manual text annotation, the technical complexity that keeps these tools out of reach for many researchers, and, perhaps most critically, the challenge of bridging the gap between sophisticated algorithms and the deep theoretical understanding social scientists have already developed about human interactions, social structures, and institutions. To counter these limitations, we propose an approach to large-scale text analysis that requires substantially less human-labeled data, and no machine learning expertise, and efficiently integrates the social scientist into critical steps in the workflow. This approach, which allows the detection of statements in text, relies on large language models pre-trained for natural language inference, and a “few-shot” threshold-tuning algorithm rooted in active learning principles. We describe and showcase our approach by analyzing tweets collected during the 2020 U.S. presidential election campaign, and benchmark it against various computational approaches across three datasets.

## Introduction

The advent of increased computing resources, machine learning (ML) algorithms, and more easily accessible and large-scale text data over the last decade allows social scientists to use “text as data” to understand a wide range of social phenomena (Evans and Aceves 2016; Grimmer, Roberts, and Stewart 2022; Salganik 2019). Yet these new opportunities are not without challenges and risks. From a practical perspective, extracting data from large quantities of text still calls for considerable investment in the human labeling of text. Supervised machine learning algorithms require labeled training data on which to learn before they can be deployed at scale on an entire corpus. Well-trained human labelers are essential to this process (Grimmer et al. 2022), and when supervised ML algorithms are trained on enough data produced by such labelers, they can perform as well as them, particularly when the coding task is relatively straightforward (Do, Ollion, and Shen 2022). More importantly, many social scientists are rightly suspicious about the wisdom of turning over the coding of data to a computer, particularly given the “black box” nature of many ML algorithms.^1^ These challenges are amplified when labeling tasks go beyond the straightforward identification of topics and involve detecting more abstract concepts or the valence of an argument for or against a particular idea. For such tasks, it would seem that much greater involvement by the human social scientist is necessary to ensure accurate and reliable results. In other words, the first challenge is to develop methodologies that efficiently “augment”—rather than replace—the human collection and labeling of textual data, as Do, Ollion, and Shen (2022) put it.

This imperative—to augment rather than replace—is true on an analytical level as well. The social world is not an unknown tabula rasa, and should not be treated that way by computational social scientists. So a related challenge is to develop an approach that speaks to existing analytical practices and makes use of the wide array of theoretical insights that social scientists have already developed about human interactions, social structure, and institutions (Bonikowski and Nelson 2022). New computational approaches also allow for the deployment of a more inductive, exploratory epistemological stance that contrasts with the deductive logic embedded in traditional statistical methods. This has led a number of scholars to suggest that epistemologically, computational tools are closer cousins to qualitative approaches such as ethnography or archival research than to quantitative approaches that revolve around deductive reasoning and the testing of hypotheses. Sociologists, for instance, have extolled the usefulness of using computational tools in combination with qualitative approaches founded on grounded theory (Nelson 2020), abductive reasoning (Brandt and Timmermans 2021; Timmermans and Tavory 2022), and the extended case method (Pardo-Guerra and Pahwa 2022). Outside of sociology, scholars have probed the integration of natural language processing (NLP) tools and other ML techniques with history and structural anthropology (Guldi 2023; Pedersen 2023).

In this article, we present and illustrate an approach to large-scale text analysis based on natural language inference (NLI) that moves us toward overcoming these two challenges by (a) reducing the amount of human-labeled data that is necessary to train an ML model, and (b) efficiently integrating the social scientist into multiple critical steps in the workflow while providing multiple opportunities for them to check and adjust the work of the computer through qualitative interventions. To be specific, this method allows us to code a corpus with predefined statements, such as “Joe Biden is sold out to China,” “The economy is doing well,” or “We need a strong and stable government.” These statements, which we call “claims,” are text classification tools that researchers can flexibly design to capture different types of information, depending on their interest. For instance, the above claims can help us identify a political stance (“anti-Biden”), measure a sentiment (positive feelings about the economy), or categorize topics in a text (“this sentence is about politics”).

This claim-based approach gives researchers significant control over their analysis while allowing them to take advantage of recent advances in NLI and ML technologies. Specifically, in this article, we combine claims generation with transformer-based large language models and a “few-shot” threshold-tuning algorithm rooted in active learning principles. We show that there are several advantages to this process. First, our approach requires less training data, while avoiding the need to “fine-tune” the large language model’s (LLM's) parameters (in the machine learning sense of the term). This makes it computationally less expensive and technically more accessible than existing computational solutions to text classification. Second, the method's efficiency in terms of both training data and computation allows for an iterative workflow between human decision-making and ML. This “insight-inference loop,” as we call it, provides greater opportunities for human intervention in the classification process and more subtle and conceptual human inputs than traditional methods. It can accommodate both deductive and inductive approaches, or a combination of the two—for example, when some concepts are pre-specified based on theory and substantive knowledge and others emerge from the data itself. Finally, our approach provides an end-to-end solution for generating the limited amount of training data it requires from a completely unlabeled corpus, making it particularly useful for applications where a training dataset does not already exist.

We begin by providing some background on LLMs and their application for social science research, with a focus on the text analysis algorithms underlying our methodology. We then provide a step-by-step walkthrough of our approach, using real data to illustrate each step. This illustration focuses on mentions of China on Twitter in relation to Joe Biden and Donald Trump, respectively, in the context of the 2020 U.S. presidential election cycle. Next, we evaluate our approach using three test sets: “one from our 2020 U.S. elections Twitter corpus, and two benchmark datasets from the text analysis literature”: a sentiment classification dataset from Barberá et al. (2021) and a topic classification dataset from Burst et al. (2020) (both are referenced by Laurer et al. (2024)). In this evaluation, we validate our approach by comparing it to human annotations. We also assess its performance against other computational methods, considering both how well they perform and how much data and computation they each require. Finally, we provide guidance on when our approach is most likely to be suitable, discuss the scope of use cases it can cover, and reflect on its significance in the context of sociological research.

## Supervised Learning, Transfer Learning, and LLMs

Recent decades have seen a series of advances in NLP and ML that have been adopted by social scientists to study large text datasets. The perhaps most well-established alternative to manually coding the data relies on lexicons such as Linguistic Enquiry and Word Count (LIWC), originally developed in the 1990s. Lexicons contain lists of words associated with a class of interest (e.g., positive emotions) used to compute scores for input text. An example of such an approach is Golder and Macy's (2011) analysis of seasonal mood variation on Twitter.

Lexicon-based measures being rather crude, modern NLP has moved on to ML-based approaches, notably “supervised learning” (for a review for social scientists, see Molina and Garip 2019). The latter relies on the availability of data where class membership is known. From this training data, an algorithm learns model parameters or “weights.” The resulting classification model can then be used to classify new, unseen examples. For example, Yu, Kaufmann, and Diermeier (2008) trained party classifiers on U.S. Congress speeches this way. When the researcher does not have access to training data, a dataset needs to be “labeled” or “annotated.” In this procedure, not unlike coding in qualitative data analysis, one determines for each data point (e.g., a sentence, paragraph, and article) whether or not the class of interest applies. Earlier supervised learning approaches were time-intensive as they would require a lot of labeled training data to perform well.

Newer supervised learning approaches perform better and require less labeled training data by first “pre-training” a model to represent language probabilistically using a large corpus of unlabeled data, and then leveraging this model to train a classifier on a smaller dataset of labeled data. Such approaches make use of a property called “transfer learning,” as the pre-trained model's capabilities are retained by the downstream classifier. In the first generation of such approaches (Mikolov et al. 2013), the pre-trained model would be a small neural network used to generate “static word embeddings,” that is, re-usable numerical vector representations of words. These embeddings would then serve as the input into the final classifier.^2^ For an example in sociology, see Zhang and Pan (2019).

Roughly since the release of bidirectional encoder representations from transformers (BERT) (Devlin et al. 2019), the dominant paradigm for text classification has shifted away from static word embeddings to pre-training increasingly large neural networks capable of “next word” generation, also called large language models (LLMs). The final classifier is then obtained by “fine-tuning” a subset of the LLM's “weights” on the small dataset labeled for the task at hand. The benefits of this “pre-train/fine-tune” paradigm have been considerable, with fine-tuned LLMs often outperforming older supervised learning approaches on textual classification tasks. Moreover, pre-trained language models are widely available, meaning that researchers interested in training a new classifier only need to do the fine-tuning. This has greatly contributed to making NLP methods more accessible to research beyond computer science (e.g., Coan et al. 2021; Do, Ollion, and Shen 2022; Röchert et al. 2022; Wankmüller 2019).

While a significant improvement in training a classifier from scratch, the cost of fine-tuning a pre-trained LLM remains non-negligible. Fine-tuning datasets still typically contain several thousand annotated examples. Creating datasets of this size remains expensive and time-consuming, with the best results achieved with substantial investments in training the annotators. This can leave little room for experimentation: once created, the classification schema is hard to modify without completely re-annotating the dataset. Moreover, fine-tuning a pre-trained LLM remains somewhat computationally expensive and technical, particularly when optimizing the process by experimenting with different fine-tuning “hyper-parameters” (e.g., number of epochs, learning rate, choice of optimizer, warm-up ratio, and weight decay).

To overcome some of these limitations, research has emerged on strategies to minimize the need for annotation. For instance, strategies that fall under the umbrella of active learning typically entail dynamically selecting the next example to annotate from a pool of unannotated data based on its “informativeness” or novelty relative to existing annotations, or its estimated impact on the model's performance (Ren et al. 2021). Alternatively, in an attempt to obtain general-purpose classifiers, researchers have tried using pre-trained language models fine-tuned for a *different* but more general task, such as the task of recognizing textual entailment, or what is termed “Natural Language Inference” (e.g., Yin, Hay, and Roth 2019). As we describe further below, natural language inference is an approach to a classification task that involves inferring whether a document entails a “hypothesis.” Applying such a model to a new task without any further fine-tuning is called “zero-shot” text classification. Related to but different from this is the recent discovery that, as language models get increasingly large (in the number of weights) and are pre-trained on increasingly more unlabeled data, they require fewer and fewer labeled examples to perform well on a downstream task (Brown et al. 2020). Giving a language model only a few labeled examples to learn from is known as “few-shot” learning. Although increasingly common in the computer science literature, social scientists have rarely used pre-trained NLI classifiers for few-shot learning (Burnham 2024). One example is Burnham (2024), who uses this approach for stance detection regarding COVID-19 mitigation policies like masking and vaccines in Twitter data.

Somewhat more common among social scientists is to prompt generative LLMs for textual classification tasks (e.g., Gilardi et al. 2023; Törnberg 2023, 2024). A number of very LLMs have been created, including GPT-4 (OpenAI), Llama (Meta), PaLM (Google), Claude (Anthropic), and BLOOM (BigScience). One disadvantage of relying on such models, however, is that only a handful of organizations have the resources (in terms of both data and computation) to create them, at great financial and environmental costs (Bender et al. 2021). Some of these models are not published and are only used internally. Others are only accessible to researchers through an application programming interface (API), which means that fine-tuning the model or using it to classify data requires transmitting one's data to the company providing the model, which leads to legal and ethical concerns. Only some of these models have been released to the public, such that researchers can download the code used to train them and/or their weights, fine-tune them, and use them freely for classification and other purposes. In this article, we only make use of models whose weights are openly available and briefly touch upon the subject of closed models in the “Discussion” section.

Generally speaking, supervised learning has been most closely associated with confirmatory, deductive approaches to research (Evans and Aceves 2016), as the researcher would put a great deal of effort into deciding on a suitable set of categories and creating training data for these categories. Inductive, theory-building research approaches have been most closely associated with unsupervised ML techniques, which work without labeled examples or categories, such as topic modeling (Baumer et al. 2017; Nelson 2020). The recent developments in NLP and ML have changed this picture. Less and less training data is necessary to perform classification. As we show in this article, this makes it possible for social scientists following an inductive approach to create text classifiers ad hoc and iteratively refine them as their understanding of the data improves.

## Our Approach and its Application, Step-by-Step

**Figure 1.**
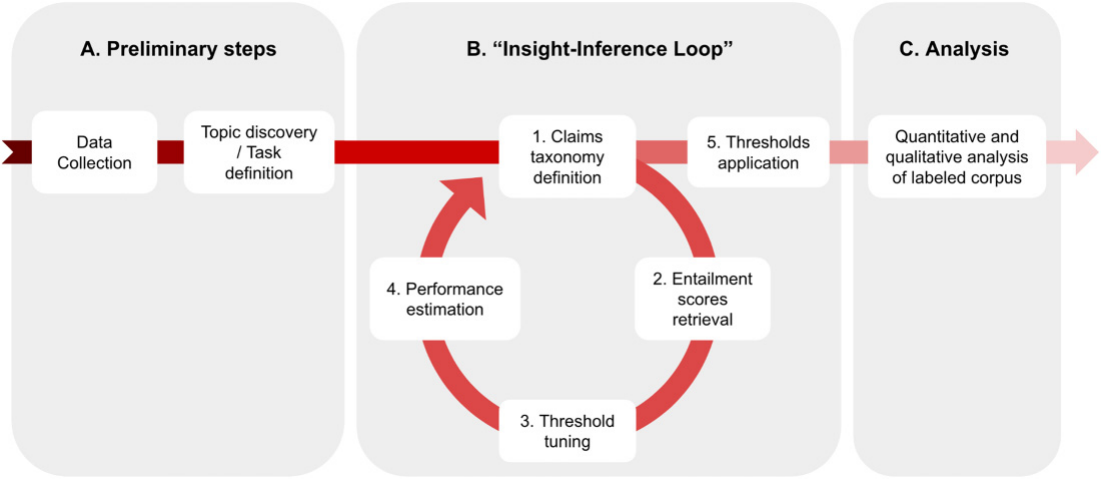
The “Insight-Inference Loop” framework. This framework is itself only a sub-part of the end-to-end computational social science research process. It is namely independent from the preliminary data collection and task definition steps, and from the analysis of results that follows. Steps 1–4 in the framework form an iterative loop, meaning that the researcher may perform these steps multiple times until satisfied with the results they yield.

We propose an approach to text classification that can be broken down into five steps. In the first step, we define a taxonomy of claims that we are interested in detecting in the corpus. Next, we use an off-the-shelf LLM fine-tuned for the task of NLI to obtain an “entailment” score for every pair of claims in the taxonomy and document (e.g., sentence, paragraph, and tweet) from the corpus (Step 2). Once these scores are obtained, we select a very small sample of documents, typically between 10 and 40 per claim, using the Probabilistic Bisection algorithm and annotating them to find an optimal threshold for each claim (Step 3). We then estimate the performance of the system for the chosen taxonomy using three simple heuristics (Step 4). Depending on how well the system performs, we can choose to modify the taxonomy and essentially repeat the previous four steps: this constitutes an iterative loop in the methodology. Eventually, we apply the thresholds found in Step 3 to obtain claim labels for all documents in the corpus. Figure 1 describes this logic of inquiry.

In what follows, we describe each of these steps in further detail and illustrate them using data previously obtained from Twitter. In November 2019, we built a large dragnet to collect tweets that were concerned, one way or another, with the then upcoming 2020 U.S. presidential election, at the time one of the most critical political events in a generation or more. We did not know what we would ultimately use the data for, however, two considerations guided us. First was the recognition of the historical—indeed potentially transformative—significance of this widely anticipated event. Second was the understanding that tweets had to be collected as they were being released in real-time, before various corrections had taken place (by users or by the platform itself).

The dataset was collected by querying the Twitter API using the following keywords: “Donald Trump,” “Bernie Sanders,” “Joe Biden,” “US election,” “Democratic candidate,” “Republican candidate,” “2020 United States presidential election,” and “presidential re-election” (see Appendix A, supplemental materials, for more information on the Twitter API and the data collection process). The names of several other Democratic candidates (e.g., “Elizabeth Warren” and “Kamala Harris”) were also included in the initial query since Joe Biden (who would ultimately win this election against Donald Trump) did not become the presumptive Democratic nominee until April 8, 2020—the date when Bernie Sanders exited the race for the presidency. For this analysis, however, we excluded data collected with these keywords to concentrate on the dynamics between Donald Trump and the two main Democratic candidates, Joe Biden and Bernie Sanders (who had a large and devoted Twitter following), as they unfolded over many months before and after the election. This strategy also allows us to reduce the volume of data to be more manageable while retaining Tweets expected to be the most relevant given the final outcome of the election. This filtering was done using a simple dictionary, or “token matching,” method.

The data collection process ran continuously from November 8, 2019, to October 24, 2022. We thus ended up with a very rich dataset of around 2.4 billion tweets,^3^ or about 5TB of data, at our disposal that we could explore in multiple ways.

### Defining a Taxonomy of Claims

To explore a rich and complex dataset like this one, a researcher must first decide what to look for. Their very first task, then, is to develop a set of “claims” that capture a statement or idea to identify in unseen text. What, however, is a claim? We define it as a declarative statement, simple enough to be expressed as a sentence with a single clause. Such a statement may be present or absent in any particular segment of text, and it must be possible to judge from the text itself whether or not it is present. The statement can be true (“The sky is blue”) or false (“The Earth is flat”), verifiable (“Edinburgh is in Scotland”) or unverifiable (“We live in a multiverse”), objective (“Interest rates are decreasing”) or subjective (“Biden is great”), affirmative or negated (“Fairies don't exist”). It can describe the world in some capacity or can also simply convey a position or perspective (“I support the Boston Celtics,” “I believe in magic,” and “I feel sad”). It cannot be a command or question. A claim can be used to identify a more abstract conceptual category or “class” such as a sentiment (e.g., “I feel sad” can stand for the class “negative”), topic (e.g., “Interest rates are decreasing” can stand for the class “economy”) or stance (e.g., “Biden is great” can stand for the class “pro-Biden”).

One can approach the claims development task inductively or deductively depending on the nature of the research question and analysis. Proceeding deductively would mean that the researcher writes the claims based on domain knowledge or theory, producing a set that is thought to be complete enough to identify all relevant concepts. For an example of the latter, consider Chausson and Ross (2024), where a taxonomy with claims such as “I dislike myself” or “I feel sad much of the time” was created from the BDI-II, a resource widely used by mental health professionals for the diagnosis of depression, in order to detect symptoms of depression in social media posts.

To proceed inductively, on the other hand, one would generate the set of claims as a result of an exploration of the corpus itself. This stage of the process would rely on conventional sociological approaches to inductive analysis of linguistic data (whether text or speech), in which the researcher relies on a close examination of that data and on substantive domain knowledge to identify important themes, social variations, or conversational codes (for good empirical illustrations, see Anspach 1988; Labov 2006; Wagner-Pacifici 1994). For instance, one way to carry out this exploration is by sampling a moderate number of documents at random from the corpus, being careful to stratify the sample by key dimensions such as time, source, or other document meta-data. Stratifying here ensures that all essential categories are represented in the data even if they appear at different rates, which is particularly important because the sample here is relatively small to allow for efficient review. The size of the sample will depend on how much variation the researcher expects to see, as corpora with more variation will require a larger sample of documents to capture all important variations in the sample. It is also possible to use inductive topic-modeling techniques such as LDA (Blei et al. 2003) or BERTopic (Grootendorst 2022) to assist the researcher in the process of exploring the data. Given the inherent subjectivity of this inductive process, social scientists have a range of strategies for improving and measuring reliability, most of which involve multiple independent coders and measuring agreement between them (see Grimmer et al. 2022 for applications in text analysis). Of course, one could also proceed both inductively and deductively in this stage by writing some claims based on a priori knowledge and developing others inductively.

In the case of our 2020 U.S. presidential elections data, we decided to adopt an inductive approach. A first manual exploration revealed that our data touched on a number of different topics. It contained debates about masks and school closures in the context of the COVID-19 pandemic; documented the crystallization of a wave of social protests (Black Lives Matter) like no other in recent memory; chronicled the lead-up to, experience, and aftermath of an attempted coup in the United States. The possibilities were endless. In the end, we chose to focus on representations of China as a case study on which to demonstrate our method.

The reasons for choosing to focus on China were both substantive and practical. Substantively, China emerged early on in our deliberations as a particularly interesting subject to zero in on. As a rising world power, led by an increasingly authoritarian leader since 2012, the country has been the object of rising economic and geopolitical anxiety in the United States, which sharply intensified with the onset of the COVID-19 pandemic. A Pew Research Center (2023) survey shows that negative opinions of the country have been rising fast since 2018. Gallup polls reveal that since 2021, Americans have viewed China as the United States’ greatest enemy (Younis 2023). Unsurprisingly, these feelings are reflected (or exhorted?) online. In a study of Reddit and 4Chan, Shen et al. (2022) found that online interest in China increased notably during the pandemic period, as did the use of anti-Chinese slurs. Anti-Asian hate crimes have spiked (FBI 2023).

On a practical level, the topic of China is relatively easy to parse out from the mass of unrelated tweets: we only needed a reference to “China” or “Chinese” to create a subsample. We extended this selection to include all tweets containing the terms “Kung Flu” (first used by Donald Trump at a rally in Tulsa, Oklahoma on June 20, 2020) and “Wuhan” (with the casing being irrelevant), given the prevalence of these terms in online discussions of COVID-19.

Our next task was to define a list of claims pertaining to the way China was being talked about in our U.S. election sample. Two of the authors independently read a random sample of 200 tweets per month from the dataset (for a total of 3,200 tweets, given that we carried out this review in February 2021), inductively identified prominent and interesting claims, and then conferred on a final list for the taxonomy. This process took about 20 hr. The full list of claims we identified is given in Appendix B, supplemental materials. Interestingly, that inspection of the data did not find many claims relating to trade, despite the importance of this topic in prior literature (e.g., Aldrich, Lu, and Kang 2015; Autor et al. 2020; Che et al. 2022; Jin, Dorius, and Xie 2022; Xie and Jin 2022). One especially common trope, however, of which there were several variations, was the notion of an unethical relationship between Joe Biden (or members of the Biden family) and Chinese authorities or businesses. Similar claims were occasionally made about Donald Trump and members of his family, but they were much more rare. A first iteration using our claims detection methodology over a sample of the data confirmed these observations (more about this below).

Given these initial insights and the polarized nature of this particular electoral contest, our final taxonomy focused on a symmetrical analysis of Joe Biden's and Donald Trump's perceived relationship to China. Formally, this choice of claims aimed to answer the following research question: in the context of an intensely polarized electoral campaign, how are mentions of a special relationship with China distributed between the two presidential candidates and over time? Concretely, addressing this question meant selecting two parallel classes of six claims each, the first about Joe Biden and the second about Donald Trump, from the list of claims obtained from our preliminary review of the data. The resulting taxonomy is presented in Table 1. Note that our use of two parallel sets of claims in our taxonomy is motivated only by our choice of research questions and is not a necessary feature of our methodology. For an example of an “asymmetrical” taxonomy, please see an application of our method for topic classification in the “Evaluation” section further in this.

**Table 1.** Taxonomy of Claims for Our Analysis of the 2020 U.S. Presidential Elections Data.

**Class 1**: China and Joe Biden	**Class 2**: China and Donald Trump
1_1: Biden is sold out to China 1_2: Biden does business in China 1_3: China blackmails Biden 1_4: Biden works for China 1_5: The CCP controls Biden 1_6: Biden's policies hurt the United States	2_1: Trump is sold out to China 2_2: Trump does business in China 2_3: China blackmails Trump 2_4: Trump works for China 2_5: The CCP controls Trump 2_6: Trump's policies hurt the United States

### Obtaining Entailment Scores Using an NLI Model

As mentioned earlier, researchers have proposed to use pre-trained models fine-tuned for the general task of “Natural Language Inference” (NLI), also called “Recognizing Textual Entailment,” as general-purpose “zero-shot” classifiers. NLI in its simplest form is a binary classification task where, for a given “extract” and “hypothesis,” the aim is to classify whether or not the extract entails the claim (Zeng et al. 2021). The idea then is to re-frame the classification task of interest to be equivalent to an NLI task. For instance, Yin, Hay, and Roth (2019) performed topic classification by using hypotheses such as “*This text is about politics*,” “*This text is about sports*,” or “*This text is about health*” as input into the NLI model alongside each extract to be classified. The hypothesis for which the NLI model returns the highest entailment score then corresponds to the topic of the extract.

The motivations behind using NLI models for classification tasks are several. First, many textual classification tasks can relatively easily be rephrased into NLI tasks. Secondly, compared to the original pre-trained model, a version fine-tuned for NLI should be better at detecting logical and semantic inferences, both of which are essential to most text classification tasks. And finally, “entailment” being a structural and semantic property of language in general rather than one that pertains to a particular type of discourse, NLI can be thought of as more “upstream” compared to most textual classification tasks. This makes NLI models suitable for textual classification for very different domains and corpora.

In order to detect the claims developed in the previous step in our corpus, we leverage the capabilities of an NLI model called BART_MNLI_. This is a version of the BART pre-trained LLM (Lewis et al. 2019), which was fine-tuned on the multi-genre NLI (MNLI) dataset (Williams, Nangia, and Bowman 2017) and is available off-the-shelf via the HuggingFace platform. Having already been fine-tuned on the MNLI dataset, the BART_MNLI_ model can then simply be deployed *without any further training* on any corpus, such that every combination of document from the corpus and claim from the taxonomy of claims defined in the previous step is provided as input. For each such combination, the model returns an entailment score, which corresponds to the model's confidence that the extract makes (or entails) the claim. Given the limited amount of text the NLI model is able to handle as input, documents ought to be relatively short, that is, sentences or paragraphs. Splitting the text into sentences using libraries such as spaCy (Honnibal et al. 2020) or NLTK (Bird, Klein, and Loper 2009) might, therefore, be a necessary pre-processing step.

It is worth noting that the use of the BART_MNLI_ model specifically is not a requirement of the approach we propose. Rather, any model designed to return an entailment score would be appropriate, irrelevant of its specific architecture or the NLI dataset it is fine-tuned on. It is also possible that models fine-tuned for different but related semantic tasks, such as paraphrasing or summarization detection, could be appropriate, however, we have not experimented with these.

In the case of our own corpus, we defined each tweet to be a document. To reduce the amount of computation needed, we did not run the NLI model on retweets but only on original tweets, replies, and citations. We also filtered the data to keep only English tweets using the “langdetect” Python package (Dalnik 2021). Using a single RTX 2070 SUPER GPU, it took ∼28 hr overall to obtain the NLI scores for the 1,310,540 tweets that remained in our dataset and 12 claims in our list, or about 0.0064 s per tweet/claim pair (see Appendix C, supplemental materials, for the scores distributions for each of our claims).

### Finding Thresholds by Annotating a Small Sample of Data

The entailment scores returned by the NLI model cover the full 0 to 1 range. What we need, however, is a binary label for each document/claim pair. Our system therefore requires, for each claim, a threshold somewhere between 0 and 1. While we could use the intuitive threshold of 0.5, we find that in practice this does not work well. Indeed, the NLI model seems to exhibit bias, being overconfident in its prediction for some claims and underconfident for others. This can be explained by the domain shift that occurs when applying an NLI model to data that significantly differs from the one it was originally trained on. As a result, a threshold of 0.5 can lead to very different performance depending on the claim. Moreover, unless the prevalence of a claim in the corpus can be established a priori, selecting the *n*% documents with the highest score is not an appropriate solution either. Finally, given that the presence of a claim in a text can be relatively ambiguous (particularly for more abstract claims), we believe it is important for our system to account for the analyst's subjective definition of the claim. To meet these criteria, we propose to use an algorithm rooted in the statistical heuristic of probabilistic bisection (Burnashev and Zigangirov 1974; Horstein 1963) to find these thresholds with as few annotations as possible. In what follows, we provide a high-level description of this algorithm. For a more in-depth description of this process, please refer to Appendix D, supplemental materials, and Chausson and Ross (2024).

**Figure 2.**
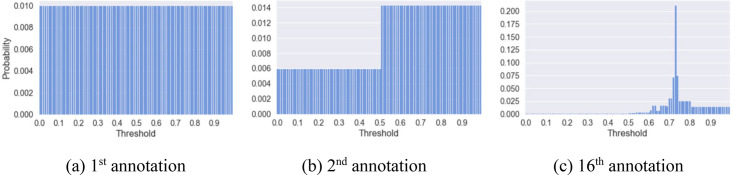
Probability distribution over threshold location at different stages of the threshold tuning step.

First, we start by assuming that the location of the threshold is represented by a uniform probability distribution spanning the entire 0 to 1 range (see Figure 2a). We then select the median point of this distribution, which at the initial timestep is simply equal to 0.5, and sample a document with an entailment score equal to this median. If the researcher annotates this document as **not** implying the claim, we can infer that the optimal threshold we are looking for will probably be **higher** than this median. We therefore update the probability distribution so that the probability of the threshold being less than that median is scaled **down**, and the probability of it being greater than that median is scaled **up** (see Figure 2b). If, on the other hand, the user annotates this document as indeed implying the claim, we can infer that the optimal threshold we are looking for will probably be **lower** than the current median. We therefore update the probability distribution accordingly, scaling **up** for values below the median and **down** for values above. Steps 2 and 3 are repeated until the probability distribution is considered sufficiently concentrated around a threshold: for example, when 95 percent of the probability mass around the median is concentrated in a range equal to or smaller than 0.10 (see Figure 2c).^4^ The median of the final probability distribution then becomes the threshold for the claim.

Our reliance on a small sample of annotated data for threshold-tuning means that we do not use NLI models in a “zero-shot” way as suggested by Yin, Hay, and Roth (2019). Instead, our approach can be described as “few-shot.” Moreover, dynamically updating the probability distribution after each annotation and using this information to select the next data point to annotate very much roots this threshold-tuning approach in an active learning framework. Importantly, this step does not require updating the parameters of the NLI model itself and is therefore very different from the process of “fine-tuning” an LLM, a computationally and technically more taxing process. Finally, this “threshold-tuning” step is essentially how the classifier is made to align with the annotator's interpretation of the claim (ideally the researcher themselves), and it only needs to be performed once for each claim. In other words, the methodology does not require training external annotators, thus allowing for a faster exploration of the data. As we will show in our evaluation later, the threshold-tuning step is essential to improve the quality of the classifications returned by the system.

**Figure 3.**
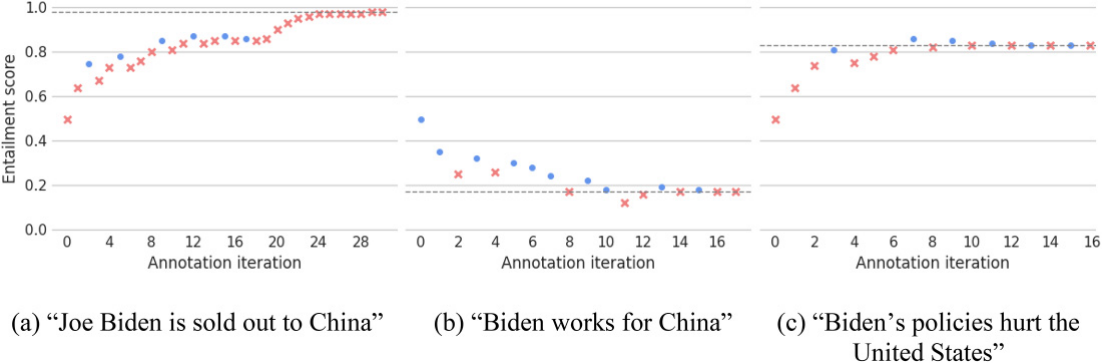
Annotation traces for three of our claims. Dots represent positive annotations (i.e., when the annotator deemed that the tweet contained the claim), while crosses represent negative annotations. The dashed line represents the final thresholds.

We use this threshold-tuning algorithm for each claim from our own taxonomy. Figure 3 shows the annotation traces for three of our claims (see Appendix E, supplemental materials, for all claims). As we can see, the algorithm first converges quickly towards the final threshold but progressively slows down. Moreover, the probabilistic nature of the algorithm ensures that earlier annotations can be overridden by later ones, thus allowing for noise in the entailment scores. We find that in practice a researcher could identify the optimal threshold for a claim using this algorithm in about 20 min per claim (21.3 ± 8.4 annotations per claim on average for our case study).

### Estimating the Performance of the System for Each Claim

Even after having tuned the thresholds, our claims detection methodology might perform better for some claims than others. This can be due to the model's own biases, the formulation of the claim, or the content of the corpus itself. One way to determine whether this is the case in a traditional ML context would be to ask human annotators to annotate a small sample of the data. The annotations in this “validation set” can then be treated as the ground truth, and compared to the labels output by the system to measure its performance. Claims that perform poorly on the validation would then be rephrased, replaced, or simply excluded. While such a validation set is typically much smaller than the training set used to train or fine-tune the classifier, the costs of re-annotating the validation set for each new claim can quickly add up.

In the case of our own claims detection methodology, however, information gathered during the threshold-tuning step already provides some insights into whether or not a particular claim seems to be detected properly by the system. In what follows, we present three heuristics that can be used to validate the quality of the classification. These heuristics can be used as either an alternative or in addition to a conventional validation set.

#### **Heuristic 1:** No true positives

Data points the researcher annotated as including the claim during the threshold-tuning step can be treated as *true positives* if their score is greater than the final threshold, and *false negatives* otherwise. Similarly, data points annotated by the researcher as *not* including the claim can be treated as *false positives* if their score is greater than the final threshold, and *true negatives* otherwise. With this in mind, the first heuristic we use is to eliminate any claims for which no true positive was found. An example of such a scenario can be seen in Figure 3a for claim 1_1 (“Joe Biden is sold out to China”). Column 2 of Table 2 summarizes the number of true positives for all the claims and highlights those which would need to be eliminated according to this first heuristic. These include 1_1 (“Joe Biden is sold out to China”), 1_2 (“Biden does business in China”), 1_3 (“China blackmails Biden”), 2_3 (“China blackmails Trump”), and 2_6 (“Trump's policies hurt the United States”).

**Table 2.** Summary of Performance Estimation Heuristics for All 12 Claims Derived From the Threshold-Tuning Annotations.

Claim	Number of true positives	Weighted accuracy	Proportion of data in 95% CI	Overall
1_1: Biden is sold out to China	0	0.68	0.125	Screen out
1_2: Biden does business in China	0	0.84	0.099	Screen out
1_3: China blackmails Biden	0	0.98	0.036	Screen out
1_4: Biden works for China	8	0.88	0.182	Pay attention to confidence intervals on visualizations
1_5: The CCP controls Biden	5	1.00	0.312	Pay attention to confidence intervals on visualizations
1_6: Biden's policies hurt the United States	2	0.95	0.241	Pay attention to confidence intervals on visualizations
2_1: Trump is sold out to China	2	1.00	0.014	Keep
2_2: Trump does business in China	1	0.89	0.029	Keep
2_3: China blackmails Trump	0	0.95	0.004	Screen out
2_4: Trump works for China	10	0.82	0.002	Might benefit from further testing
2_5: The CCP controls Trump	2	0.81	0.001	Might benefit from further testing
2_6: Trump's policies hurt the United States	0	0.73	0.020	Screen out

#### **Heuristic 2**: Accuracy weighted by distance to the threshold

The proportion of true positives and negatives in the threshold-tuning annotations is sufficient to calculate an accuracy score. The picture painted by this single metric might, however, be distorted by the fact that this sample is not randomly selected from the data. Rather, it predominantly contains data points with entailment scores concentrated around the final threshold. However, we expect entailment scores to be somewhat noisy, with misclassifications being more likely to occur close to the final threshold. Misclassifications occurring close to the threshold are therefore not as problematic as those occurring significantly above or below.

Consequently, as our second heuristic, we propose to use a version of the accuracy score where each data point is weighted by its distance to the threshold. That is, we take the distance of each data point from the threshold (i.e., the absolute value of the difference between the data point's entailment score and the claim's threshold). If the data point is a false negative or false positive, we negate this value: we call this the “polarized distance.” We then sum all of our polarized distances and divide this by the sum of all (non-polarized) distances. Finally, we normalize this score to range between 0 and 1. Formally, this is represented by the following equation:

$$W\;\mathrm{Acc}(c)=\left(\frac{\sum_{d\in D_{c}}\{\begin{array}{cc} |s_{d,c}-t_{c}|, & \mathrm{if}\;y_{d,c}=z_{d,c}\\ -|s_{d,c}-t_{c}|, & \mathrm{otherwise} \end{array}}{\sum_{d\in D_{c}}|s_{d,c}-t_{c}|}+1\right)/2,$$

where $D_{c}$ is the sample of data annotated during the threshold-tuning of claim *c*; *d* is a data point from that sample; $s_{d,c}$ is its entailment score; $t_{c}$ is the final threshold obtained for claim *c*; $y_{d,c}$ is the prediction (i.e., true if $s_{d,c}\;\geq t_{c}$ and false otherwise); $z_{d,c}$ is the annotation (i.e., true if the researcher indicated that the data point contains the claim and false otherwise) and 1 is added to the fraction and the result is divided by 2 to normalize the value into the [0, 1] range.

As shown in the third column of Table 2, claims 1_1 (“Joe Biden is sold out to China”) and 2_6 (“Trump's policies hurt the United States”) both have a “weighted accuracy” under 0.75. We therefore rule out these claims. Moreover, claims 1_2 (“Biden does business in China”), 2_4 (“Trump works for China”), and 2_5 (“The CCP controls Trump”) score below 0.85, so we flag these as potentially problematic.

#### **Heuristic 3**: Confidence interval and scores distribution

The threshold-tuning step, once completed, returns a probability distribution for the estimated location of the threshold given the annotations selected with the probabilistic bisection algorithm. Having access to this probability distribution means that confidence intervals can be calculated. For instance, the 95 percent confidence interval denotes the range estimated to contain the “true” threshold with 95 percent probability.

**Figure 4.**
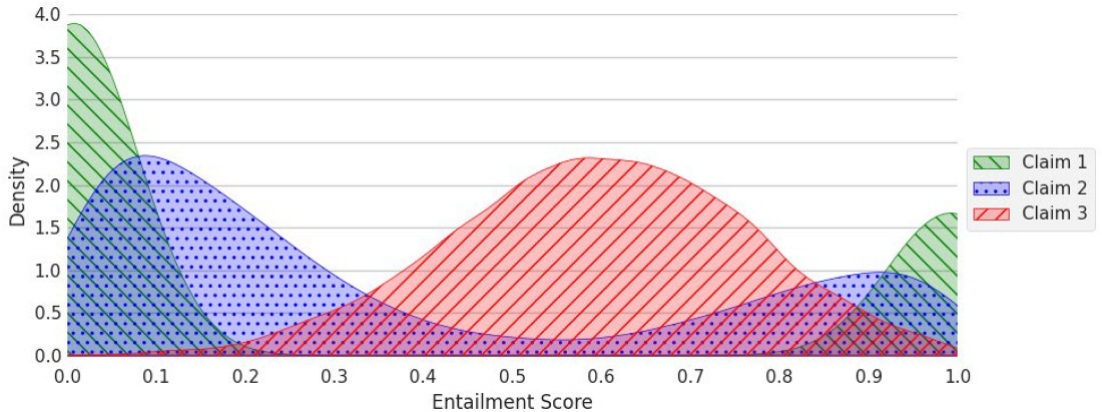
Example distributions of entailment scores.

One might be tempted to simply use the width of the confidence interval around the threshold as an indicator of how well the system performs for a given claim, with narrower intervals suggesting a better performance. However, entailment scores are not typically uniformly distributed between 0 and 1. Figure 4 provides a schematic illustration of this. In practice, we tend to find that the distribution of scores is typically bi-modal, with a peak in frequency near 0 and another (smaller) one near 1, as illustrated by exemplar claims 1 and 2. Other distributions can also exist, however (e.g., exemplar claim 3).

Given such a diversity in possible distributions of scores, the width of a confidence interval can imply very different levels of uncertainty. To see this, take exemplar claim 3. A confidence interval spanning from 0.10 to 0.90 would contain almost the entire dataset for this claim. However, the same confidence interval, despite its width, would contain only a small portion of the data for exemplar claim 1. In other words, a wide confidence interval might not actually be problematic if the corpus contains relatively few data points with scores within this interval. Inversely, a narrow interval might still be problematic if the corpus contains many data points with scores within this interval. In the case of exemplar claim 1, this would, for instance, be the case if the confidence interval spanned from 0.01 to 0.10, or from 0.90 to 0.99.

Rather than relying solely on the width of the confidence interval, the actual distribution of scores in the corpus for a given claim should, therefore, be used to determine what proportion of the data lies within the confidence interval. This proportion can then be used as our third heuristic to determine whether the system is working satisfactorily, with a lower proportion corresponding to a better performance.

The fourth column of Table 2 above summarizes this proportion for the 95 percent confidence interval for each of our claims. We decide to flag claims for which the 95 percent confidence interval contains more than 10 percent of the corpus, namely 1_1 (“Biden is sold out to China”), 1_4 (“Biden works for China”), 1_5 (“The CCP controls Biden”), and 1_6 (“Biden's policies hurt the United States”). It is important to note that this third heuristic need not necessarily be used to rule out these claims. Rather, the confidence intervals can simply be included in claims frequency visualizations, as we will see later.

#### Iterative loop

If a claim does not meet the requirements set out in these three heuristics, the analyst could simply drop it. However, it is more likely that they would want to re-work it, replacing it with one or more claims intended to capture the same idea, and doing so iteratively until the system returns satisfactory results.

Our main recommendation for a successful re-formulation of the claims is simply to refer back to the corpus: that is, to identify “exemplar tweets” from the corpus that make these claims, and to emulate the language used. These “exemplar tweets” may have come up during the initial “corpus discovery” step or during the threshold-tuning: for instance, in the case of claim 1_1 (“Joe Biden is sold out to China”), even if the threshold-tuning was overall unsuccessful, the third, sixth, 10th, 13th, 16th, and 18th examples were all annotated as including the claim (see Figure 3a).

Secondly, if the unsuccessful claim is rather abstract, our second recommendation would be to replace it with more literal or concrete claims. For instance, “Joe Biden is sold out to China” could be replaced by the more specific and literal “Biden accepted bribes from China” or “Biden received money from China in exchange for special treatment.” Similarly, the claim “Biden does business in China” could be replaced by the more specific claim “Biden works with Chinese companies,” “Biden has a bank account in China,” or “Biden owns financial assets in China.”

Finally, the purpose of using an iterative loop in our methodology is not only to improve the quality of the labels, but also to enable a real “social construction,” by the analyst, of the phenomenon of interest. This is consistent with the classic epistemological position proposed by Pierre Bourdieu and co-authors in *The Craft of Sociology* (1991): Objects of sociological investigation are not given—neither by common sense nor by data. Rather, they must be *made* through self-conscious and controlled interventions. This is always crucial, but particularly so when social scientists face an expansive dataset, such as the one we collected here. In this case, we constructed our object by iterating first on the general topic of interest and then on the framing of the representative claims. Our initial taxonomy contained 63 claims, all derived from a manual inspection of a small sample of data. Some of these claims related to COVID (e.g., “China owes reparations for COVID”), trade (e.g., “Cheap Chinese goods put US manufacturing out of business”), geopolitics (e.g., “China wants to annex Taiwan”), or human rights (e.g., “China abuses human rights”). A first iteration over a sample of the data^5^ using our methodology revealed, however, that the prevalence of these claims (at most a few hundred per month) was minimal compared to claims accusing Joe Biden of maintaining an unethical relationship with the Chinese government or Chinese businesses (several thousands of tweets per month, see Appendix F, supplemental materials, for the relevant visualizations). These first results informed our decision to focus exclusively on the latter in our final taxonomy, and to include a comparison with similar claims made about Donald Trump. We do not attempt to re-work this final taxonomy further and simply rule out claims that do not perform satisfactorily. Depending on the goals of a project, other researchers may reasonably choose to rework the taxonomy.

#### Applying the thresholds to obtain the labels

Having iteratively tested and re-worked different claims and calculated their entailment score threshold, we can use these thresholds to classify all the tweets in our dataset as containing or not containing each claim. With each tweet thus given a set of labels, we can generate useful visualizations and statistics to understand the contents of the dataset better. While this can be done for each claim individually, claims can also be combined for further insight. For instance, in the context of our 2020 U.S. presidential elections dataset, one might be interested in visualizing the prevalence of **all** claims about Joe Biden together, or in singling out tweets that contain a particular claim (e.g., “Donald Trump's policies hurt the United States”) but not any other claims. Moreover, the researcher can perform the classification on a greater unit of text than a sentence or paragraph (e.g., an entire news article) using simple inference rules: for example, the article might inherit the claim label if at least one of its sentences/paragraphs has that label.

**Figure 5.**
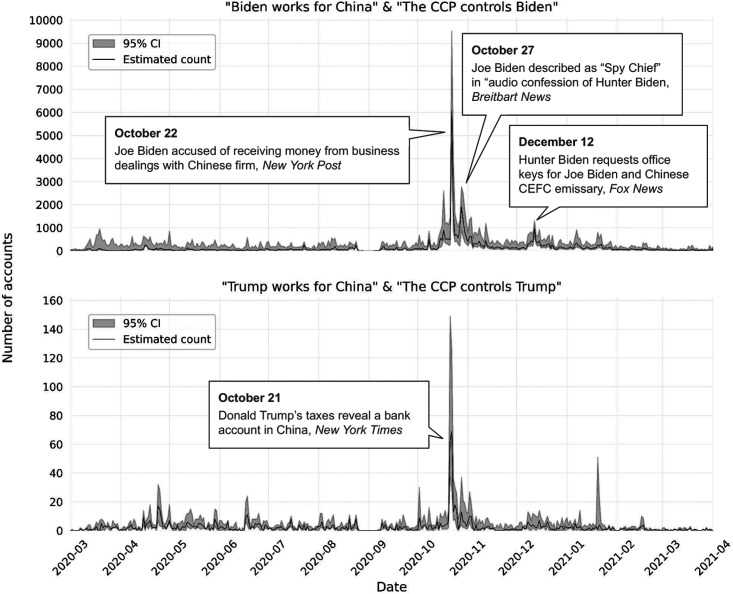
Temporal evolution of a number of accounts posting tweets making claim 1_4 (“Biden works for China”), 1_5 (“The CCP controls Biden”), 2_4 (“Trump works for China”), and 2_5 (“The CCP controls Trump”). The visualization is restricted to March 2020 to April 2024 for clarity, as the activity observed before and after is negligible. The shading shows the 95 percent confidence interval around the threshold for each claim.

In our 2020 U.S. elections case study, Figure 5 shows the number of accounts making claims 1_4 (“Biden works for China”), 1_5 (“The CCP controls Biden”), 2_4 (“Trump works for China”), and 2_5 (“The CCP controls Trump”) over time. We use the number of accounts making the claim as the unit for the *y*-axis, rather than the number of tweets, because it is less sensitive to the excessive tweeting of some accounts (e.g., in one account we found tweeted variations of the same tweet several thousand times in the same day). Moreover, the *x*-axis of these visualizations is restricted to March 2020 until April 2021, as this is the period with the most activity (see Appendix G, supplemental materials, for all other claims and for the activity throughout the 3 years of data collection). Finally, shaded areas on the graphs provide the 95 percent confidence interval around the threshold for each claim.

For both candidates, the idea of a corrupt relationship with China was planted early, in the spring of 2020. When we zoom in on the period immediately preceding and following the election, we see that the claims “Biden works for China” and “The CCP controls Biden” experience their highest peak on October 22, in what may be a reaction to the parallel claims being most forcefully made about Trump on October 21. Although the temporal profiles are similar for the Trump and Biden claims, however, the level of activity is not. The negative claims about Biden far outweigh the claims about Trump, with about 6,000 accounts involved at the peak in mid-October 2020, versus about 60 accounts for claims about Trump. The claims about Biden also linger longer, with a second peak (∼2,000 accounts) on October 27 and 28, and a third one (about 1,000 accounts) on December 12, and continued activity into January 2021. By then, there are still more accounts making negative claims about Biden's relationship to China than at the October 22 peak for Trump.

Inspecting the tweets that make up each of these peaks allows us to identify the specific context associated with each claim. Doing so, we discover that claims about Donald Trump made on the 21st of October are associated with allegations of him having a bank account in China and paying more taxes in China than in the United States, as reported by the *New York Times* (McIntire, Buettner, and Craig 2020). Relating to Joe Biden, the October 22 peak is linked to an article from the *New York Post* in which the candidate is accused of receiving a large sum of money from business dealings involving his son Hunter Biden and “a shady Chinese Communist firm” (Goodwin 2020). The second peak on October 27, on the other hand, is driven by the presumed leak, announced by the website National Pulse, itself founded by the former editor-in-chief of Breitbart News, of an “audio confession of Hunter Biden” in which Joe Biden is described as the “Spy Chief” (Winters and Kassam 2020). Finally, the December 12 peak is tied to a story, reported by Fox News, of an email from Hunter Biden requesting keys made for his new “office mates,” which included both Joe Biden and a “Chinese ‘emissary’ to CEFC chairman” (Singman and Shaw 2020).

### Evaluating our Approach

Earlier, we presented three heuristics for estimating whether or not the methodology we propose works well for a particular claim in the context of a given corpus. In this section, we carry out a more traditional evaluation of the methodology using three human-labeled test sets: the reduced 2020 U.S. elections/China dataset we created for the classification task presented above, and two additional datasets from the literature. This evaluation is not a necessary step for deploying our methodology but it allows us to formally validate it. In what follows, we present each of these test sets and then use them to benchmark our methodology against a range of baselines.

#### Test sets descriptions

We created a sample of 100 tweets for each claim stratified by the entailment score from our 2020 U.S. presidential elections dataset: that is, we picked one tweet per entailment score percentile. This stratification allowed us to sample from the entire range of entailment scores uniformly, unlike a random sampling strategy which would over-represent tweets with scores near the mode(s) of the distribution while under-representing other tweets. In other words, this stratification allowed us to abstract away from the particular distribution of entailment scores in the dataset. As such, the test set is designed to directly test the discriminatory power of the NLI model (i.e., whether, for a given claim, higher scores do indeed correlate with a higher likelihood of an extract including the claim) and of our thresholds (i.e., whether this single threshold is sufficient to distinguish between extracts that do and do not contain the claim).

Having sampled this test set of 1,200 tweets, we hired and trained three undergraduate students to annotate it independently. Note that, because the “threshold-tuning” only needs to be performed once and ideally by the researchers themselves, hiring and training external annotators is not a requirement of our methodology. Rather, we use the annotations from each annotator to validate to what extent our methodology produces labels that align with that annotator's interpretation of the claim. After all, the methodology we put forward does not necessarily strive to provide “objective” claim labels, but rather labels that, per Bourdieu, Chamboredon, and Passeron's (1991) epistemology, fit the intended meaning of the claim according to the researcher: that is, the person doing the threshold-tuning. In other words, the threshold-tuning step of our methodology is precisely intended as an opportunity for the researcher to “infuse” the system with, or “calibrate” it to, their own interpretation of the claim as they *constructed* it through the iterative loop described earlier. A fair evaluation should therefore compare the test set *as annotated by a specific individual* to the claims detection system using the threshold *as tuned by that same individual*. Practically speaking, this means treating the annotations from each annotator as three separate test sets, instead of aggregating them into a single “ground truth.”

The annotators received 2 hr of training before starting the annotation task, during which they were given general instructions on how to approach the task, a definition and a few positive and negative examples for each claim, and the opportunity to practice annotating tweets together (see Appendices H1 and H2, supplemental materials). On average, the annotators spent 9 hr each annotating the 1,200 tweets.^6^ Once this first annotation task was completed, the annotators were also tasked with completing the threshold-tuning for each claim.^7^ It took the annotators on average 3.8 hr to complete the threshold-tuning for all 12 claims. Table 3 summarizes the thresholds obtained by each annotator, the proportion of the test set for each claim annotated as containing that claim, and the number of claims deemed “unsuccessful” according to the heuristic put forward in the previous section. Visualizations representing the results of these two annotation tasks (stratified sample and threshold-tuning) and the exhaustive list of values obtained for these heuristics can be found in Appendix H3, supplemental materials.

**Table 3.** Overview of Test Set and Threshold-Tuning Annotations Provided by Each Hired Annotator.

	Proportion of positive annotations	Average fine-tuned threshold	Number of screened-out claims
Annotator 1	0.23	0.73	5
Annotator 2	0.33	0.70	2
Annotator 3	0.11	0.90	6

From Table 3, we can infer that Annotator 3 was the strictest in their interpretation of the task, as they consistently annotated the least number of examples from the stratified sample as containing the claim and obtained the highest thresholds during the threshold-tuning step. In fact, this annotator was so strict that only six out of the 12 claims passed the three heuristics presented previously according to their threshold-tuning annotations. Annotator 2, on the other hand, was the least strict, consistently labeling more examples from the stratified sample as containing the claim, and obtaining the lowest thresholds during the threshold-tuning step. Finally, Annotator 1 was essentially the median annotator. We therefore used the thresholds obtained by the latter in our step-by-step illustration of our approach earlier.

The fact that this ranking remained mostly consistent for all the claims suggests that disagreements between the annotators can be explained in terms of each annotator's propensity to interpret tweets and claims more or less literally (see Table 4, e.g., illustrating this notion of “literalness”).

**Table 4.** Three Examples from the Test Set Which Annotators 1 and 2 Considered as Making the Given Claims but Annotator 3 Did Not, and Three Examples Which Annotator 2 Considered as Making the Claims but Which Annotators 1 and 3 did not.

Claim	Tweet	Annotator		
		1	2	3
“Biden works for China”	The Communist Chinese are Joe Biden's Puppet Masters. This Socialist Horror will come to America if Joe Biden takes office. As millions of our family members are taken to the gouloggs for execution. <URL>	Yes	Yes	No
“The CCP controls Biden”	@realDonaldTrump Joe Biden & Democrat Party are in Collusion with the Chinese Communists. They're endorsing him out in the open! I wonder how involved are the Chinese COMMUNISTS, the Democrat Party & the "Postal "Vote Mail-in" Scheme? Is Joe Biden a Manchurian candidate? The party will rule!	Yes	Yes	No
“Trump works for China”	BREAKING: Audio of Donald Trump Jr was recorded with Eric Trump admitting to a secret deal with Beijing that implements their father's involvement. Google Spy chief of China tape for a full report. Fact check that Jack <HANDLE> #Biden2020 <HANDLE> <HANDLE> <HANDLE>	Yes	Yes	No
“Biden works for China”	Crazy Joe is a traitor to America! WATCH: Resurfaced Joe Biden Speech Shows Him Urging Chinese Communist Party To Increase Its Influence In The United States <URL> via <HANDLE>	No	Yes	No
“The CCP controls Trump”	<HANDLE> Joe Biden was a respected Senator & VP of the USA. You are just a lying whining ninny. The REAL stories are McConnell is in deep with China, the Trump family is in debt to China, & after all your bitching about Hunter, unqualified you got a Russian board position thanks to Pops.	No	Yes	No
“Trump works for China”	<HANDLE> Its Donald Trump with his China bank account and Ivanka with her 23 Chinese patents.	No	Yes	No

These examples illustrate that Annotator 3 was the most literal and strict in their annotations while Annotator 2 was the least.

In addition to our own test set, we also use test sets from two additional datasets/tasks from the literature. The first is a dataset of news articles from Barberá et al. (2021), where each article is annotated based on whether it describes the economy as performing well (“positive”) or badly (“negative”). Our taxonomy for this task consists of two claims: “The economy is performing well overall” and “The economy is performing badly overall.” In total, we perform 45 annotations to obtain the thresholds for this task (see Appendix I1, supplemental materials, for more details).

The second dataset is taken from Burst et al. (2020) and contains excerpts from party manifestos annotated with one of eight topics: “External Relations,” “Freedom & Democracy,” “Political System,” “Economy,” “Welfare & Quality of Life,” “Fabric of Society,” “Social Groups,” or “Other.” We use the latest version of the dataset at the time of writing (from 2024) and filter out any non-English data. To create our taxonomy, we refer to the coding schema from Burst et al. (2020) and formulate 66 claims in total. Unlike claims for the other two tasks (U.S. elections and sentiment about the economy), claims in this taxonomy are mostly “meta” claims (e.g., “This quote is about the economy”): that is, instead of making a statement, they describe a higher-level property of that text (i.e., the topic). The complete list of claims and their mapping to each topic is given in Appendix I1, supplemental materials. The total number of annotations performed to obtain the threshold for all claims is 1,549 or about 23 annotations per claim on average.

#### Evaluating our method on the test sets

Table 5 summarizes the accuracy and macro-average precision, recall, and *f*1-score of our claims detection approach on each of the test sets presented above (see Appendix I2, supplemental materials, for the equations of accuracy, precision, recall, and *f*1-score and Appendix I3, supplemental materials, for the breakdown of the evaluation per claim). Importantly, as stated earlier, we perform a separate evaluation on the version of our U.S. elections test set annotated by each annotator, where the thresholds used in each case are the ones obtained by that same annotator.

**Table 5.** Performance of Our Proposed Methodology Using the BART_MNLI_ Model and Thresholds Tuned Using the Probabilistic Bisection Algorithm, as Evaluated on the U.S. Elections Test Set Annotated by Each of Our Three Annotators, and the Sentiment and Topic Classification Test Sets. The Precision, Recall and f1-Score Are All Macro-Averaged.

Task		Accuracy	Precision	Recall	f1-score
U.S. elections	Annotator 1	0.76	0.65	0.60	0.60
	Annotator 2	0.76	0.73	0.65	0.65
	Annotator 3	0.87	0.78	0.70	0.72
Sentiment about economy		0.75	0.74	0.74	0.74
Topic in manifestos		0.56	0.46	0.43	0.44

*Note.* BERT = bidirectional encoder representations from transformers; MNLI: multi-genre natural language inference.

Using macro-averaging to calculate the precision, recall, and f1-score for each claim means that the labels “contains the claim” and “does not contain the claim” were both given the same importance irrespective of their prevalence in the test set (or in the entire dataset). This also means that the precision, recall, and *f*1-score values obtained for each task should come close to those that would be obtained if evaluating on the entire dataset. In the case of the U.S. elections dataset, however, it is reasonable to expect that for most claims the accuracy score would be greater if calculated on the entire dataset. After all, as mentioned previously, the distribution of entailment scores in the actual dataset is typically bi-modal, with a peak in frequency near 0 and another one near 1. At these extremes, it is reasonable to speculate that the claim's label is more likely to be correct. Data points with such entailment scores are, however, underrepresented in this evaluation because of the stratification strategy used to sample the test set.

Overall, it is worth noting that the precision is systematically higher than recall (except in the case of the sentiment task where they are equal), implying that our specific implementation of the approach tends to under-detect more than over-detect claims. Moreover, in the case of the U.S. elections data, the performance of our approach appears to be the best when evaluating against the annotations of Annotator 3. This can be explained in several ways. Annotator 3 might have been the most consistent between the two annotation tasks (i.e., on the stratified test set and during the threshold-tuning) in terms of how they interpreted the claims, the tweets, and the notion of whether a tweet is making a claim. Moreover, the BART_MNLI_ model might work best in cases of more “literal” entailment. Using a different NLI model, however, might lead to different behavior.

In isolation, the *f*1-score achieved by our method on each test set cannot easily be used to assess the performance of the method. In what follows, we, therefore, benchmark it against other computational approaches that only require a sample of labeled data before being able to handle unseen examples.

#### Comparing to other computational approaches

##### Fine-tuning pre-trained models

The most salient approach to compare our approach with is arguably a “pre-train/fine-tune” approach such as the one used by Do, Ollion, and Shen (2022), given its prominence in social science research. Estimates of the time required to fine-tune pre-trained LLMs can vary widely, depending on the number of labeled examples used (anywhere from a few thousand to tens of thousands), the complexity of the classification task, how much training annotators require and how much the researcher(s) experiment with data pre-processing, hyper-parameters tuning (e.g., number of training epochs, learning rate, weight regularization, loss function, etc.) and different model architectures. Still, for the sake of comparison, consider that it took an “expert” annotator (i.e., the researcher) 45 hr to annotate the 8,631 data points Do, Ollion, and Shen (2022) used to fine-tune a pre-trained model for just two binary classification tasks, as opposed to 3.8 hr^8^ for our own annotators to complete the threshold-tuning task for all 12 claims. Moreover, these tasks were relatively simple, even when compared to the task of detecting the presence of a single claim in a tweet, as revealed by the average time required for a single annotation (19 s vs. 27 s per annotation^9^).

While fine-tuning a general-purpose transformer model such as BERT is a conventional application of the “pre-train/fine-tune” paradigm, a more innovative approach proposed by Laurer et al. (2024) is to fine-tune an NLI model such as BART_MNLI_. This approach requires the user to formulate “hypotheses” (i.e., the equivalent of our “claims”) and pre-process the data such that each text is concatenated with each claim, before fine-tuning an NLI model like BART_MNLI_ on some of this data. Just like for the base NLI model being fine-tuned, the resulting model then returns a probability distribution over the classes “contradiction,” “neutral,” and “entailment.” For each text, the version of the text with the highest entailment score then corresponds to the predicted claim/class. As demonstrated by Laurer et al. (2024), using an NLI model instead of a general-purpose transformer as the base model for fine-tuning leads to the model learning to perform the classification task quicker, and, therefore, needing fewer training examples. With a large training set, however, the fine-tuned BERT model still performs equally well or even better.

To evaluate a “pre-train/fine-tune” strategy using our own data, we fine-tune both a BERT and a BART_MNLI_ model for each of our three classification tasks. In the case of our own U.S. elections classification task, the training set consists of the sample of tweets annotated by each annotator during the threshold-tuning step, that is, 312, 284, and 236 annotations, respectively. A different BART_MNLI_ model is fine-tuned for each annotator and evaluated on that annotator's test set exclusively. In the case of the BERT model, however, we find that the lack of data when training a separate model for each annotator leads to very poor performance. Instead, we, therefore, use all these annotations together to fine-tune a single BERT model (see Appendices I3 and I4, supplemental materials, for more details on the fine-tuning process). The performance of these baselines on the U.S. elections test set is shown in columns 3 and 4 of Table 6. The fine-tuned BERT model achieves an average *f*1-score of 0.57 across the three human annotators, well below our own approach, while the fine-tuned BART_MNLI_ models achieve an average f1-score of 0.69, that is, 3 percentage points higher than our method. For more information on the fine-tuning process see Appendices I4 and I5, supplemental materials.

**Table 6.** Performance on the 2020 U.S. Elections Test Sets for (1) Our Proposed Methodology Using BART_MNLI_ and Tuned Threshold, (2) a Fine-Tuned BERT Model, (3) a Fine-Tuned BART_MNLI_ Model, (4) Prompting Using BLOOM, (5) Prompting Using Llama 2, (6) Prompting Using Llama 3, and (7) the BART_MNLI_ With a Threshold of 0.5 for all Claims.

	Ours	Fine-tuned BERT	Fine-tuned BART_MNLI_	BLOOM	Llama 2	Llama 3	BART_MNLI_ with a 0.5 threshold
Annotator 1	0.60	0.59	0.69	0.48	0.59	0.56	0.59
Annotator 2	0.65	0.66	0.70	0.43	0.57	0.63	0.62
Annotator 3	0.72	0.47	0.68	0.30	0.58	0.62	0.51
**Average**	**0.66**	**0.57**	**0.69**	**0.40**	**0.58**	**0.60**	**0.57**

*Note.* BERT = bidirectional encoder representations from transformers; MNLI = multi-genre natural language inference.

In the case of the sentiment and topic classification tasks, we have access to much larger training sets from Barberá et al. (2021) and Burst et al. (2020), respectively. Similarly to Laurer et al. (2024), we therefore fine-tune a version of the BERT and BART_MNLI_ models with different amounts of training data ranging from 100 to 2,500 for the sentiment task and 100 to 5,000 for the topic task. This allows us to assess the relationship between training set size and performance when fine-tuning these models.

As shown in Table 7 our method performs the best for the sentiment task, even as the number of annotations for the fine-tuned BERT and BART_MNLI_ increases. For the topic classification task, however, the fine-tuned BERT and BART_MNLI_ model performs the best from about 2,500 (*f*1-score of 0.46 and 0.43, respectively) and continues to learn with more data, albeit at a decreasing rate.^10^ Our own method performs within the same *f*1 range as the two fine-tuned models given the number of annotations performed during the threshold-tuning, but does not outperform these baselines. A plausible explanation for the relatively poorer performance of our method on the topic classification task given the number of annotations might lie in the use of “meta” claims of the form “This quote is about…,” rather than direct statements that can be entailed by the text's content. After all, the MNLI dataset on which BART_MNLI_ is fine-tuned in the first place does not contain training pairs with claims of this form.

**Table 7.** Performance on Two Classification Tasks: Sentiment Classification of News Articles About the Economy (Barberá et al. 2021) and Topic Classification of Excerpts From Party Manifestos (Burst et al. 2020). The Different Approaches Evaluated Are (1) Fine-Tuned BERT Models With Varying Amounts of Training Data, (2) Fine-Tuned BERT_MNLI_ Models With Varying Amounts of Training Data, (3) Prompting Using Llama 3, (4) Zero-Shot Classification Using the BART_MNLI_ Model With a Default Threshold of 0.5, and (5) Our Proposed Methodology Using BART_MNLI_ With Tuned Thresholds.

Approach		Sentiment about economy	Topic in manifestos
Fine-tuned BERT models	BERT + 100 annotations	0.37	0.10
	BERT + 500 annotations	0.44	0.30
	BERT + 1,000 annotations	0.58	0.37
	BERT + 2,500 annotations	0.62	0.46
	BERT + 5,000 annotations		0.48
Fine-tuned BART_MNLI_ models	BART_MNLI_ + 100 annotations	0.67	0.39
	BART_MNLI_ + 500 annotations	0.68	0.42
	BART_MNLI_ + 1,000 annotations	0.71	0.43
	BART_MNLI_ + 2,500 annotations	0.71	0.43
	BERT + 5,000 annotations		0.51
Llama 3		0.66	0.37
BART_MNLI_ with a 0.5 threshold		0.68	0.40
Ours		**0.74**	0.44

*Note.* BERT = bidirectional encoder representations from transformers; MNLI = multi-genre natural language inference.

##### Prompting

Aside from “pre-train/fine-tune” strategies, another paradigm that has become very popular for text classification tasks in the field of NLP in the last few years is “prompting” (Liu et al. 2023). This approach leverages generative language models which, as their name indicates, are designed to generate text, for instance, for question answering or text completion. At the time of writing this article, Meta's Llama 2 (Roumeliotis, Tselikas, and Nasiopoulos 2023) and Llama 3 (AI@Meta 2024) models are prominent open-source text completion tools built from a generative language model. Another notable (though slightly older) choice is BigScience's BLOOM model (Scao et al. 2022). “Prompting” then refers to the practice of giving a “prompt” to the generative model such that its output is the classification label. The model's output, that is, the “answer” to the question in the case of question-answering models or the completion of the prompt in the case of text completion models, would then correspond to the label for that claim: for example, “Yes” if a tweet contains a specified claim, “No” otherwise. We compare the performance of our own claim detection approach to a prompting approach using Llama 2, Llama 3, and BLOOM, where the prompt used is the following:


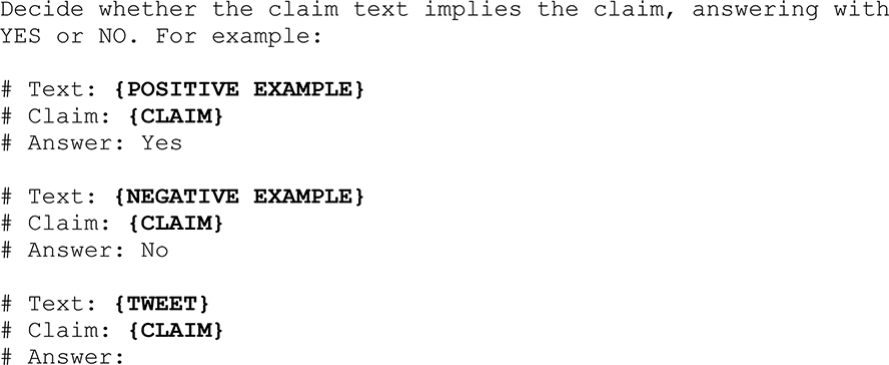


Here, the **{TWEET}** placeholder corresponds to the tweet to label, **{CLAIM}** corresponds to the claim to label it with, and **{POSITIVE EXAMPLE}** and **{NEGATIVE EXAMPLE}** are predefined positive and negative examples for that claim^11^ (see Appendix I6, supplemental materials, for the exhaustive list of examples given as context). For the 2020 U.S. elections classification task, we summarize the performance of these three models according to the annotations provided by each annotator in columns 5–7 of Table 6. Our methodology using the BART_MNLI_ model and tuned thresholds significantly outperforms Llama 2, Llama 3, and BLOOM, by 8, 6, and 12 *f*1-score percentage points, respectively. Llama 3 being the best-performing of the three models, we evaluate it on the sentiment and topic classification tasks as well. As shown in Table 7, the model achieves an *f*1-score of 0.66 on the sentiment classification task, thus performing better than all fine-tuned BERT baselines but worse than other approaches, including fine-tuned BART_MNLI_ baselines and our own method. Similarly, it performs better than BERT models fine-tuned on 1,000 examples or less on the topic classification task with an *f*1-score of 0.37 but worse than any other approach, including our own.

##### Zero-shot classification using BART_MNLI_

Finally, to validate the benefits of the threshold-tuning step of our method, we also compare the performance of our system to a version that uses a unique threshold of 0.5 for all claims (i.e., a “zero-shot” approach). This information is summarized in column 8 of Table 6 for the U.S. elections task, and in the “ZSL w. BART_MNLI_” row of Table 7 for the sentiment and topic classification tasks. Our method using the BART_MNLI_ model with fine-tuned thresholds outperforms this third baseline as well, by 9, 6, and 4 *f*1-score percentage points, respectively.

#### Threshold-tuning across different folds of data

At a given timestep of running the probabilistic bisection algorithm for threshold-tuning, several data points can have an entailment score equal to the current median of the probability distribution and therefore be selected for annotation. This raises the question of how dependent the thresholds are on the specific examples annotated: that is, whether these thresholds might be “over-fitting” the small training set used to obtain them. In this section, we follow a strategy proposed by Chausson and Ross (2024) to evaluate the extent to which this is the case: we split our 2020 U.S. elections dataset into three folds of equal size (378,428 data points per fold) and run the threshold-tuning independently on each fold. We then calculate the difference in the obtained thresholds.

**Figure 6.**
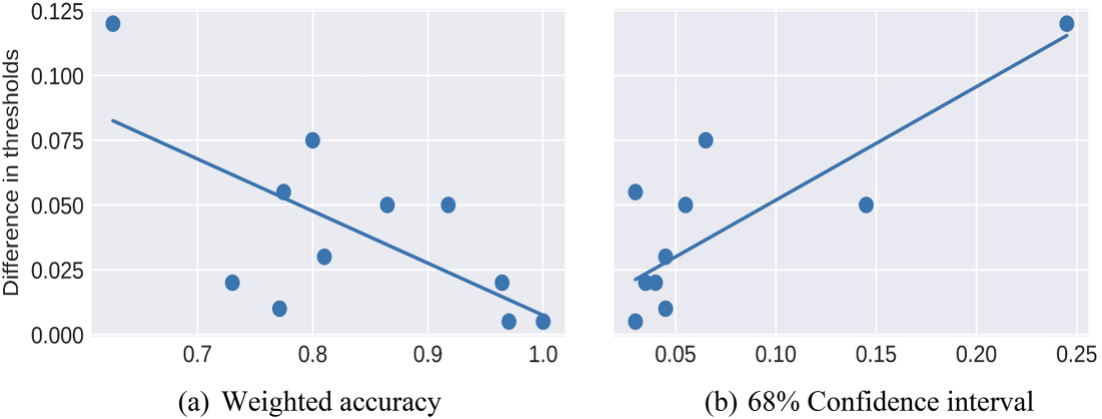
Difference between the highest and lowest threshold per claim when tuning thresholds on three separate folds of the U.S. 2020 presidential elections dataset, shown against the weighted accuracy per claim and width of the 68 percent confidence interval (averaged across all three folds).

For the 12 claims used for our U.S. elections classification task, differences in thresholds range between 0.005 and 0.12, for an average of 0.037 and a standard deviation of 0.03. Interestingly, plotting the difference in thresholds per claim against their “weighted accuracy” (as specified in the “Heuristic 2” section) averaged across folds reveals a negative relationship between the two variables (see Figure 6a). Similarly, plotting the difference in thresholds per claim against the average width of the 68 percent confidence interval (i.e., first standard deviation) reveals a positive relationship (see Figure 6b). These results indicate that slight differences in thresholds can be expected when selecting a different set of examples to annotate during the threshold-tuning step. Importantly though, the weighted accuracy heuristic and the width of confidence intervals around the thresholds are informative for estimating the trustworthiness of the thresholds. Moreover, as explained under the “Heuristic 3” section above, the impact of diverging from the “ideal” threshold on the number of misclassifications will also depend on the actual distribution of entailment scores for a given claim: that is, a wide confidence interval might not be as problematic if the corpus contains few data points with entailment scores within that interval.

## Discussion and Conclusion

The rapid growth in the availability of large-scale textual data, coupled with the development of computational algorithms to analyze those data, presents both opportunities and challenges for social scientists. In this article, we present and illustrate a methodological framework based on NLI to code large datasets. This methodology can be used to detect the presence of statements in text and, insofar as these can denote higher-level concepts (e.g., sentiments and stances), to classify text into more general categories. Moreover, it only requires a few human-annotated examples for each statement and provides an explicit strategy for sampling these annotations.

The efficiency gained from this approach facilitates an iterative workflow, which we describe as an “insight-inference loop.” Researchers interact productively, frequently, efficiently, and *analytically* with algorithms while working with a large corpus of text data. An important benefit of this efficiency is that it allows us to avoid three problems that have made many social scientists suspicious of the automation of computational text analysis: sidelining decades of social science evidence and theory, excluding researchers without the resources to develop large training datasets, and erroneously relying on ‘black-box’ algorithms to make analytical decisions based on inscrutable foundations.

Our approach is structured through five steps, although any particular analysis will likely need to iterate over the steps multiple times to improve the labeling of text. After defining, assembling, and cleaning the corpus, the researcher first elaborates a taxonomy of claims—short sentences that capture the statements, concepts, or ideas that the researcher wishes the algorithm to code in the text. This step can be done inductively, deductively, or through a combination of the two, drawing on prior research, theory, and a careful examination of the corpus. The taxonomy can be revised based on insights from later steps. Next, the researcher deploys a pre-trained NLI model to generate an entailment score, indicating the probability that a document contains a claim of interest. The use of a pre-trained model in this step is a key source of efficiency (note that our framework can accommodate any NLI model, allowing researchers to adopt improved models as they become available). In the third step, the researcher codes a small number of documents for each claim to determine the threshold for the distribution of entailment scores. This “few-shot” and “active learning” threshold tuning step allows the social scientist to intervene efficiently, using the probabilistic bisection algorithm to examine a relatively small number of documents per claim. Through this process, the algorithm “learns” the researcher's substantive interests and preferences. Following this, the researcher evaluates the classification performance using key metrics from the previous coding step. Based on this evaluation, they can revise, reconceptualize, or remove poorly detected claims, informed by theory and a close reading of the documents used in the previous step. The researcher iterates between these two steps until they are satisfied that the complete set of desired claims is performing well. Finally, the researcher applies the NLI model with the final claims and chosen thresholds to label the entire corpus, enabling subsequent analysis.

We illustrated the use of our approach with an analysis of a novel Twitter corpus about China and the 2020 U.S. election. Labeling 1,310,540 tweets with 12 claims allowed us to document the pattern of tweets about China over time relative to election-related events. Although space prevents us from providing a full treatment here, the analysis offers interesting insights about the centrality of China as an imaginary villain in recent U.S. politics. China emerges from the data as a wedge issue, a geopolitical rival bent on controlling U.S. politicians, and by extension the U.S. government, for its own ends, and in direct threat to freedom and democracy. It is essentially invoked to malign the Democratic Presidential candidate's reputation, accused of being insufficiently tough or inherently corrupt. While we did find negative mentions of China in relation to Donald Trump as well, these pale in comparison with the incessant flood of negative tweets seeking to compromise Joe Biden and especially his son Hunter, through a generic association with Xi Ji Ping, the Chinese state, the Chinese Communist Party, or Chinese businesses. Importantly, the peak of this activity was found to have occurred on October 22, 2020, a mere 2 weeks before the U.S. presidential election but immediately following the publication of a *New York Times* article about Donald Trump's own business dealings in China, where one of his companies maintained a bank account (McIntire, Buettner, and Craig 2020). This kind of fine-grained insight would have been hard to spot without a computational approach. We plan to dig further into these patterns and present a more in-depth and systematic analysis of our data in a future contribution.

The generalizability and performance of our proposed method were validated against several baselines on three different tasks: detecting claims relating to China in a corpus of Twitter data about the 2020 U.S. presidential elections, classifying news articles based on their sentiment about the economy, and classifying manifesto excerpts based on their main topic. As summarized in Table 8, our proposed approach has a number of advantages over existing computational text analysis workflows. First, it performs well, better than “prompting” or “zero-shot” approaches and about as well as BERT or BART_MNLI_ models after fine-tuning. Moreover, it requires significantly less annotated data than fine-tuning a BERT model to achieve this performance, while providing an explicit strategy for sampling the training examples. Finally, the method does not require the researcher to actually fine-tune any model, making it computationally and technically more accessible. These advantages translate into significant benefits for researchers. Time spent annotating data and fine-tuning a model can limit the researcher's ability to inductively discover and revise key concepts and measures. In comparison, the relative efficiency of our approach, both in terms of computation and training data, shifts the use of researcher time toward the analytically important tasks of generating, revising, and evaluating claims by iterating between computation and close reading. Moreover, this iterative process also provides the researcher with greater intuition as to how the models are working to label the corpus.

**Table 8.** The Main Strengths and Weaknesses of Various Computational Approaches to Textual Classification Evaluated in This Articles.

Approach	Citation	Strengths	Weaknesses
Fine-tuning general-purpose LLM (e.g., BERT)	Do, Ollion, and Shen (2022)	Works well for any textual classification task given enough good quality training data.	Requires significant amounts of training data. Technical overhead of fine-tuning the pre-trained model.
Fine-tuning NLI model (e.g., BART_MNLI_)	Laurer et al. (2024)	Require significantly less annotations than when fine-tuning a BERT model.	Technical overhead of fine-tuning the pre-trained model.
Prompting with generative models	Liu et al. (2023)	Requires only a couple of examples for in-context learning. Does not require any fine-tuning.	If not open-source (i.e., available via an API), issues wrt interpretability and data privacy. Smaller open-source models exhibit poor performance. Larger open-source models too large to run on standard hardware.
Zero-shot classification with NLI model	Yin, Hay, and Roth (2019)	Requires no training data. Does not require any fine-tuning.	Domain shift between pre-training and inference limits performance.
NLI model with threshold-tuning (ours)		Does not require any fine-tuning. Require significantly less annotations than when fine-tuning a BERT model. Performs better than Prompting or Zero-shot strategies. Provides strategy for selecting training examples.	Works less well for “meta” claims (e.g., “This quote is about the economy”).

*Note.* LLM = large language model; BERT = bidirectional encoder representations from transformers; NLI = natural language inference; API = application programming interface.

Our work on this article occurred amid the introduction and widespread adoption of massive, generative models from private companies (e.g., OpenAI's ChatGPT) in many aspects of work and personal life. One might reasonably ask whether our approach is superseded by the availability of those tools, which could be simply fed documents, and asked whether each claim is present in the document. While someday such an approach might be advisable, we believe there are multiple reasons that social scientists should be cautious about such tools. One is that many of these models are proprietary. This means that the underlying models are not available to researchers to understand and refine. In short, they magnify the “black box” problem. Second, they do not currently produce true classification probabilities (or what we call entailment scores), and it is, therefore, hard to develop metrics for evaluating how well the model is working or to fine-tune its use. Third, researchers should be able to run these models in their own secure computing environments in order to protect their subjects’ privacy and confidentiality. For example, we do not recommend feeding proprietary or sensitive data into the public-facing versions of closed LLMs, for legal and ethical (e.g., privacy) reasons. Some corporate tools may offer more guarantees with respect to safeguarding data, but they may be prohibitively expensive for researchers. Finally, model size is an issue. While we did experiment with open-source models in this paper (Llama 2, Llama 3, or BLOOM), we could only deploy the smallest version of these models in our environment. These smaller versions performed worse than other computational classification methods, including our own.

Our evaluation suggests that our approach might be easiest to use and most accurate when claims are more literal, although our example of views of China around the 2020 election in Twitter data shows that non-literal meanings can be detected as well (e.g., “Trump is sold out to China”). As with almost any computational approach, the approach is also likely to work best when the goal is to aggregate the coded documents by other variables, as we have done in our U.S. elections case study in aggregating tweets in time, rather than in use cases in which there are a small number of documents in the corpus and each must be coded without error. Our analysis takes advantage of the law of averages to detect aggregate patterns, even when a fraction of the documents may be incorrectly coded. Moreover, we expect that the approach should work best on more structured and edited text, such as newspaper articles, books, and even blog posts. Indeed, social media corpora, such as the Twitter dataset used in our case study, are especially challenging for computational classification methods due to their frequent use of rapidly evolving colloquial language, incomplete sentences, non-verbal signs, and the absence of punctuation.

Finally, our evaluation suggests that the approach works best with claims that express direct statements (e.g., “The economy is performing well overall”) rather than “meta” claims about the text itself, such as “This quote is about the economy.” In other words, our approach is particularly suited for labeling textual data based on its explicit affirmations, that is, based on what it *says* rather than higher-level properties of that text, like language, tone, topic, or “truth-value.” Most straightforwardly, this can be explained by the fact that NLI datasets, such as the MNLI dataset that the BART_MNLI_ model was fine-tuned on (Williams, Nangia, and Bowman 2017), do not typically contain training examples with such ‘meta-claims’.

On a deeper level though, this finding also aligns with our understanding of human communication patterns. In natural discourse, the context in which people utter meaning is rarely made explicit. Instead, humans rely on shared and implicit understandings. When discussing the economy, for example, one typically does not preface their statement with “this sentence is about the economy.” Such a clarification is unnecessary because the context is implicitly understood. Instead, nearly all the verbal energy in human communication is focused on making direct statements about things or people in the environment (in this case the economy). This phenomenon is closely related to what socio-linguists call the “indexicality” of language (Goffman 1983; Gumperz 1982). Simply put, indexicality refers to the notion that the meaning of certain words or phrases depends on the context in which they are used, a context that is often left unstated (e.g., demonstrative pronouns, such as “this,” are indexical). This taken-for-granted nature of human communication might pose significant challenges for machine learning models, which are trained on available human-produced text. Precisely because explicit references to context are infrequent in human communication, the training datasets that LLMs learn from during their pre-training are likely to contain far more instances of direct statements about, say, economic conditions than meta-linguistic statements about the nature of the texts themselves.

Far from undermining its utility, these insights help scope possible applications of our approach. By focusing on the specific meanings of communicative actions, such as tweets, rather than the meta-topics and sentiments that these actions may reflect, we can stay closer to the empirical ground and the world that the participants inhabit. This makes our approach a particularly useful tool for the sociology of culture. By focusing our analysis on meaningful phrases (rather than simply topics or sentiments), similar to the ones that might be used in actual communications, we can capture the kinds of semantics that matter to the formation and maintenance of symbolic universes and political attachments. For instance, in the context of our empirical illustration using a Twitter dataset about the 2020 U.S. elections, the discursive association between Biden, China, and allegations of corruption (which we capture by the generic phrase “Biden is sold out to China” but appears in various related forms) emerged in our initial inspection of the data as a shorthand designed to solidify negative perceptions about the Democratic presidential candidate. Importantly, this narrative was not isolated to social media; it was part of a broader, coordinated effort to shape public beliefs. Trump himself frequently called Biden “China Joe.” Influential media figures on the right further echoed and amplified the message. On Fox News, for instance, “host Tucker Carlson made a regular habit of accusing his political opponents of literally working for the Chinese government” (Marcetic 2023). It is in this vernacular form that powerful cultural tropes circulate and gain resonance across platforms. By staying as close as possible to the actual language people use, our strategy of inference better preserves the original, qualitative “insights” we started from and helps convey the emotional resonance and cultural significance these associations might hold for both the senders and the recipients of these messages, up to the point where they become fully taken-for-granted.

## Supplemental Material

sj-pdf-1-smr-10.1177_00491241251326819 - Supplemental material for The Insight-Inference Loop: Efficient Text Classification via Natural Language Inference and Threshold-Tuning Supplemental material, sj-pdf-1-smr-10.1177_00491241251326819 for The Insight-Inference Loop: Efficient Text Classification via Natural Language Inference and Threshold-Tuning by Sandrine Chausson, Marion Fourcade, David J. Harding, Björn Ross and Grégory Renard in Sociological Methods & Research

## Notes

1. For instance, these algorithms are known to sometimes rely on spurious associations in the training data, rather than relevant features. This can lead to very different performance when the model is deployed on data that does not resemble the training data.

2. Kozlowski et al. (2019) exploited the properties of word embeddings in their study of the “geometry of culture.” However, instead of using the embeddings as input into a separate classification model, the authors analyze the embeddings directly.

3. This includes tweets as well as retweets, quotes, and replies.

4. The algorithm might terminate early if no unannotated data point can be found close enough to the median.

5. 10,000 tweets per month from February 2020 to July 2021.

6. The Cohen's kappa coefficient obtained from the three annotators’ test set annotations is 0.44, indicating a moderate agreement between annotators. Given that annotations from each annotator are treated as three separate test sets, however, the significance of this metric is limited.

7. Training examples were excluded from both annotation tasks and the stratified sample was excluded from the dataset for the threshold-tuning.

8. We exclude both the time required to run the NLI model (for our approach) and that required to fine-tune and run the pre-trained model (for a traditional “pre-train/fine-tune” approach) in this comparison.

9. The lack of expertise of our student annotators could have contributed to their taking longer to annotate.

10. The f1-score might appear low, however it is worth noting that, for each prediction, only one of the eight possible labels can be considered correct. This makes the problem much more complex than the two other (binary) classification tasks we are evaluating on. After all, instead of a 50 percent chance, a random guess would only have a 12.5 percent chance of being correct. Moreover, this particular task is relatively subjective and complex. While in theory an excerpt should be labeled as relating to only a single topic, in practice the same excerpt can relate to several topics in equal measure and the annotation schema does not always clarify which topic should be considered the dominant one. In other words, while the f1-score is useful to compare across classification methods on the same dataset, it should be taken with a pinch of salt when considered on its own to decide whether the model is good enough to be deployed.

11. Providing training examples in the prompt is called “in-context” learning and can be considered a “few-shots” approach.
