# Supplementary material for: The Insight-Inference Loop: Efficient Text Classification via Natural Language Inference and Threshold-Tuning
Source: Sociol Methods Res. 2025 Apr 18;55(2):568–615. doi: 10.1177/00491241251326819 (PMC13038153; doi:10.1177/00491241251326819)
Supplement: sj-pdf-1-smr-10.1177_00491241251326819 - Supplemental material for The Insight-Inference Loop: Efficient Text Classification via Natural Language Inference and Threshold-Tuning [file sj-pdf-1-smr-10.1177_00491241251326819.pdf]

Appendix: “The Insight-Inference Loop: Efficient Text Classification via Natural Language Inference and Threshold-Tuning”

A. Twitter API and data collection

We used version 1.1 of the Twitter API to collect our data. At the start of our data collection process, this version of the API was the only one available: version 2.0 of the API, with further capabilities destined for academic research specifically, did not yet exist[1]. Moreover the Twitter API at that time was still free. We notably made use of the “streaming” (also called “firehose”) feature of the Twitter API. This feature of the Twitter API returns tweets that match the query as they are published in real time, as well as their retweets, quotes and replies. In other words, the data returned using the Streaming API strictly corresponds to the time window for which the data collection is running. The main advantages of this method are that 1) the data collection is not impacted by future content moderation or the deletion of Twitter user accounts and 2) the metadata of collected tweets (e.g. user information) strictly corresponds to their state at the time of the Tweet’s publication. Disadvantages, however, include 1) the loss of data if the data collection process is interrupted and 2) the inability to evaluate the final impact of tweets (e.g. number of retweets, replies and quotes) directly from the collected data, as these are by nature 0 (or close to 0) when picked up by the API .

We launched two data collection processes using the Streaming feature of the API, each using the following queries:

**Process 1**	"Donald Trump", "Elizabeth Warren", "Bernie Sanders", " Joe Biden", "Andrew Yang"
**Process 2**	"Kamala Harris", "Pete Buttigieg", "US election", "Democratic candidate", "Republican candidate", "2020 United States presidential election", "presidential re-election"

The split into two processes only served to balance the expected volume retrieved from the Twitter API. Once this data was collected, it went through several filtering steps during the analysis. These filtering steps, and how each filtrate was used, is summarized in the following

[1] Twitter released in 2020 a v2.0 of its API, which does not restrict academic researchers on how far back in time they can collect data from and which provides higher quotas per query. This version of the API, however, was released after we first started collecting data. In an effort to keep the data collection process consistent, we therefore decided to continue using v1.1 of the Twitter API.

Figure. It is worth noting that retweets, quotes and replies returned by the Twitter API also included by default the original tweets they related to, so each of these can be counted as two tweets in our dataset.

Data processing step	Method
1. Collect data using Twitter API	***Twitter API***: *see Table for keywords*
2. Filter for subset of keywords	***Token matching***: *"Donald Trump", "Bernie Sanders", "Joe Biden", "US election", "Democratic candidate", "Republican candidate", "2020 United States presidential election", "presidential re-election"*
3. Keep only mentions of China	***Token matching***: *"china", "chinese", "kungflu", "kung flu", "asian", "wuhan"*
4. Keep only English tweets	**`langdetect`** ***Python package***: *uses Google's language detection Java library under the hood*

→ Claims detection

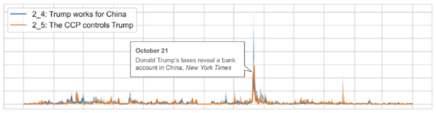

B. Manual review of data samples

The following table contains the most prominent claims identified from a manual review of a sample of the data.

China owns Joe Biden<br>Hunter Biden has deals with China<br>Joe Biden's son doing business in China<br>"China Joe"<br>"Joe China"<br>"Beijing Biden"<br>The Biden/Xi deal<br>Biden quotes Mao<br>Biden is a traitor<br>Biden will sell US to China

- Biden is a sell out
- Biden sold out to China
- Biden is controlled by the Chinese Communist Party
- Biden is compromised by Chinese Communist Party
- Biden is an agent of the Chinese government
- Biden taking cash from China
- Biden makes money in China
- Biden is laundering money in China
- China pays Biden to steal election
- China supporting Biden
- Biden Chinese puppet
- Biden lied about China connections
- Biden partnership with China
- Biden family compromised by the Chinese government
- Biden, China and Ukraine
- China blackmails Biden
- Biden is corrupt
- Biden colludes with China
- Biden works for China
- Biden bought by China
- Biden got rich off China
- Biden favors the Chinese
- Laptop evidence

China owns Donald Trump
- Trump owes money to China

China is interfering in US election in favor of Biden
- ...Meddling
- ...Disrupting
- ...Influencing
- The American Republic vs the CCP
- Documentary exposes China’s game plan
- millions of votes coming from China to help Joe Biden win
- ballots printed in China
- China funded the riots
- China steals US election
- China election fraud
- Beijing ‘Substantially Involved’ in US 2020 Election
- China owns the Dominion voting system
- The CCP virus
- Hundreds of Twitter accounts linked to China

US intelligence/officials/White House say that China, Russia and Iran are interfering in US elections

- China, Russian and Iranian hackers target US elections
- Cyberattacks from China, Russia and Iran
- China sought to influence US election according to Director of National Intelligence
- China sowed disinformation

US Intel Report: 'Russia interfered in election to hurt Biden, help Trump; China didn't interfere'

- report reveals China did not try to subvert US election

China says it has no intention to interfere in US election
China congratulates Biden on election

Presidential election is a contest between US and China

- US and Chinese Communist Party

China created coronavirus to weaken Trump

- destabilize/bring down

Coronavirus will destroy Trump's great economy

- ruin

China steals American jobs

- China prospers at the expense of US

Trump will hold China accountable for coronavirus

- Trump will sue China over coronavirus
- Trump will bill China for coronavirus

The Chinese virus

- Kung flu

- Biden is mentally compromised
- Biden has dementia

Biden is soft on China

- Biden is weak
- Biden is spineless
- Biden [signed executive order to] ban the term "China virus"
- Biden says Chinese genocide is cultural norm
- Biden defends China

Trump stands up to China
- Trump is strong on China

Trump blames China and WHO for Covid deaths

Biden supports rioters and domestic terrorists and China

Biden is fascist
- a dictator

Trump is racist

Trump sought help from Xi for reelection
- [Bolton says] Trump asked China for election help
- Trump sold out to China
- Trump kowtows to China
- China supports Trump’s reelection
- China is rooting for Trump
- Trump in bed with China

Facebook removes fake Chinese accounts
- Fake drivers’ licenses from China
- Counterfeit drivers’ licenses from China

Twitter censors the movie Obamagate

The Democrats stole the election

CCP Cyberware attack linked to McConnell’s wife

US criticizes China over Hong Kong
- China hits back at US criticism of Hong Kong election change.
- China passes Hong Kong election law
- China destroying democracy in Hong Kong

Texas GOP candidate says she does not want Chinese immigrants

Biden revokes ban on Tiktok and WhatsApp

Chinese lab theory is true

These claims were then synthesised into the following list, which served as our initial taxonomy of claims.

```
1_0: 'China is responsible for COVID',
        '1_1': 'COVID originated in Wuhan lab',
        '1_2': 'China created COVID as a biological weapon',
        '1_3': 'China owes reparations for COVID',
        '1_4': 'Trump will sue China over COVID',
        '1_5': 'Trump will bill China over COVID',
        '1_6': 'China should pay over COVID',
        '1_7': 'China should be boycotted because of covid',
        '1_8': 'The United States funded the Wuhan lab',
        '1_9': 'China underreports COVID cases',

'2_0': 'Joe Biden is sold out to China',
        '2_1': 'Biden does business in China',
        '2_2': 'China Joe',
        '2_3': 'Joe China',
        '2_4': 'China blackmails Biden',
        '2_5': 'Biden works for China',
        '2_6': 'Biden works for the CCP',
        '2_7': 'The CCP controls Biden',
        '2_8': 'Biden is weak',
        '2_9': 'Biden is weak on China',

'3_0': 'Donald Trump is sold out to China',
        '3_1': 'Trump does business in China',
        '3_2': 'China blackmails Trump',
        '3_3': 'Trump works for China',
        '3_4': 'Trump works for the CCP',
        '3_5': 'The CCP controls Trump',

'4_0': 'China helps Biden win US election',
        '4_1': 'China stealing US election for Biden',
        '4_2': 'China favors Biden in US election',
        '4_3': 'China funded the riots',
        '4_4': 'China created coronavirus to help Biden win',
        '4_5': 'China created coronavirus to weaken Trump',
        '4_6': 'US election ballots printed in China',
        '4_7': 'Coronavirus will destroy Trump’s great economy',

'5_0': 'China cannot be trusted',
```

'5_1': 'Chinese hackers are stealing covid research',
'5_2': 'China is covering up for the origins of COVID',
'5_3': 'Chinese case counts are dubious',
'5_4': 'China underreports COVID cases',
'5_5': 'China is an authoritarian state',
'5_6': 'China abuses human rights',
'5_7': 'China is using COVID as an excuse for persecuting minorities',
'5_8': 'China is using COVID as an excuse for oppressing people',
'5_9': 'China uses technology for surveillance',
'5_10': 'Chinese PPE is bad',
'5_11': 'China is a xenophobic and/or racist country',
'5_12': 'China’s response to COVID is too repressive',
'5_13': 'China creates disinformation',
'5_14': 'China wants to annex Taiwan',
'5_15': 'Chinese people are cruel to animals',
'5_16': 'China is not cooperating with the international community',

'6_0': 'China is too powerful',
'6_1': 'China steals jobs',
'6_2': 'Cheap chinese goods put US manufacturing out of business',
'6_3': 'China controls the US economy',
'6_4': 'China’s international influence is growing',
'6_5': 'Chinese tech collects data on US citizens'

'7_0': 'Blaming China for COVID is racist or xenophobic',
'7_1': 'Trump is unfairly blaming China for COVID',
'7_2': 'Trump is racist',

'8_0': 'China helps Trump win US election',

'9_0': 'Trump is strong',
'9_1': 'Trump is strong on China',

C. Entailment scores distributions

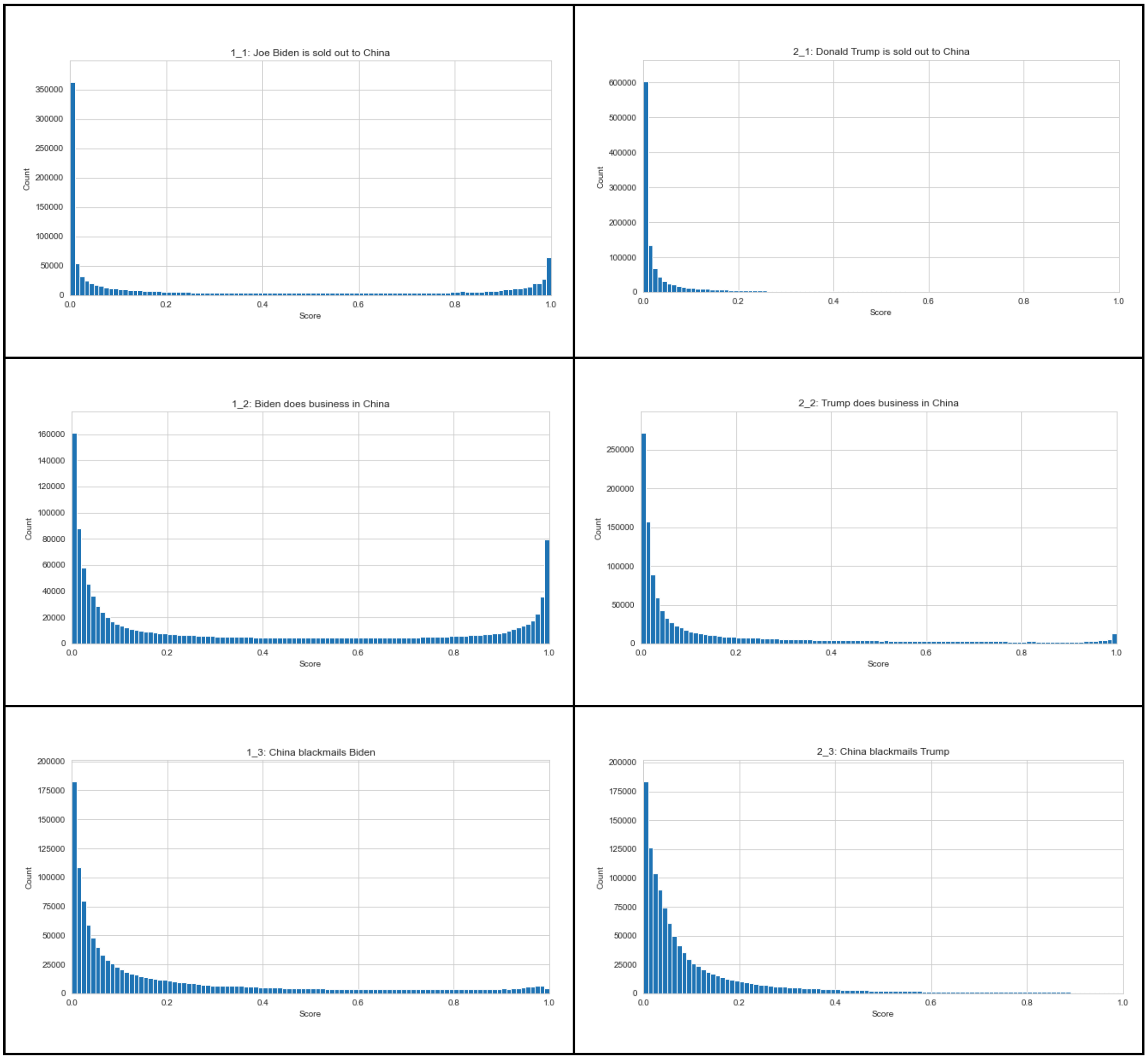

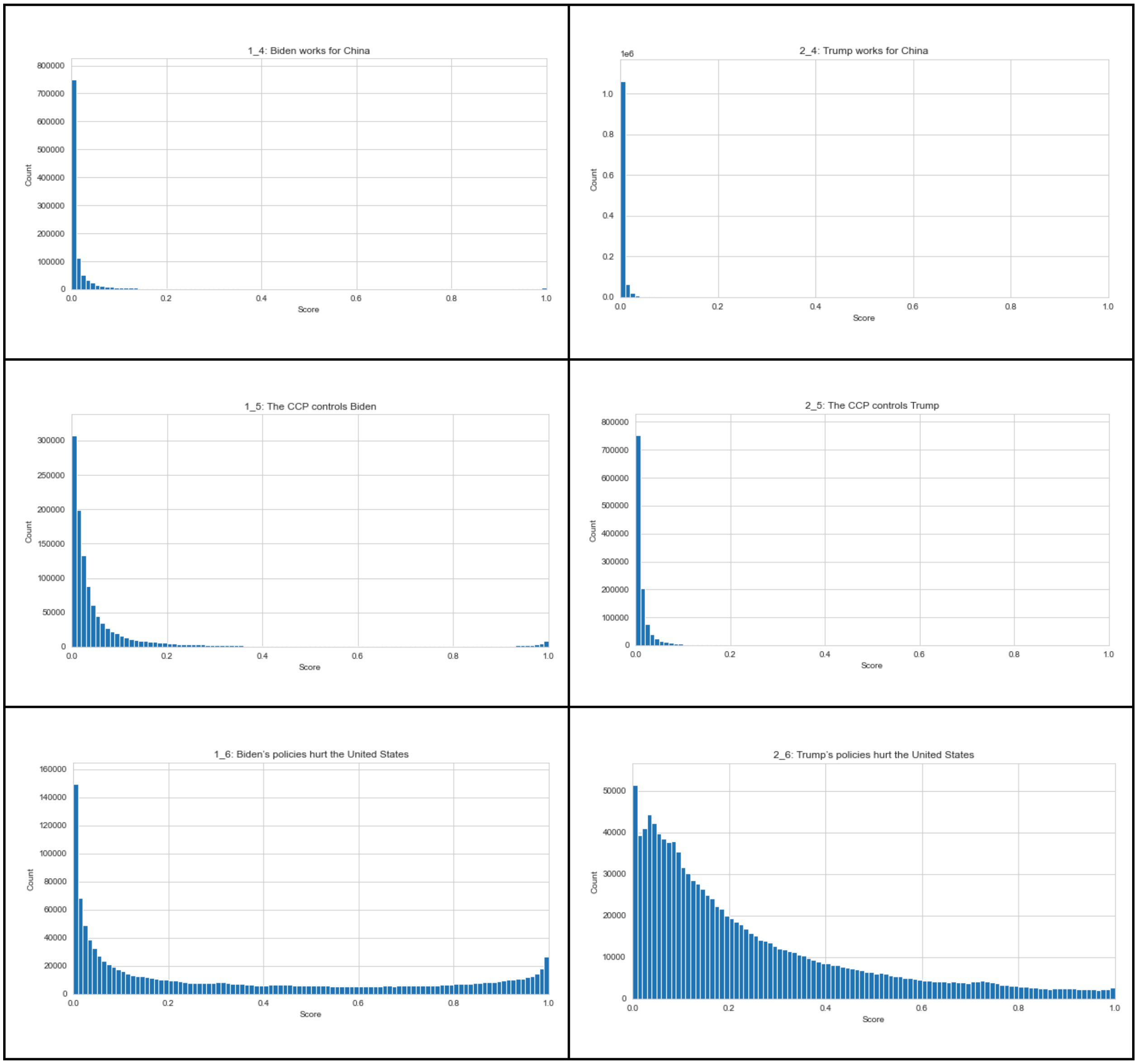
1_4: Biden works for China
2_4: Trump works for China
1_5: The CCP controls Biden
2_5: The CCP controls Trump
1_6: Biden's policies hurt the United States
2_6: Trump's policies hurt the United States
Count
Score
1e6

D. The Bisection Algorithm

We start by assuming that the location of the threshold *S** is represented by a uniform distribution f_0 spanning the entire 0 to 1 range. We then select the median point of this distribution, which at timestep 0 we call m_0 and is equal to 0.5. The algorithm then samples a tweet with an NLI score s_0 equal to m_0 for the user of the system to annotate. If the user annotates this tweet as **not** implying the claim, we can infer that the optimal threshold *S** we are looking for will probably be **higher** than s_0 with a probability *p*, where *p* is a hyperparameter. We therefore update the probability distribution so that values of f_0 are scaled **down** for values of *s* such that $s < s_0$ and scaled **up** proportionally to p when $s \geq s_0$. More formally, the distribution is updated according to the following equation:

$$f_{t+1} = \begin{cases} \frac{p}{F_t(s)} f_t(s), & \text{for } s \geq s_t \\ \frac{q}{F_t(s)} f_t(s), & \text{for } s < s_t \end{cases}$$

…where F_t stands for the cumulative distribution function of f_t, and $q = 1 - p$.

If, on the other hand, the user annotates this tweet as indeed implying the claim, we can infer that the optimal threshold *S** we are looking for will be **lower** than s_0 with a probability *p*. We therefore update the probability distribution so that values of f_0 are scaled **up** for values of *s* such that $s < s_0$ and scaled **down** when $s \geq s_0$. More formally, the distribution is updated according to the following equation:

$$f_{t+1} = \begin{cases} \frac{q}{F_t(s)} f_t(s), & \text{for } s \geq s_t \\ \frac{p}{F_t(s)} f_t(s), & \text{for } s < s_t \end{cases}$$

These steps are repeated until the probability distribution is considered sufficiently concentrated around a threshold by the user of the system, or until no datapoint can be found close enough to a given threshold m_t. The median of this final probability distribution then becomes the threshold for the given claim.

Our own implementation of the threshold-tuning algorithm uses a value of *p* equal to 0.7. Moreover, we calculate the similarity (as the ratio of common tokens) between every document newly selected for annotation and documents that have already been annotated. If the new document is too similar to other documents, another one is selected. Moreover, our implementation allows the researcher to replace the currently selected document with a different tweet with approximately the same score if they wish (e.g. if the document is too ambiguous). For more information on the PBA, please refer to Author 1 & Author 4 (2024).

E. Threshold-tuning

Annotation traces

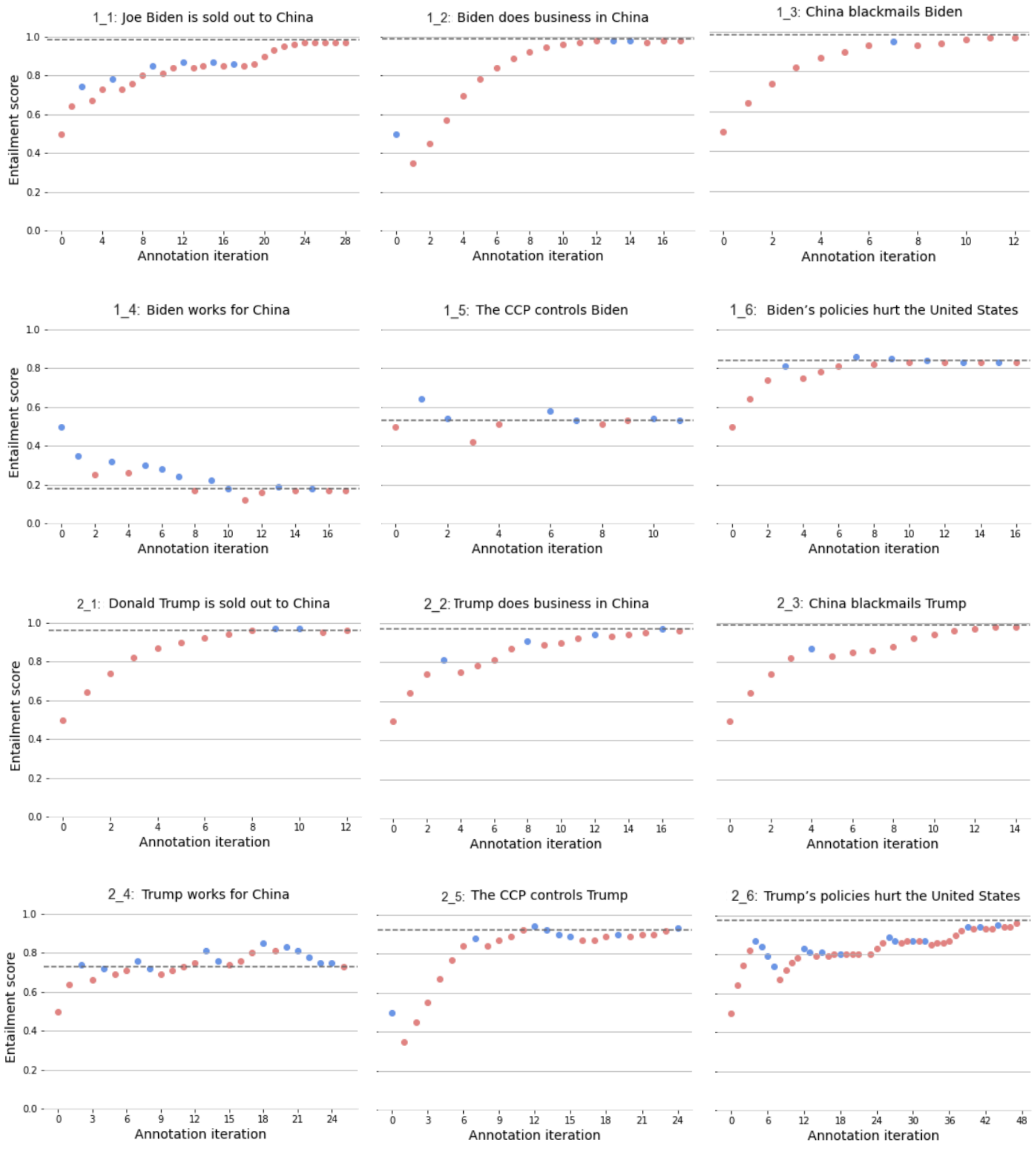

Final probability distribution for location of threshold

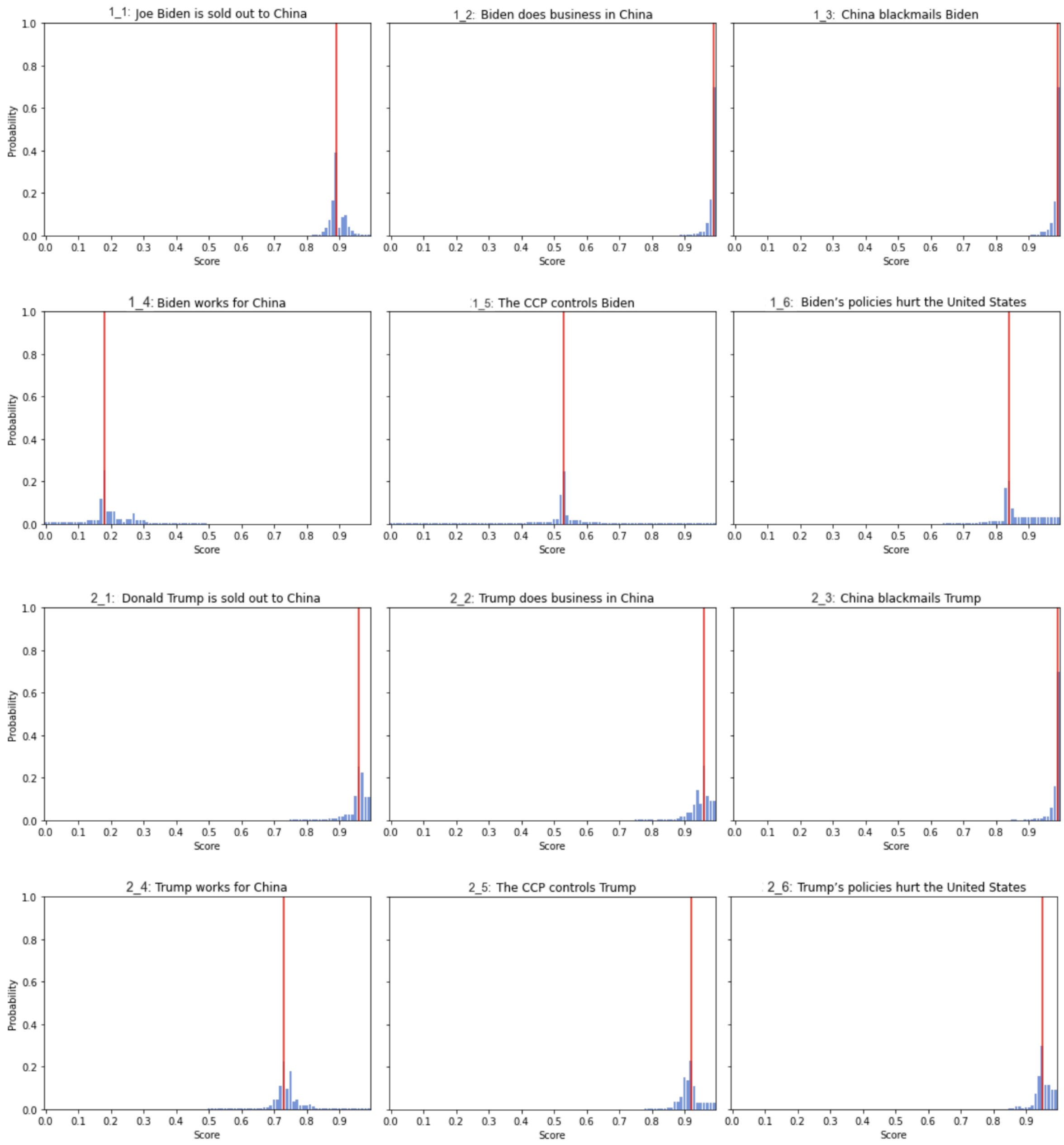

F. Results from the initial iteration of our methodology

The following diagrams visualize the frequency of each claim from our initial taxonomy of claims (see Appendix B for the list) between February 2020 and July 2021 in a random sample of 10,000 tweets per month. It is worth noting that for this first iteration, we used a different method for tuning the threshold and estimating the performance of the model for each claim. Instead of using the Probabilistic Bisection Algorithm and the three heuristics presented in the main paper, we simply randomly sampled 10 tweets per claim and annotated them. The final threshold was then defined as that which maximized the accuracy for each claim. We estimated the performance of the model then simply by recording this accuracy, and by calculating the f1-score from this handful of annotations. Tweet/claim pairs annotated as “ambiguous” were ignored. These thresholds and metrics are summarized in the table that follows the diagrams.

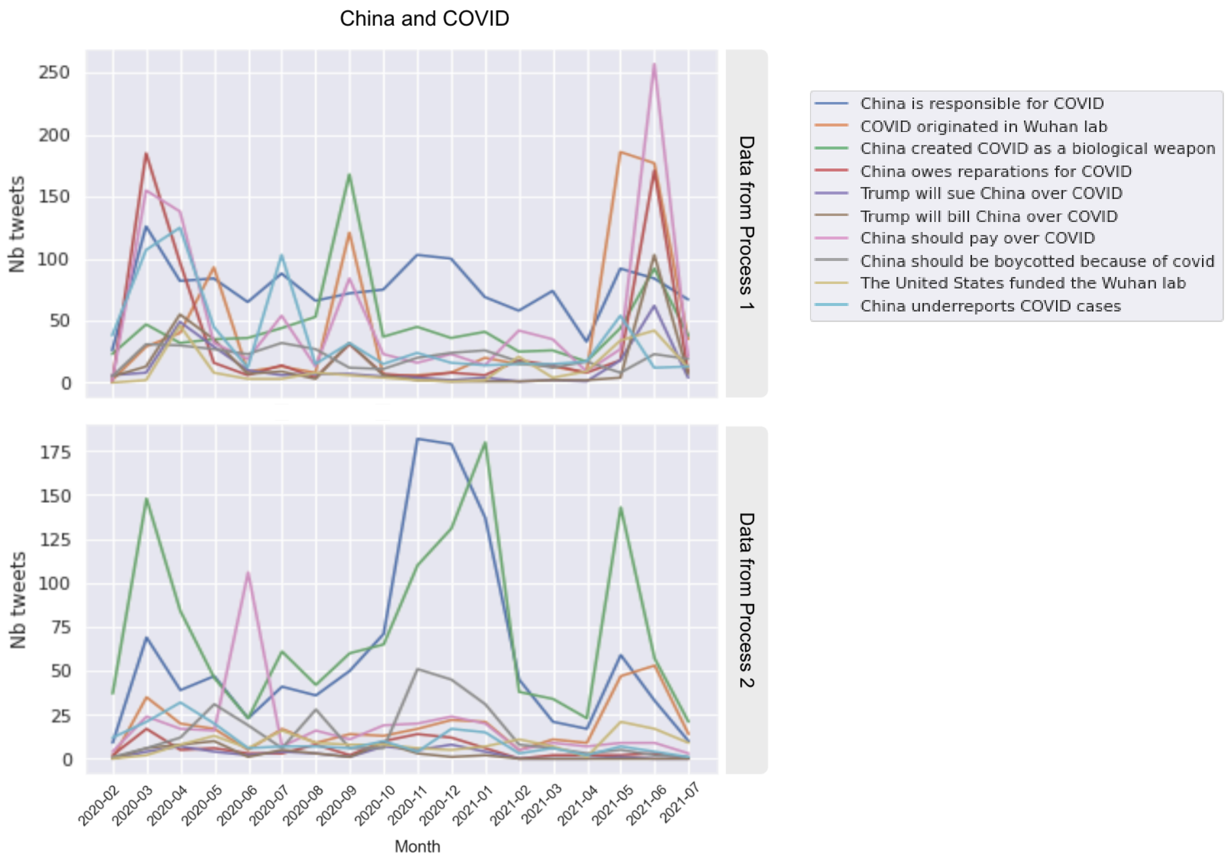

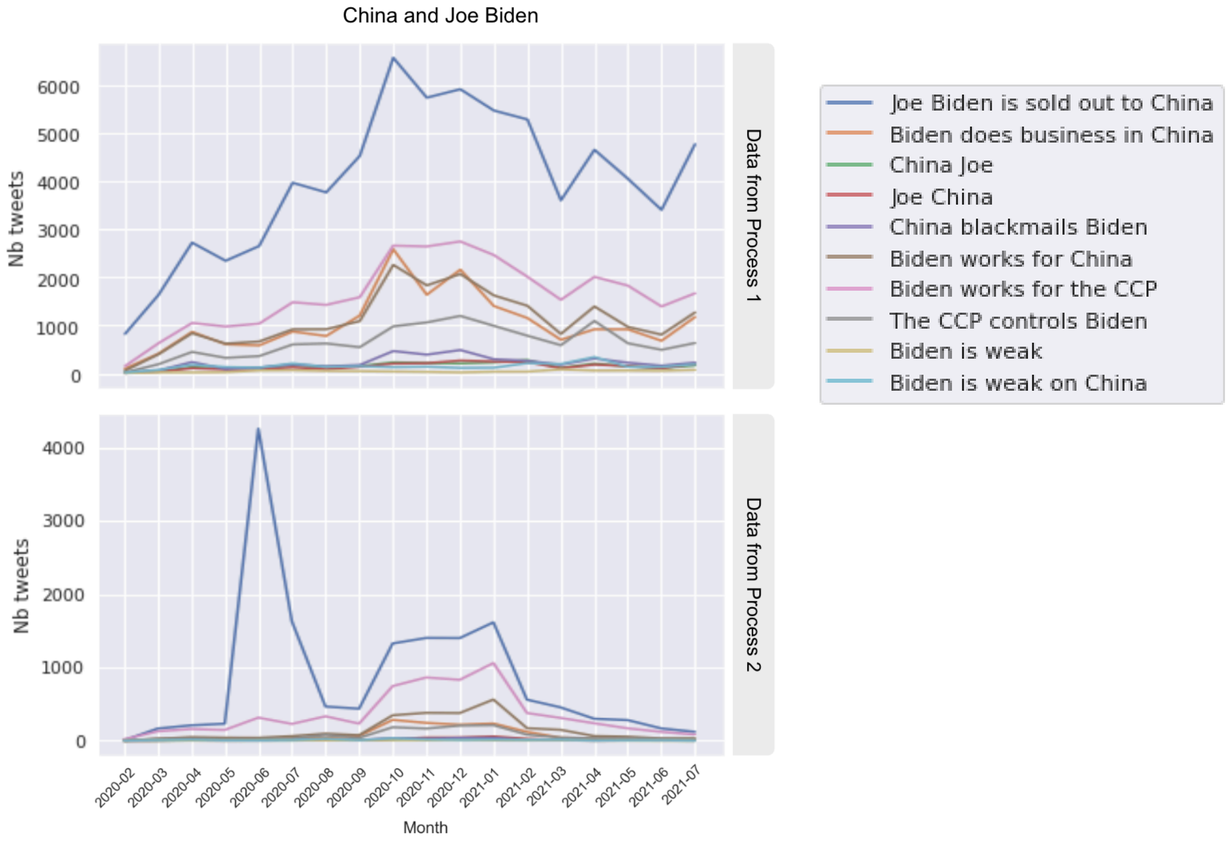
China and Joe Biden
Nb tweets
Data from Process 1
Data from Process 2
Month
Joe Biden is sold out to China
Biden does business in China
China Joe
Joe China
China blackmails Biden
Biden works for China
Biden works for the CCP
The CCP controls Biden
Biden is weak
Biden is weak on China

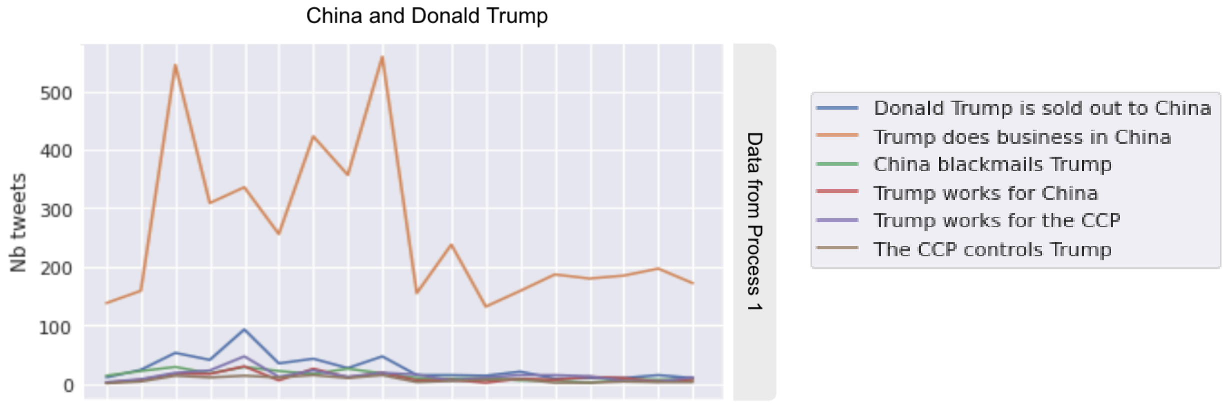
China and Donald Trump
Nb tweets
Data from Process 1
Donald Trump is sold out to China
Trump does business in China
China blackmails Trump
Trump works for China
Trump works for the CCP
The CCP controls Trump

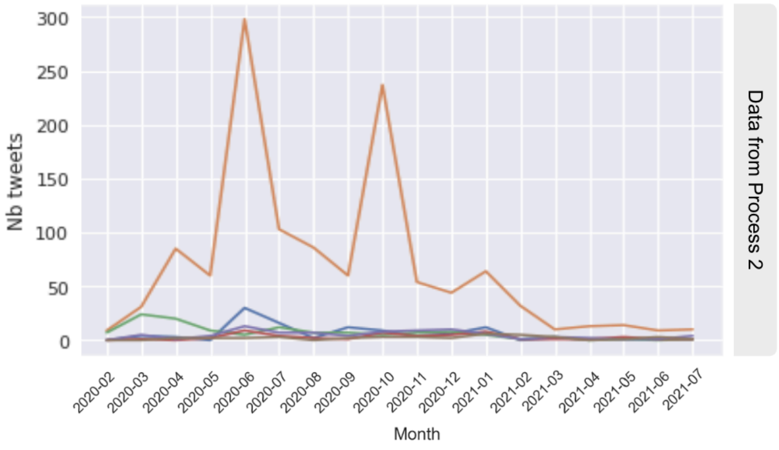

China helps Biden win

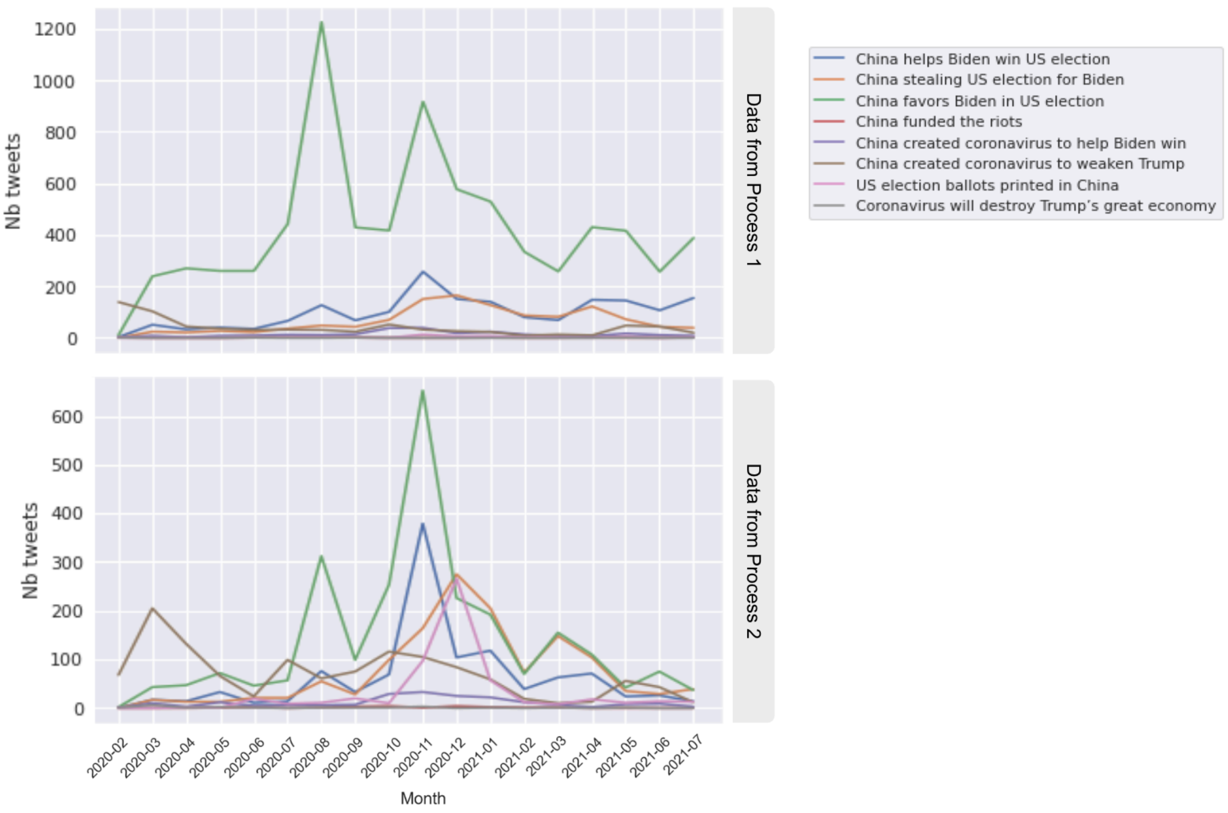

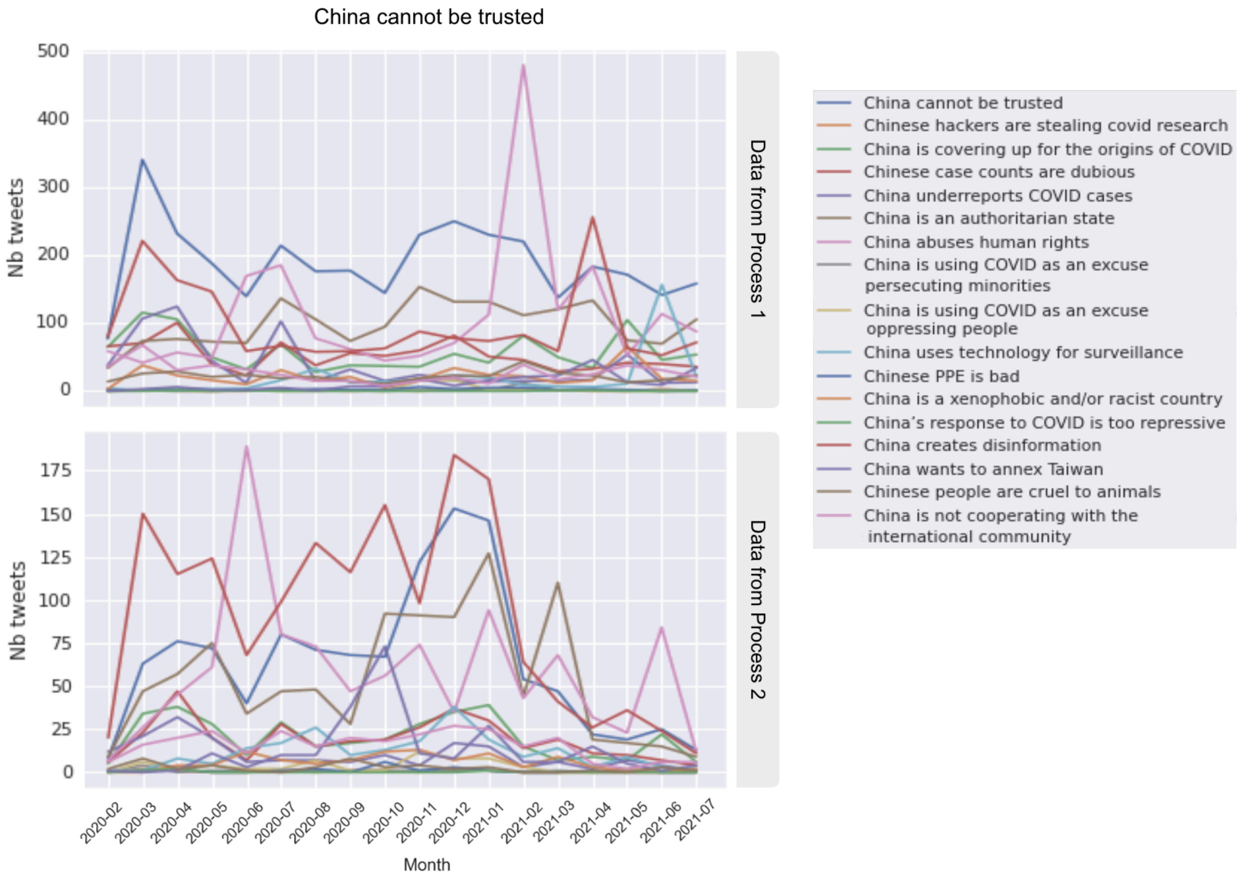
China cannot be trusted
Nb tweets
500
400
300
200
100
0
175
150
125
100
75
50
25
0
Data from Process 1
Data from Process 2
2020-02
2020-03
2020-04
2020-05
2020-06
2020-07
2020-08
2020-09
2020-10
2020-11
2020-12
2021-01
2021-02
2021-03
2021-04
2021-05
2021-06
2021-07
Month
China cannot be trusted
Chinese hackers are stealing covid research
China is covering up for the origins of COVID
Chinese case counts are dubious
China underreports COVID cases
China is an authoritarian state
China abuses human rights
China is using COVID as an excuse persecuting minorities
China is using COVID as an excuse oppressing people
China uses technology for surveillance
Chinese PPE is bad
China is a xenophobic and/or racist country
China's response to COVID is too repressive
China creates disinformation
China wants to annex Taiwan
Chinese people are cruel to animals
China is not cooperating with the international community

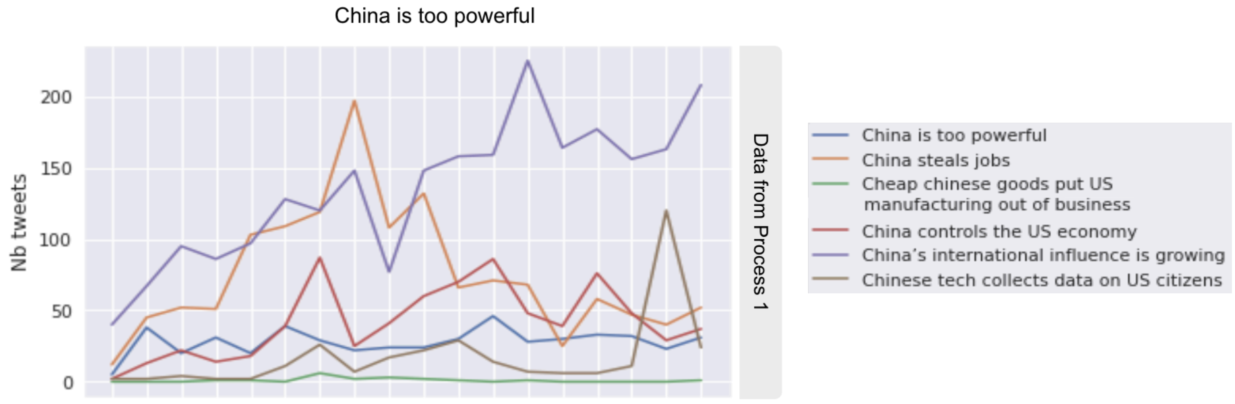
China is too powerful
Nb tweets
200
150
100
50
0
Data from Process 1
China is too powerful
China steals jobs
Cheap chinese goods put US manufacturing out of business
China controls the US economy
China's international influence is growing
Chinese tech collects data on US citizens

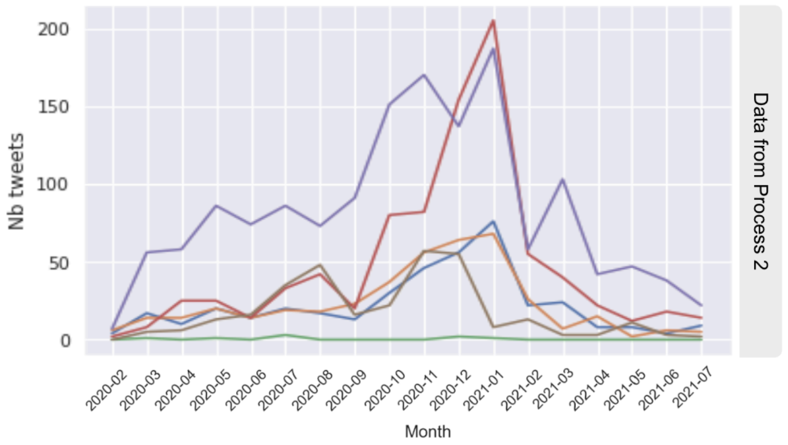

Xenophobia against China

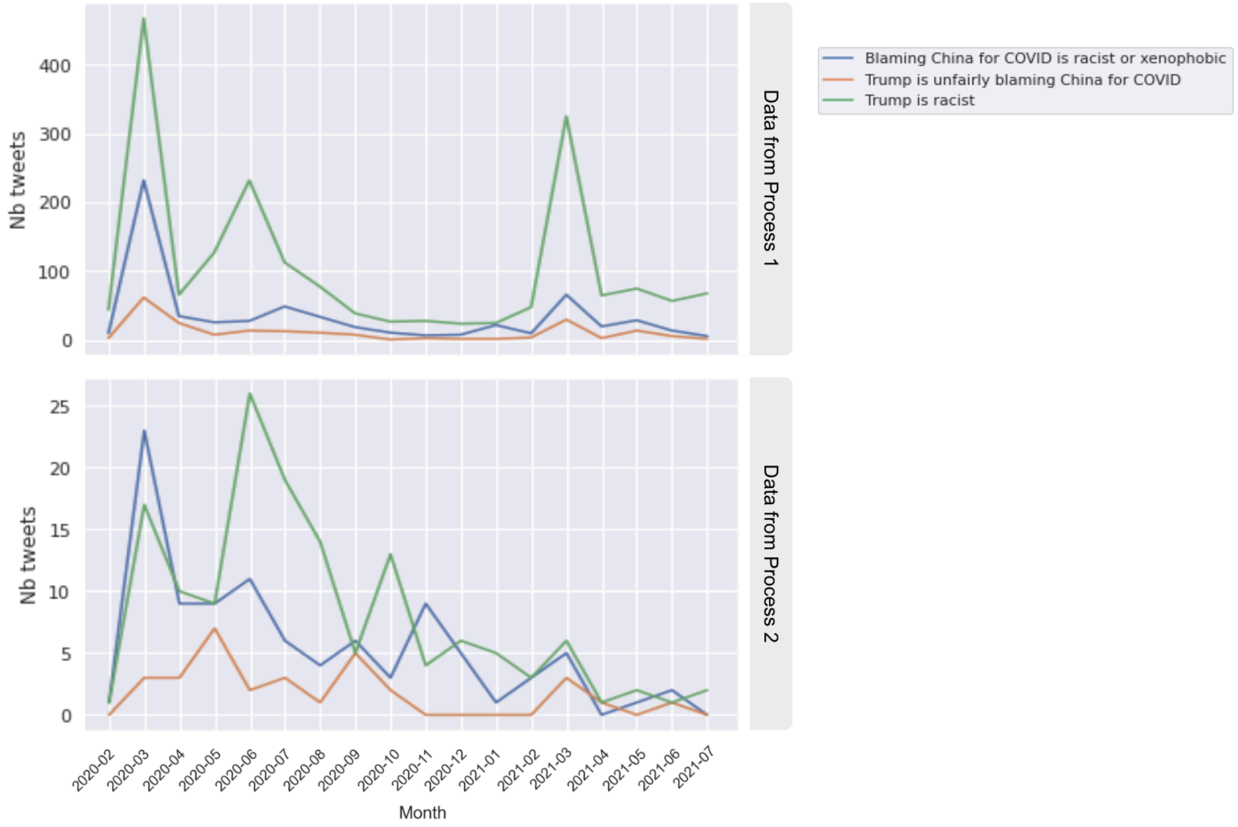

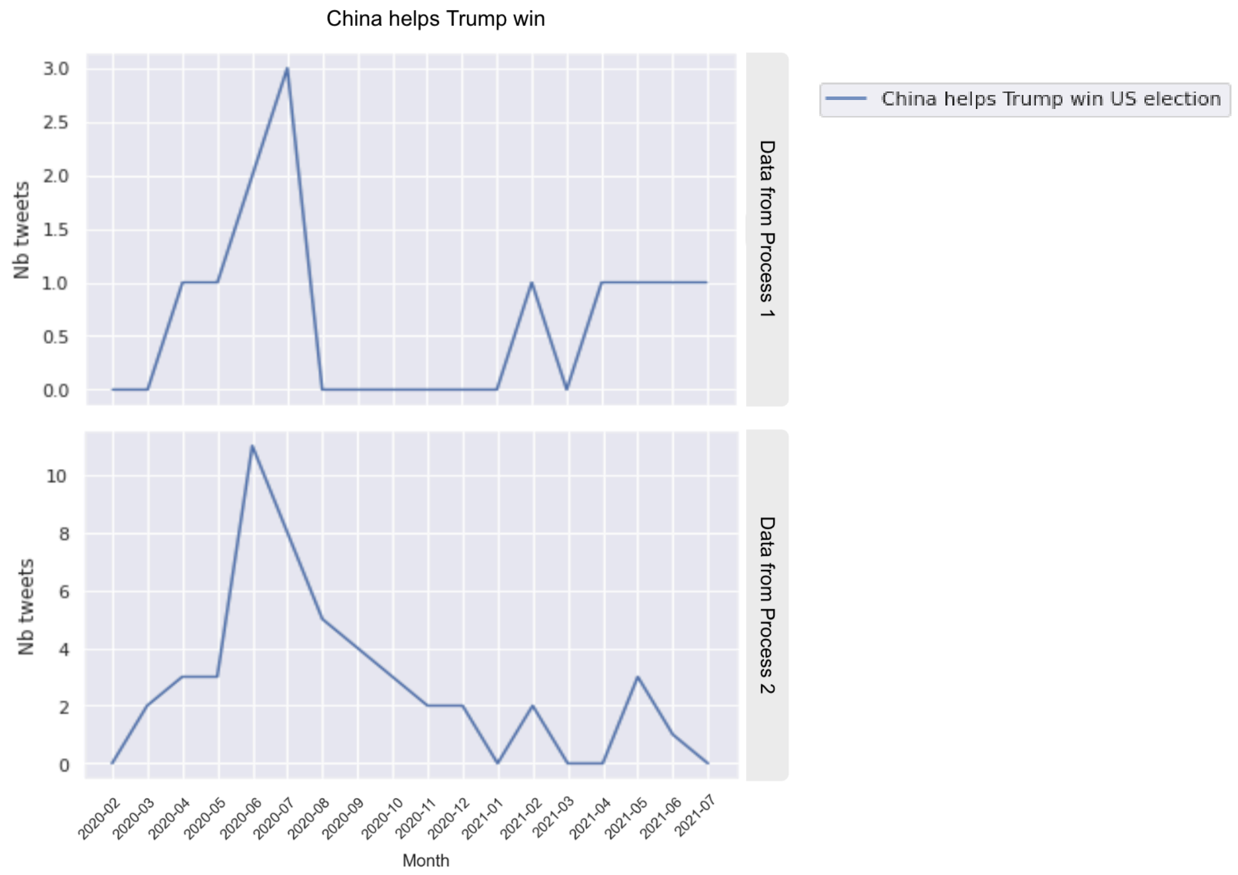
China helps Trump win
Nb tweets
Data from Process 1
Data from Process 2
China helps Trump win US election
Month
2020-02
2020-03
2020-04
2020-05
2020-06
2020-07
2020-08
2020-09
2020-10
2020-11
2020-12
2021-01
2021-02
2021-03
2021-04
2021-05
2021-06
2021-07

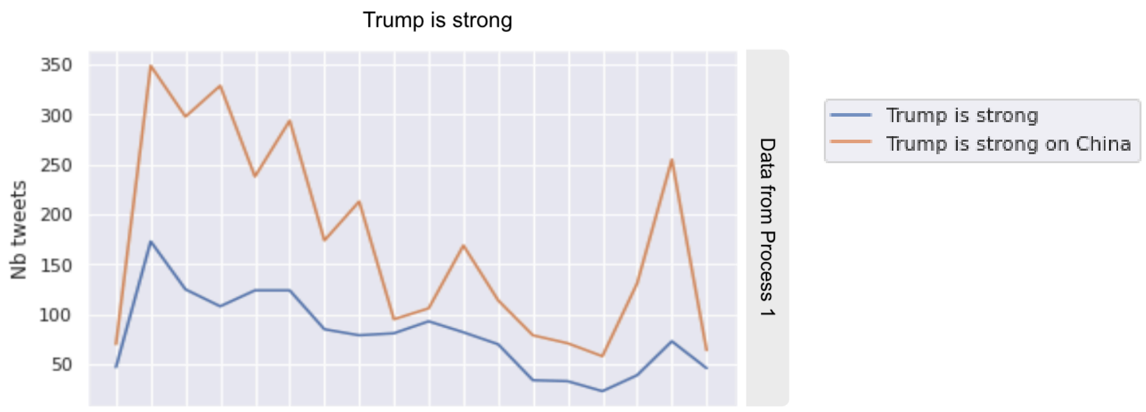
Trump is strong
Nb tweets
Data from Process 1
Trump is strong
Trump is strong on China

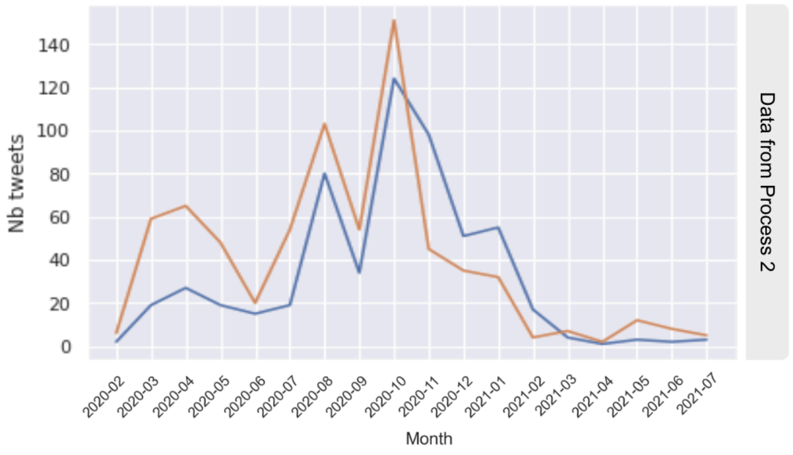

Claim	Threshold	Accuracy	F1-score
China is responsible for COVID	0.657	0.900	0.474
China is covering up for the origins of COVID	0.522	0.889	0.883
COVID originated in Wuhan lab	0.819	0.900	0.867
China is using COVID as an excuse to oppress people	0.686	0.750	0.429
China created coronavirus to help Biden win	0.758	0.900	0.867
China is not cooperating with the international community	0.714	0.800	0.444
China created COVID as a biological weapon	0.526	0.900	0.890
Joe Biden is sold out to China	0.682	0.714	0.417
Biden does business in China	0.873	0.556	0.550
China Joe or Joe China	0.420	0.700	0.600
China blackmails Biden	0.973	0.700	0.412
Biden works for China	0.622	0.667	0.585
Biden works for the CCP	0.418	0.857	0.462
The CCP controls Biden	0.571	1.000	1.000
Donald Trump is sold out to China	0.631	0.778	0.775

Trump does business in China	0.819	0.875	0.467
China blackmails Trump	0.696	0.778	0.438
Trump works for China	0.592	0.667	0.649
Trump works for the CCP	0.499	0.667	0.585
The CCP controls Trump	0.692	0.700	0.412
China helps Biden win US election	0.902	0.778	0.679
China stealing US election in favor of Biden	0.798	0.714	0.650
China favors Biden in US election	0.538	1.000	1.000
China funded the riots	0.714	0.900	0.804
China created coronavirus to help Biden win	0.758	0.900	0.867
China created coronavirus to weaken Trump	0.470	0.875	0.855
US election ballots printed in China	0.694	0.778	0.679
China cannot be trusted	0.698	0.750	0.429
Chinese hackers are stealing covid research	0.525	0.900	0.804
China is covering up for the origins of COVID	0.522	0.889	0.883
Chinese case counts are dubious	0.532	0.700	0.412
China underreports COVID cases	0.894	0.800	0.444
China is an authoritarian state	0.675	0.875	0.795
China abuses human rights	0.726	0.900	0.474
China is using COVID as an excuse for persecuting minorities	0.686	1.000	1.000
China uses technology for surveillance	0.772	0.800	0.444
Chinese PPE is bad	0.777	0.800	0.444
Chinese vaccines are ineffective	0.738	1.000	1.000
China is a xenophobic and/or racist country	0.897	0.778	0.438
China’s response to COVID is too repressive	0.576	1.000	1.000
China creates disinformation/misinformation	0.908	0.900	0.804

China wants to annex Taiwan	0.624	1.000	1.000
Chinese people are cruel to animals	0.825	0.889	0.471
China is too powerful	0.516	0.714	0.708
China is powerful	0.840	1.000	1.000
China steals jobs	0.670	0.800	0.444
Cheap chinese goods put US manufacturing out of business	0.629	1.000	1.000
China controls the US economy	0.448	0.556	0.357
China's international influence is growing	0.792	0.900	0.474
Chinese tech collects data on US citizens	0.805	0.800	0.444
China should be boycotted because of covid	0.857	0.889	0.862
Trump is unfairly blaming China for COVID	0.670	0.750	0.429
Trump is racist	0.546	0.800	0.792
China helps Trump win US election	0.897	0.750	0.429
US intelligence say that China is interfering in US elections	0.760	0.833	0.778
US intelligence say that China is not interfering in US elections	0.764	0.667	0.400
Biden is weak	0.908	1.000	1.000
Biden is weak on China	0.705	0.889	0.862
Trump is strong	0.850	1.000	1.000
Trump is strong on China	0.958	0.778	0.438
The United States funded the Wuhan lab	0.587	0.600	0.524
China manages the pandemic well	0.869	1.000	1.000
Blaming China for COVID is racist or xenophobic	0.873	0.800	0.762
Coronavirus will destroy Trump's great economy	0.442	0.889	0.862

G. Temporal frequency graphs

1. Without community labels

The following temporal frequency graph include the 68% confidence interval (see shaded area). Claims which did not pass the three quality heursistics presented in the main paper are not shown here.

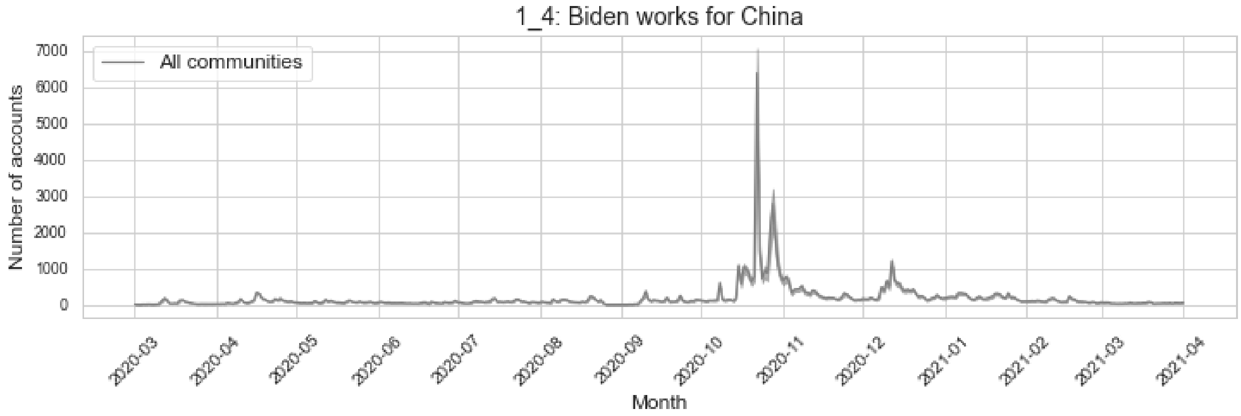

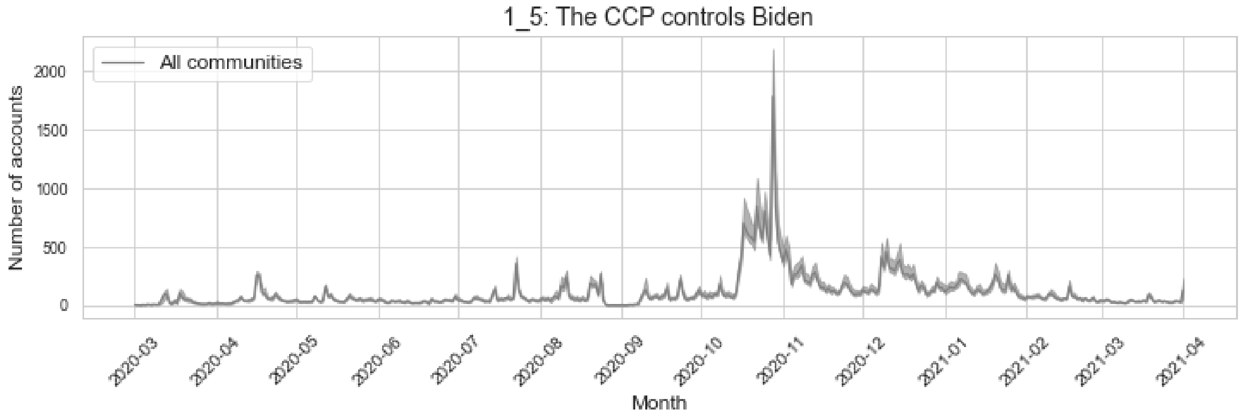

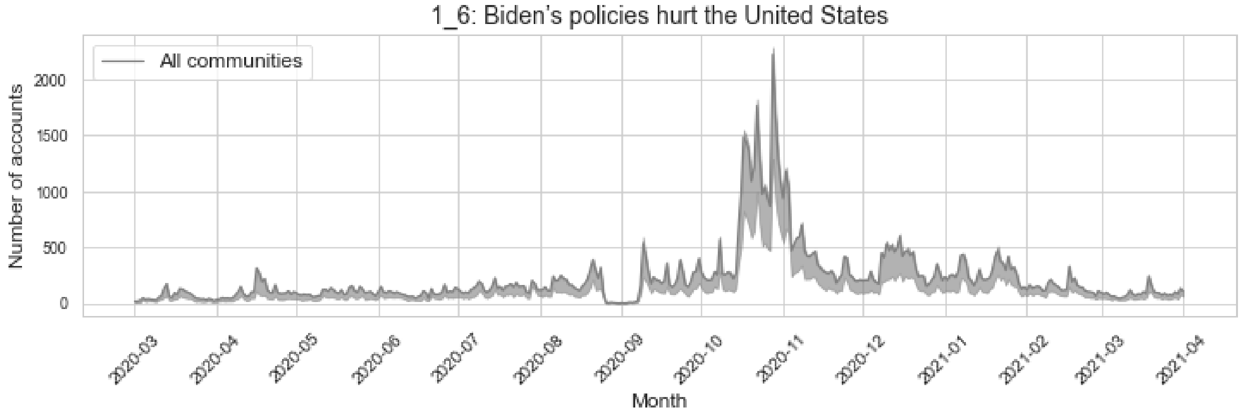
1_6: Biden's policies hurt the United States
All communities
Number of accounts
0
500
1000
1500
2000
2020-03
2020-04
2020-05
2020-06
2020-07
2020-08
2020-09
2020-10
2020-11
2020-12
2021-01
2021-02
2021-03
2021-04
Month

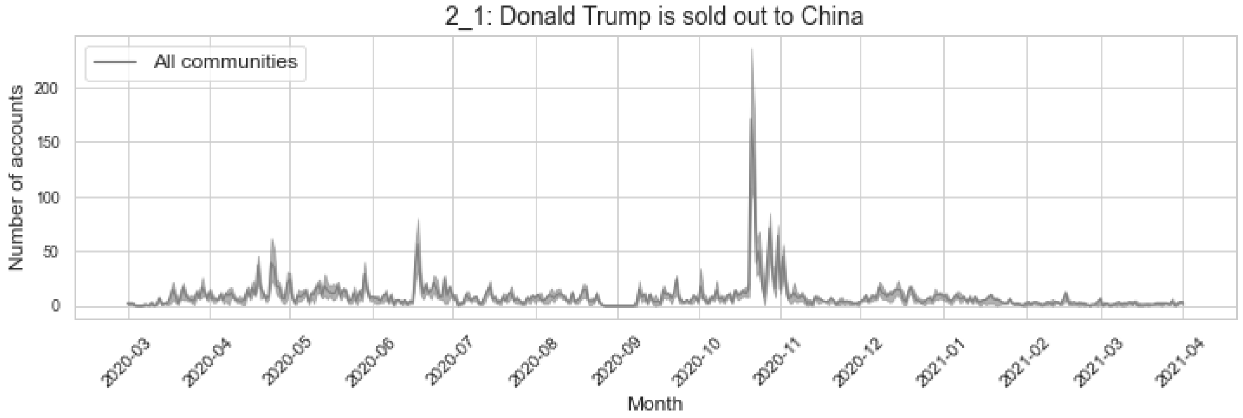
2_1: Donald Trump is sold out to China
All communities
Number of accounts
0
50
100
150
200
2020-03
2020-04
2020-05
2020-06
2020-07
2020-08
2020-09
2020-10
2020-11
2020-12
2021-01
2021-02
2021-03
2021-04
Month

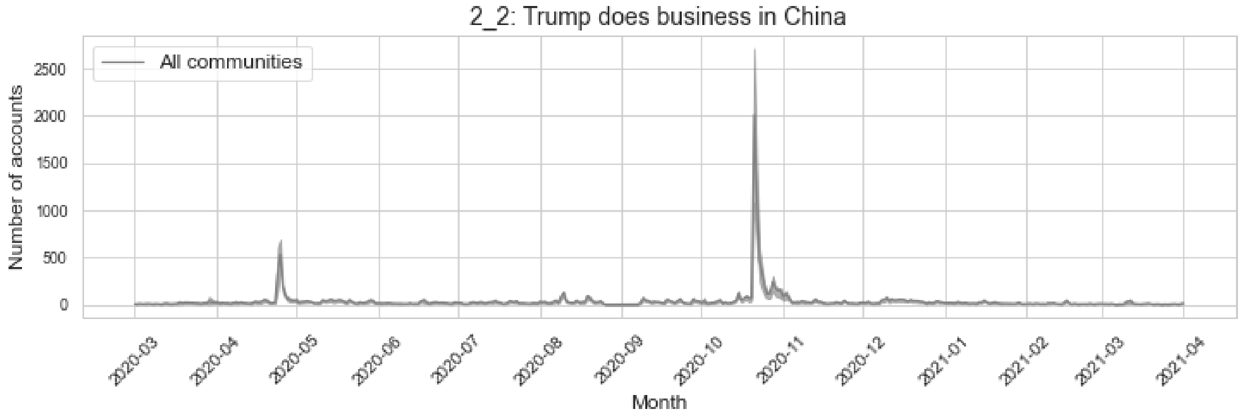
2_2: Trump does business in China
All communities
Number of accounts
0
500
1000
1500
2000
2500
2020-03
2020-04
2020-05
2020-06
2020-07
2020-08
2020-09
2020-10
2020-11
2020-12
2021-01
2021-02
2021-03
2021-04
Month

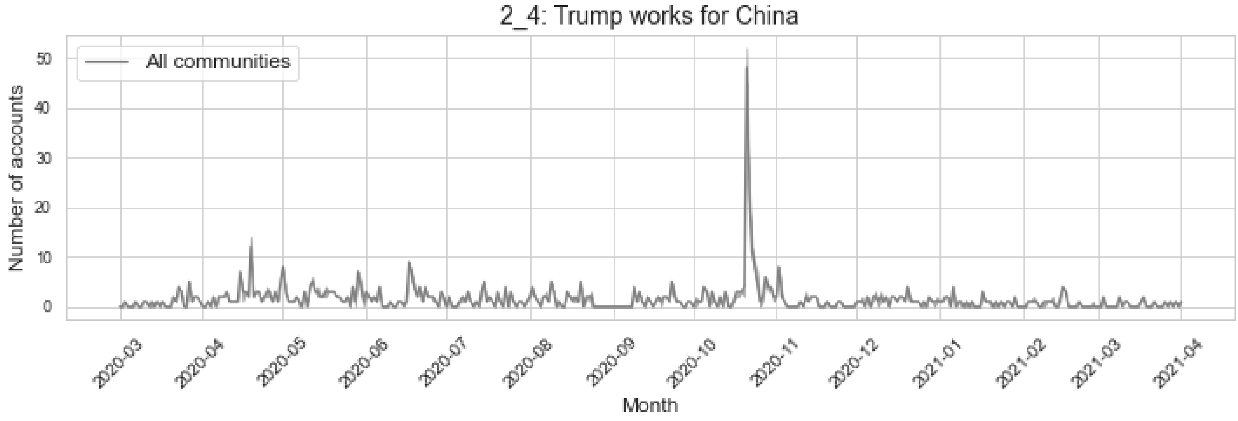
2_4: Trump works for China
All communities
Number of accounts
50
40
30
20
10
0
2020-03
2020-04
2020-05
2020-06
2020-07
2020-08
2020-09
2020-10
2020-11
2020-12
2021-01
2021-02
2021-03
2021-04
Month

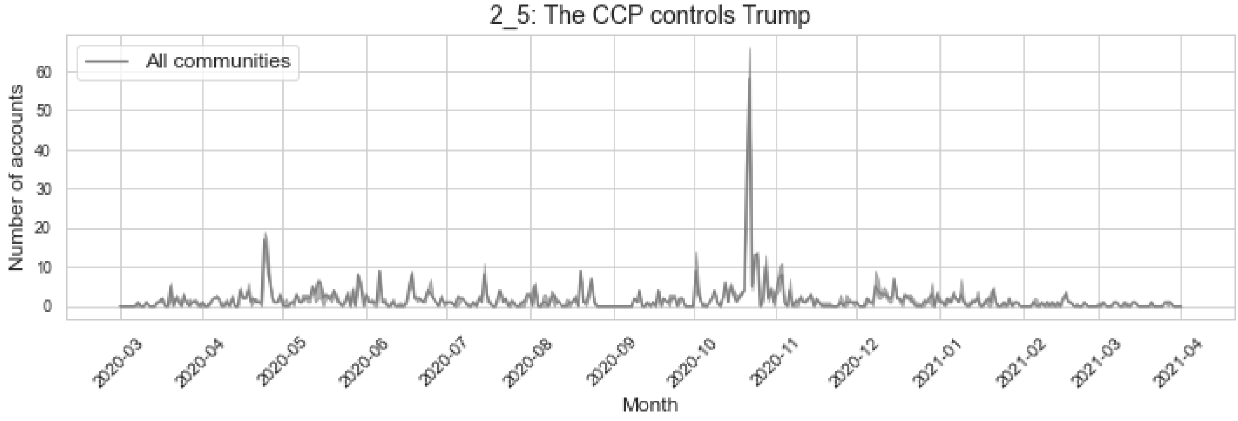
2_5: The CCP controls Trump
All communities
Number of accounts
60
50
40
30
20
10
0
2020-03
2020-04
2020-05
2020-06
2020-07
2020-08
2020-09
2020-10
2020-11
2020-12
2021-01
2021-02
2021-03
2021-04
Month

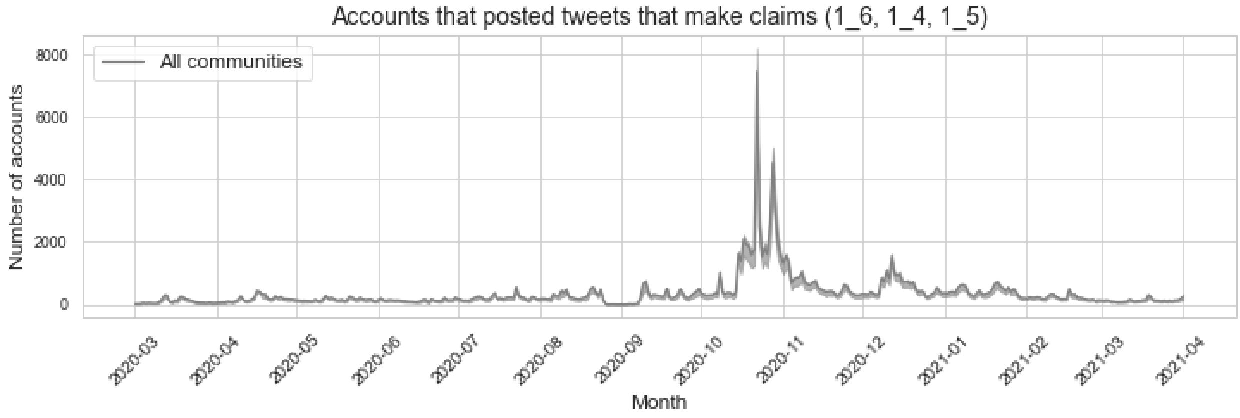
Accounts that posted tweets that make claims (1_6, 1_4, 1_5)
All communities
Number of accounts
8000
6000
4000
2000
0
2020-03
2020-04
2020-05
2020-06
2020-07
2020-08
2020-09
2020-10
2020-11
2020-12
2021-01
2021-02
2021-03
2021-04
Month

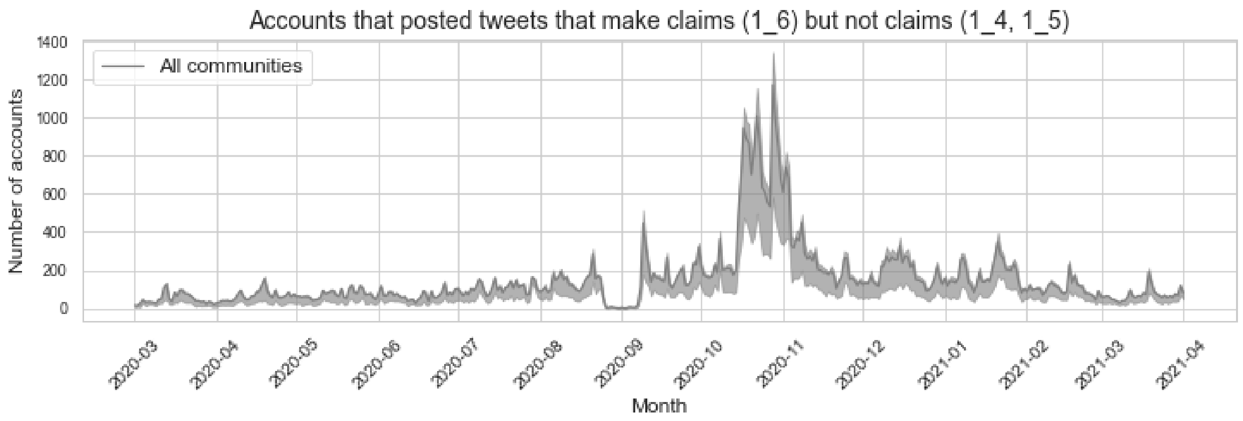

H. Annotation task

1. Annotation instructions

You will be shown a tweet about China and the 2020 US Presidential Election as well as a claim about the relationship between the two. Your task is to read the tweet and decide if the tweet contains/implies the specific claim, selecting "YES" or "NO" accordingly.

Please note that your task is to only indicate the presence of the claim in the tweet and not to evaluate the validity or accuracy of the claim or of the tweet. Please also note that the task should be done in a neutral way, not taking into account personal opinions or feelings about the topic of the tweets.

Examples

Positive Example

- **Tweet**: "China is definitely trying to interfere with the 2020 US Presidential Election. They want Trump out of office."
- **Claim**: China is trying to influence the outcome of the 2020 US Presidential Election.
- **Annotation**: YES (i.e. the tweet contains/implies the specific claim)

Negative Example

- **Tweet**: "China's economy is struggling, but they are still a major player in international politics."
- **Claim**: China is trying to influence the outcome of the 2020 US Presidential Election.

- **Annotation**: NO (i.e. the tweet does not contain/imply the specific claim)

Important notes

- The tweets have been semi-anonymised so that Twitter handles (i.e. usernames) have been replaced by the token "[HANDLE]" and URLs have been replaced by the token "[URL]".
- For the purposes of this work, please consider China, the Chinese Communist Party, the Chinese government, or the Chinese state to be the same thing.
- References to Joe Biden's family (particularly Hunter Biden) or to Donald Trump's family also imply Biden himself or Trump himself.
- Past vs. present vs. future tense does not matter. You should indicate whether the claim is included in the tweet, whether or not it is discussed with regard to the past, present, or future.
- The tweet contains the claim if the tweet includes any reference to the claim, even if the claim is not the main point of the tweet. For example:

Tweet	Annotation
**Tweet**: "HEY FAKE JOE BIDEN.....More LIES ? China Joe Melon Brain LIES more than Pinocchio Schiff. How much did Hunter and THE BIG GUY pay in takes on that $ 85 BILLION DOLLAR ARMS SALE TO THE TALIBAN ? Kill anyone more Innocent Families or US Personel in AFGHANISTAN TODAY ? "<br><br>**Claim**: Biden Works for China	YES<br><br>**Explanation**: This tweet implies the claim "Biden Works for China", via the mention of "China Joe", even though this claim is not the main point of the tweet.

- Be sure to pay close attention to whether the claim and the tweet are both about the same person (Biden or Trump). Many tweets reference both Trump and Biden but include the claim only about one.
- Look carefully for sarcasm when evaluating tweets; sometimes sarcasm changes the meaning of the tweet from its literal meaning. For example:

Tweet	Annotation
**Tweet**: "Yes Donald trump is a Chinese agent just like you claimed he was a Russian agent, a Saudi agent, an Iranian agent and a Jewish agent"<br><br>**Claim**: Trump works for China	NO<br><br>**Explanation**: This tweet does NOT actually contain the claim "Trump works for China" because the author is using sarcasm.

Per claim description

Claims	Description
**2_0 / 3_0**: [Biden/Trump] is sold out to China	Biden or Trump is willing to do what China wants; accepted money or other bribes from China in exchange for favors or special treatment, especially contradictory to US interests
**2_1 / 3_1**: [Biden/Trump] does business in China	Biden or Trump has business interests in China that are influencing his decisions; no direct relationship with the Chinese government, but business interests influence decisions in his personal interest rather than the US national interest
**2_2 / 3_2**: China blackmails [Biden/Trump]	China has compromising information about Trump or Biden and is using the threat of disclosure to influence their decisions; China has secret and damaging information about improper conduct that it could reveal and therefore has leverage or could exert pressure; China is able to personally coerce Trump or Biden by exerting legal pressure; does not include bribes
**2_3 / 3_3**: [Biden/Trump] works for China	Implies an employment relationship with the Chinese government; being paid by China and therefore loyal to China rather than the US; does not include works IN China
**2_5 / 3_5**: The CCP controls [Biden/Trump]	Biden or Trump is unable to resist China's pressure; weak and vulnerable to coercion for any reason; does China's bidding, China outsmarts Biden or Trump; Trump or Biden is manipulated against their will; China is able to direct Trump or Biden to take specific actions
**2_10 / 3_10**: [Biden/Trump]'s policies hurt the United States	Biden or Trump actions and/or decisions are detrimental to the US, whether or not this is intentional or the product of dishonest behaviour

Note: these claims are not intended to be mutually exclusive, and each claim should be annotated in isolation: i.e. it should not matter whether or not another claim than the one you are annotating fits the tweet better.

2. Training examples

1_1 / 2_1: [Biden/Trump] is sold out to China

Demo tweet	Contains claim?
🤔... Corroborated messages, which appear to outline a payout to former Vice President Joe Biden as part of a deal with a Chinese energy firm.<br><br>#Breaking: Source on alleged #HunterBiden email chain verifies message about #ChineseInvestmentFirm [URL] #FoxNews	Yes
@JoeBiden Joe Biden doesn't deserve to live in America, much less be our president. Move to China!	No
📢Trump had a secret office in China during his 1st run for president, & is partners with a government-controlled company🤔<br>📢Trump maintains bank accounts in China—that don't appear on his financial disclosures—because they're held under corporate names [URL]	Yes

Exercise tweet	Contains claim?
@realDonaldTrump Donald Trump continues to call covid19 as China virus, because he wants us to believe US has so many cases is totally due to China who detected the virus but not his incompetence. He takes no responsibility for it.	No
Trump should only refer to Biden during the debate as "the Big guy" as Biden is referred to in the emails showing the international money pay offs from China and Ukraine going to Hunter "to hold for the big guy" aka Joe Biden. That would be so great!	Yes
@realDonaldTrump This is, of course, nonsense.<br><br>Remember that Donald Trump and Ivanka Trump had stuff made in China, and said it was just "good business". Same with using bootleg foreign steel.<br><br>So where was "America First" then? When it is HIS money, he sells out America like the Judas he is.	No

1_2 / 2_2: [Biden/Trump] does business in China

Demo tweet	Contains claim?
[HANDLE] Oh sounds like donald trumps merchandise made in China and Bangladesh! David Letterman Exposes Donald Trump [URL] via [HANDLE]	Yes

Devastating Information On Hunter And Joe Biden Come From Top Chinese Official Defected to the US [URL]	No
[HANDLE] Joe Biden didn’t take money DIRECTLY from China. It was laundered to him via his son, brother, & others.<br>Also, the pallets of money; especially the 1.7 BILLION IN CASH was probably then “smurfed” to Obama & Biden’s bank accounts.	No

**Exercise tweet**	**Contains claim?**
[HANDLE] [HANDLE] [HANDLE] Selling US state dept. influence to the Chinese is bad, is corruption at the highest levels of government, and if Joe Biden did it, he needs to burn.<br><br>If Kushner did anything corrupt, he and everyone involved, including Trump himself, needs to burn.<br><br>Burn. Them. All.	No
Joe Biden should not be faulted for Hunter's poor decisions BUT Hunter invested in Megvii, a firm providing CCTV cameras for China's Skynet program - a state wide surveillance project. There are reports that both Bidens were to make a profit off this investment.	Yes
[HANDLE] [HANDLE] [HANDLE] [HANDLE] [HANDLE] Alleged China-Fighter Donald Trump Has Secret Chinese Bank Account<br>[URL]	Yes

1_3 / 2_3: China blackmails [Biden/Trump]

**Demo tweet**	**Contains claim?**
Donald Trump personally owes 10s of millions to the Bank of China - so China ‘and’ Russia have the stable genius by the nads. [URL]	Yes
[HANDLE] joe biden works for china not our country	No
[HANDLE] Clean energy technology from parts made in China because Joe Biden is beholden to the Chinese Communist Party for the millions they've donated to him and Hunter.	No

**Exercise tweet**	**Contains claim?**
[HANDLE] [HANDLE] multiple videos on Hunter Biden's laptop showing him raping and torturing underage Chinese girls, and that the Chinese Communist Party is	Yes

using these videos as blackmail against Democrat presidential contender Joe Biden. [URL]	
[HANDLE] [HANDLE] Biden had the secret bank account in China not Trump, any deal Trump had in negotiation was ended by China perhaps because N Korea had a property deal, inquiry or due to Joe Biden, whatever came 1st.	No
Forbes: China Could Force Donald Trump And The Fed To Destroy The U.S. Banking System.<br>[URL]<br>via [HANDLE]	No
[HANDLE] The whole point is how compromised Joe Biden is.<br>China would own him!	No

1_4 / 2_4: [Biden/Trump] works for China

Demo tweet	Contains claim?
[HANDLE] So Joe Biden is now proven to be an unregistered foreign agent of China.	Yes
donald trump chinese	No
[HANDLE] Donald Trump cannot hols China accountable for anything.He and Ivanka,are China’s willing,collaborators.	Yes

Exercise tweet	Contains claim?
Jake Tapper Asked Joe Biden About The Wuhan Virus. He Replied With A Cough [URL]	No
[HANDLE] Donald Trump: *Secret bank account in China *Rcv'd $$$ after becoming prez from China<br><br>Ivanka Trump: *Countless patents from China *ELECTION MACHINES BUILT IN CHINA<br><br>And all your maga costumes and propaganda are made in China. Your hypocrisy is deafening [HANDLE] [URL]	Yes
Forbes: China Could Force Donald Trump And The Fed To Destroy The U.S. Banking System.<br>[URL]<br>via [HANDLE]	No
A vote for Joe Biden is a vote for China	No

[HANDLE] DONALD TRUMP DOES BUSINESS IN CHINA, AND WITH CHINA.	Yes
[HANDLE] Donald Trump has been pandering to China	No

1_5 / 2_5: The CCP controls [Biden/Trump]

Demo tweet	Contains claim?
[HANDLE] [HANDLE] Meet Joe Biden: China's Puppet<br>#Trump2020<br>[HANDLE]<br>. [URL]	Yes
Joe Biden must have paid off China	No
[HANDLE] 🇨🇳#MAGA!🇨🇳 Donald Trump’s Debt to China [URL] via [HANDLE] #ChinaOwnsTrump	Yes

Exercise tweet	Contains claim?
[HANDLE] No. Russia, North Korea, Iran, and China kept Donald Trump in their back pocket.	Yes
CHINA IN CHARGE: The Chinese Communists are Gloating: "Good Riddance Donald Trump" [URL] via [HANDLE]	No
[HANDLE] [HANDLE] He’s so scared. The Chinese are simply kicking his ass all around the table. What a weak dude, same goes for his boss Joe Biden 😄😂	Yes

1_6 / 2_6: The CCP controls [Biden/Trump]

Demo tweet	Contains claim?
Donald Trump is not working for Russia, he's working for China. This entire trade war is hurting American economic leadership both in the short term and in the long term as they turn away from imports and grow their domestic industry.	Yes
<HANDLE> Hunter Biden FBI Investigation into Money Laundering Schemes for Crooked Joe Biden in China and Ukraine... Americans have had Enough of this Liberal Dem Fake News BS!	No
Joe Biden’s 7 Cringeworthy Moments at Wisconsin Town Hall <URL> via <HANDLE> #8 was totally not mentioned, Forgot to introduce himself as Dictator	Yes

China-Joe, the real Trojan Horse for America.	

Exercise tweet	Contains claim?
<HANDLE> Joe Biden = the destruction of the USA. China will win without firing a single shot…	Yes
Joe Biden upset that Trump closed the border with China due to coronavirus <URL> via <HANDLE>	No
<HANDLE> Joe Biden China conspiracy, Joe Biden Russia conspiracy,and Joe Biden other country's conspiracy. Joe Biden got payed by each of them. Joe Biden sold out the United States to each of them . Being a traitor to the United States. Joe Biden did treason to the United States.	Yes

3. Annotation results

Annotation of stratified sample

The figures below visually represent the annotations provided by the three annotators for each claim. The y-axis corresponds to the annotator, while the x-axis represents the percentile to which the entailment score of each data point belongs. In other words, the data in the stratified sample is here shown as sorted by the entailment score. Finally, data points annotated as containing the claim are shown in blue, while those that are annotated as *not* entailing the claim are shown in red.

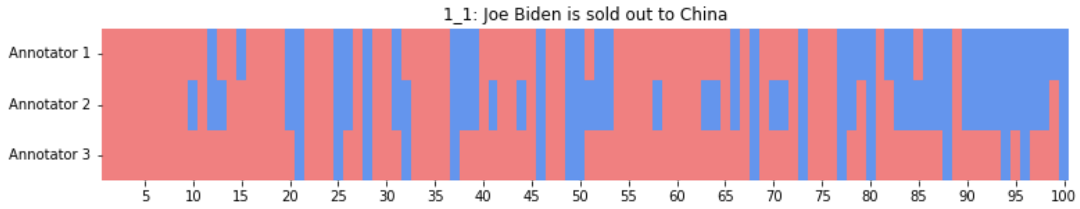

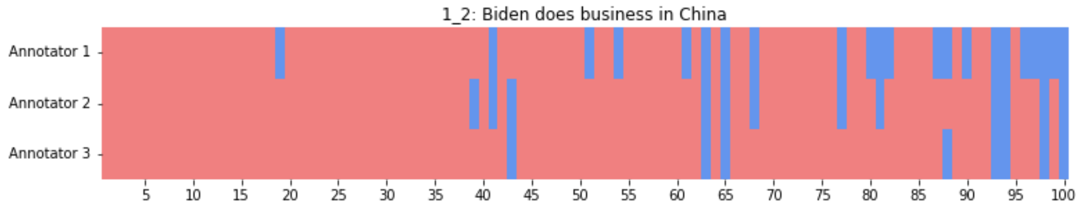

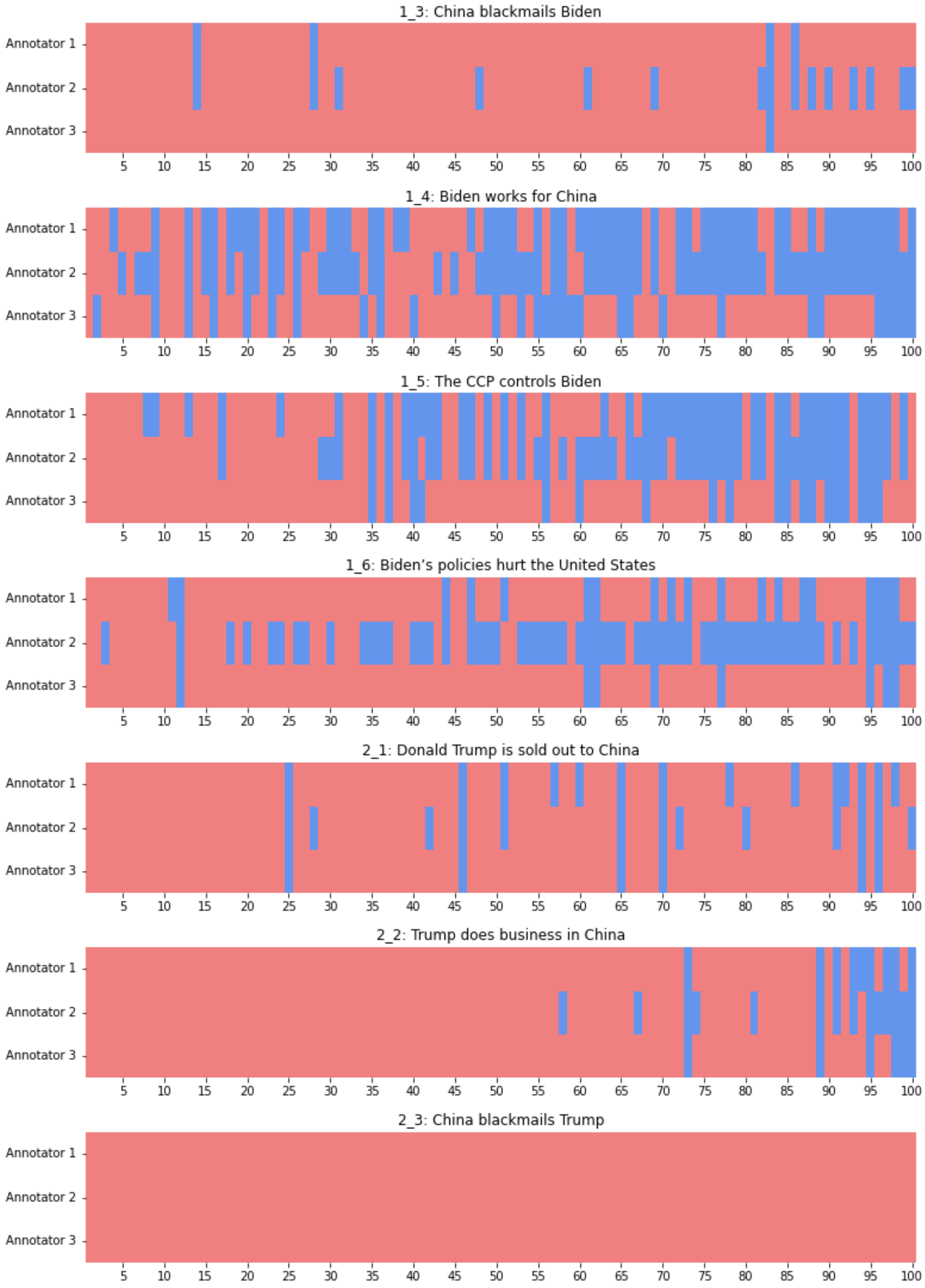
1_3: China blackmails Biden
1_4: Biden works for China
1_5: The CCP controls Biden
1_6: Biden's policies hurt the United States
2_1: Donald Trump is sold out to China
2_2: Trump does business in China
2_3: China blackmails Trump
Annotator 1
Annotator 2
Annotator 3
5 10 15 20 25 30 35 40 45 50 55 60 65 70 75 80 85 90 95 100

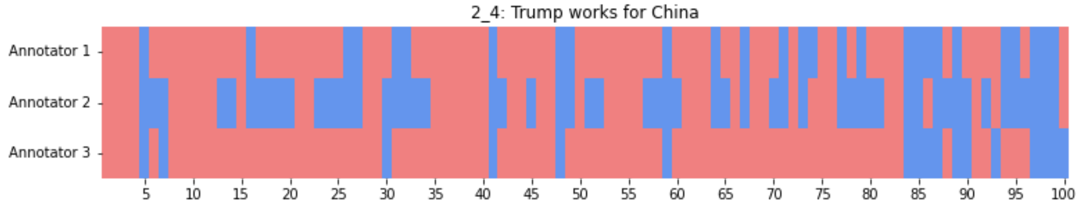

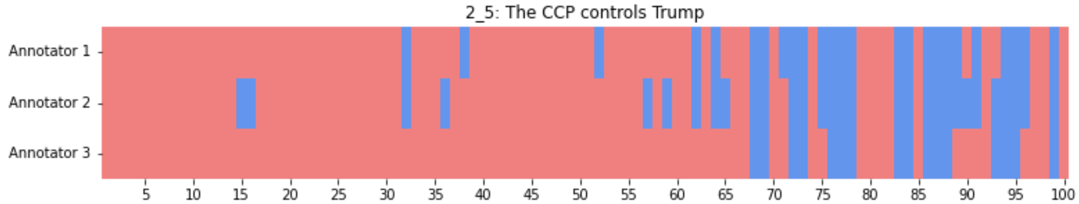

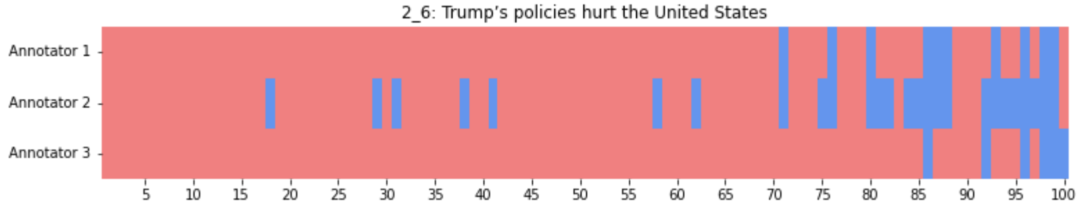

Annotations for threshold-tuning

Annotator 1

The figures below show the annotation trace returned by the threshold-tuning for Annotator 1.

1_1: Joe Biden is sold out to China 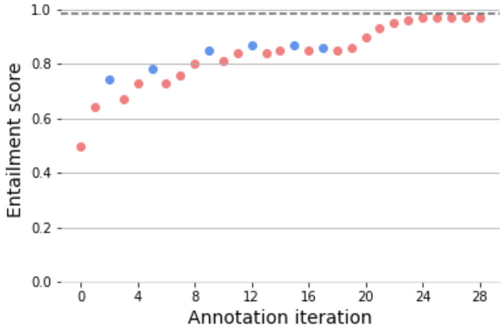	1_2: Biden does business in China 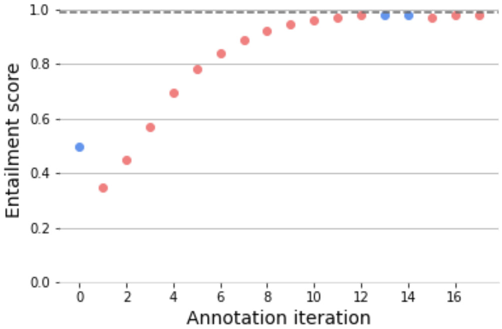
1_3: China blackmails Biden	1_4: Biden works for China

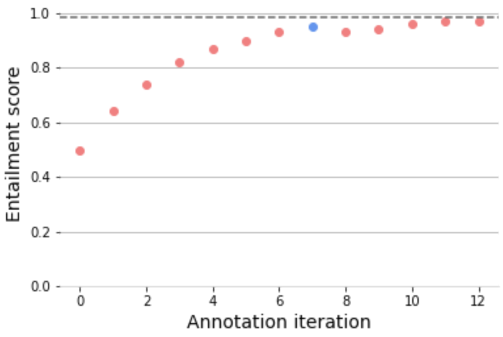

1_5: The CCP controls Biden

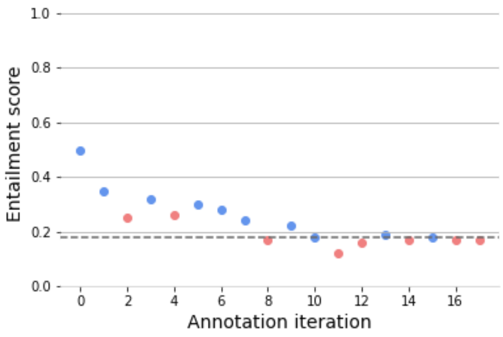

1_6: Biden's policies hurt the United States

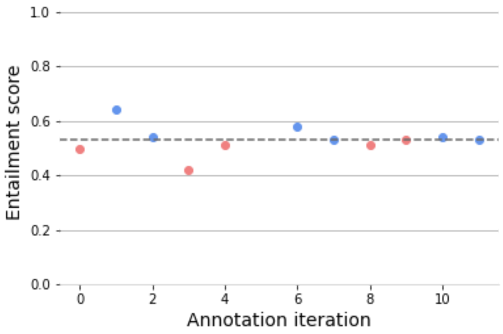

2_1: Donald Trump is sold out to China

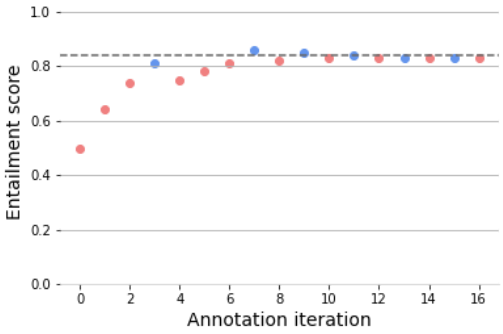

2_2:Trump does business in China

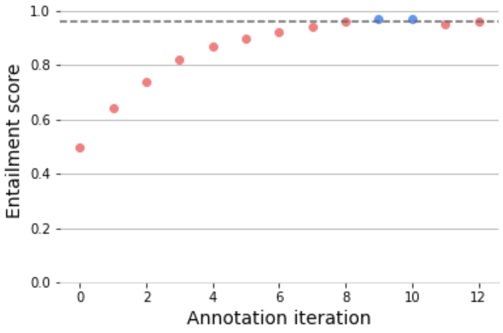

2_3: China blackmails Trump

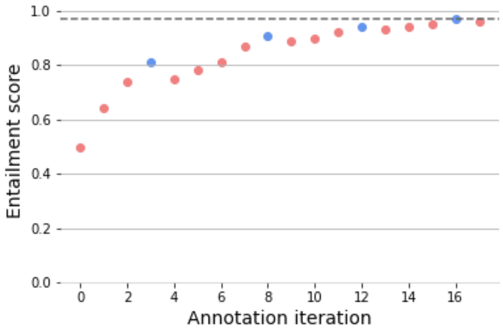

2_4: Trump works for China

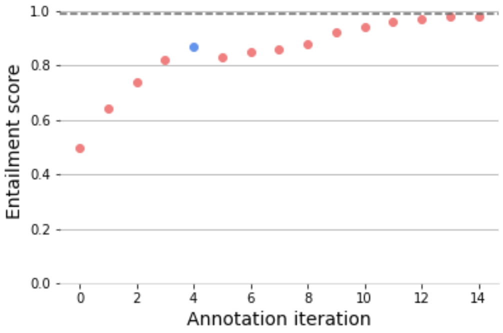

2_5: The CCP controls Trump

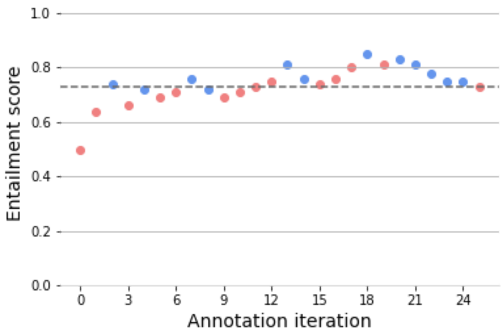

2_6: Trump's policies hurt the United States

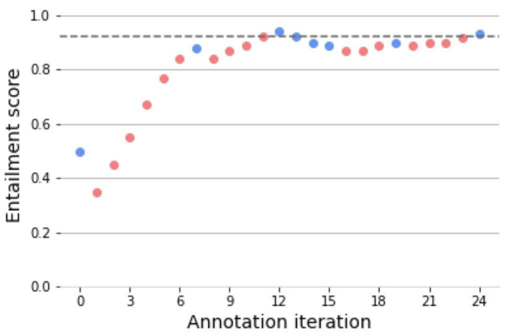	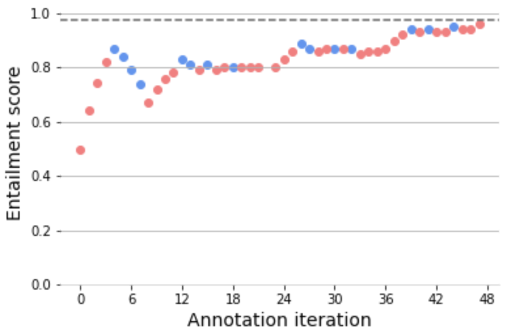

Annotator 2

The figures below show the annotation trace returned by the threshold-tuning for Annotator 2.

1_1: Joe Biden is sold out to China<br>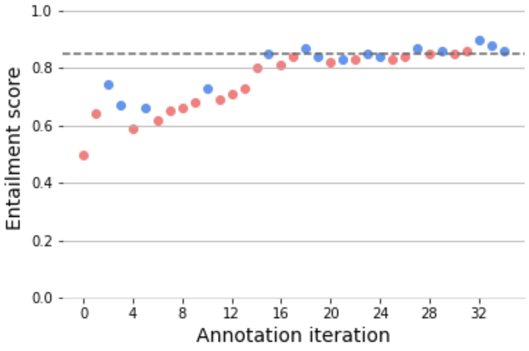	1_2: Biden does business in China<br>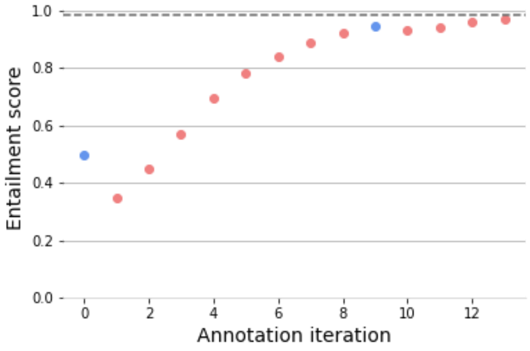
1_3: China blackmails Biden<br>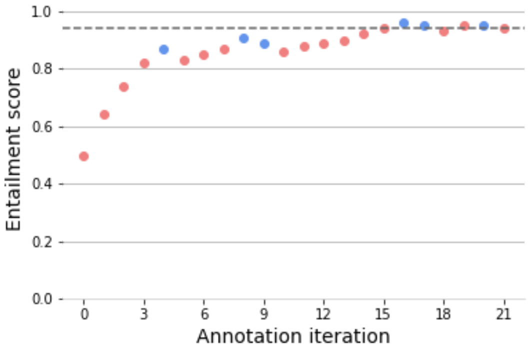	1_4: Biden works for China<br>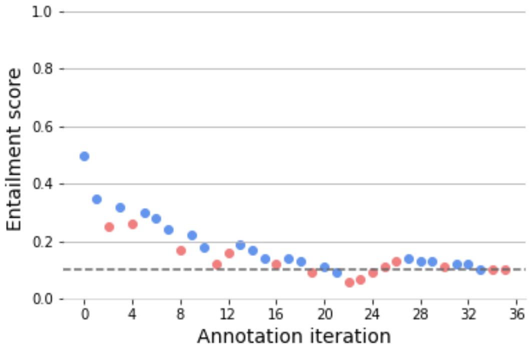
1_5: The CCP controls Biden	1_6: Biden's policies hurt the United States

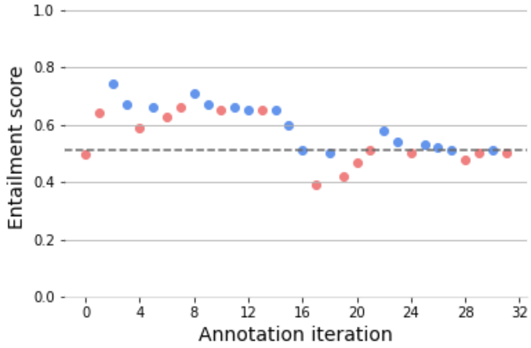

2_1: Donald Trump is sold out to China

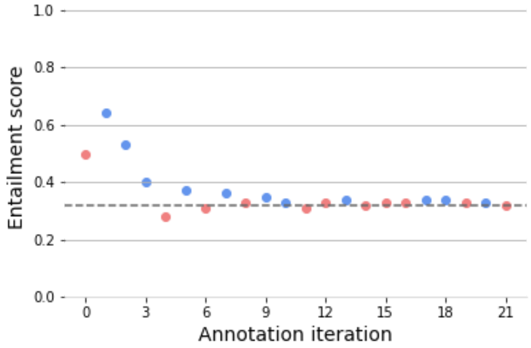

2_2:Trump does business in China

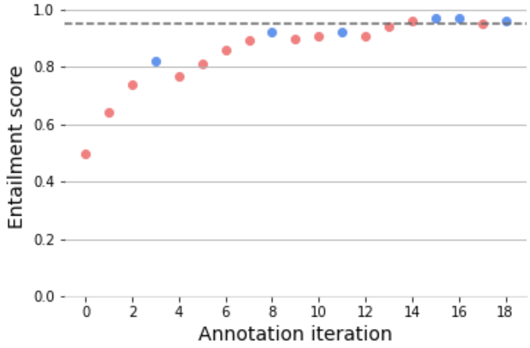

2_3: China blackmails Trump

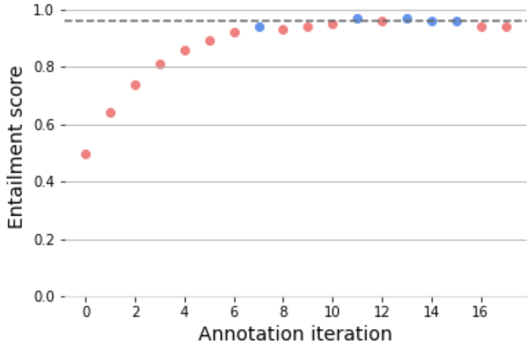

2_4: Trump works for China

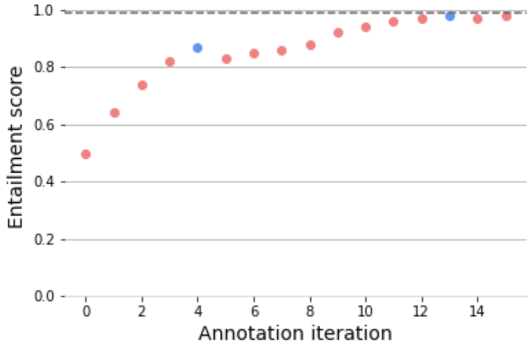

2_5: The CCP controls Trump

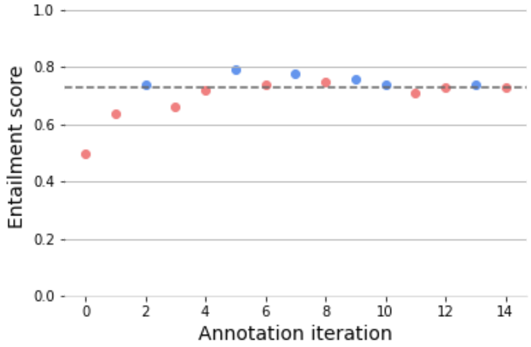

2_6: Trump’s policies hurt the United States

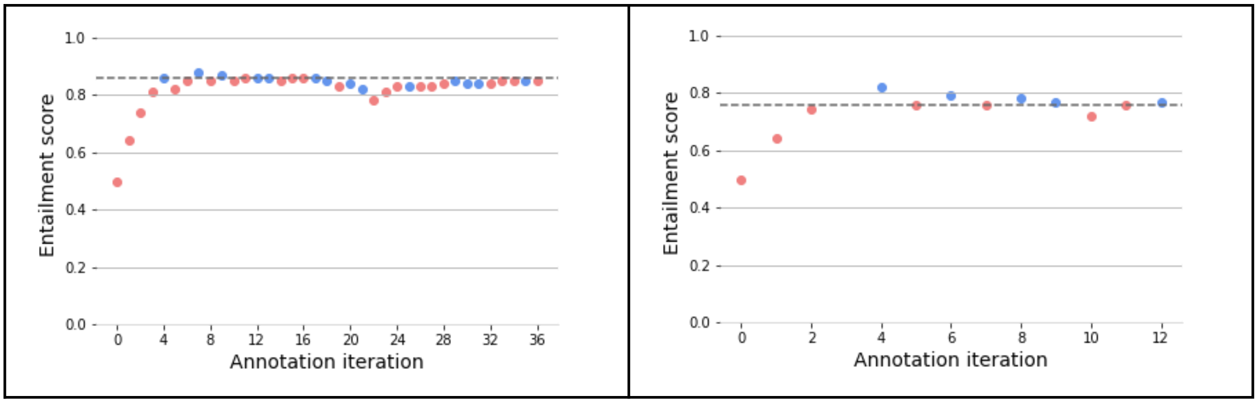

Moreover, the following table shows the values for each of the three performance heuristics for Annotator 2.

Claim	Number of true positives	Weighted accuracy	Proportion of data in 95 CI	Overall
1_1: Biden is sold out to China	6	0.91	0.174	Pay attention to confidence intervals on visualisations
1_2: Biden does business in China	0	0.99	0.130	Screen out ❌
1_3: China blackmails Biden	3	0.79	0.056	Might benefit from further testing
1_4: Biden works for China	19	0.96	0.260	Pay attention to confidence intervals on visualisations
1_5: The CCP controls Biden	13	0.80	0.107	Might benefit from further testing AND Pay attention to confidence intervals on visualisations
1_6: Biden's policies hurt the United States	11	1.00	0.482	Pay attention to confidence intervals on visualisations
2_1: Trump is sold out to China	3	0.88	0.016	Keep ✅
2_2: Trump does business in China	2	0.80	0.044	Might benefit from further testing
2_3: China blackmails Trump	0	0.89	0.004	Screen out ❌

2_4: Trump works for China	6	0.83	0.006	Might benefit from further testing
2_5: The CCP controls Trump	2	0.94	0.001	Keep ✅
2_6: Trump's policies hurt the United States	5	0.71	0.646	Keep ✅

Annotator 3

The figures below show the annotation trace returned by the threshold-tuning for Annotator 3.

1_1: Joe Biden is sold out to China

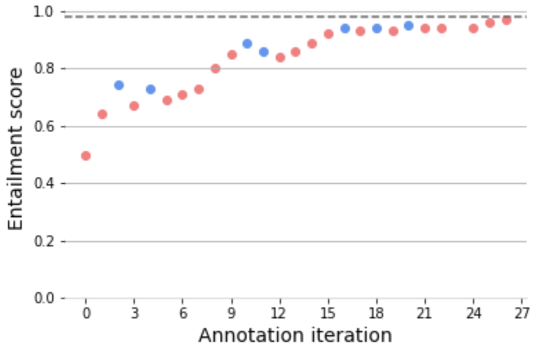

1_2: Biden does business in China

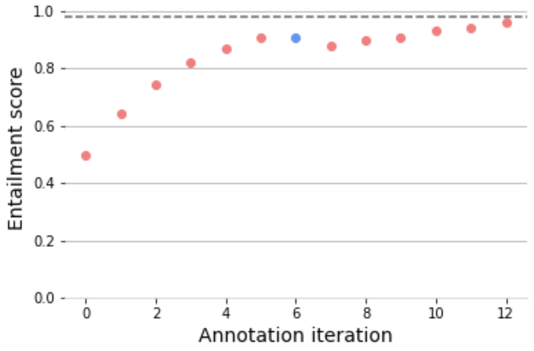

1_3: China blackmails Biden

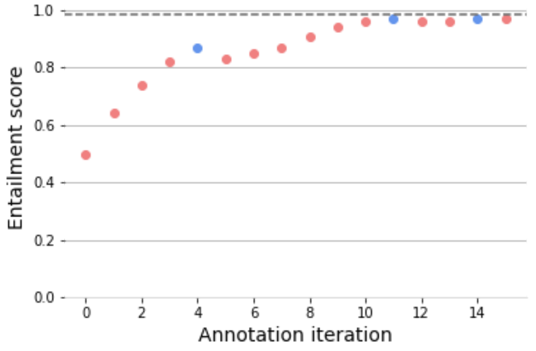

1_4: Biden works for China

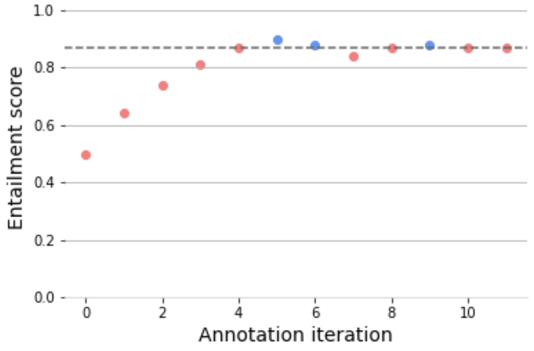

1_5: The CCP controls Biden

1_6: Biden's policies hurt the United States

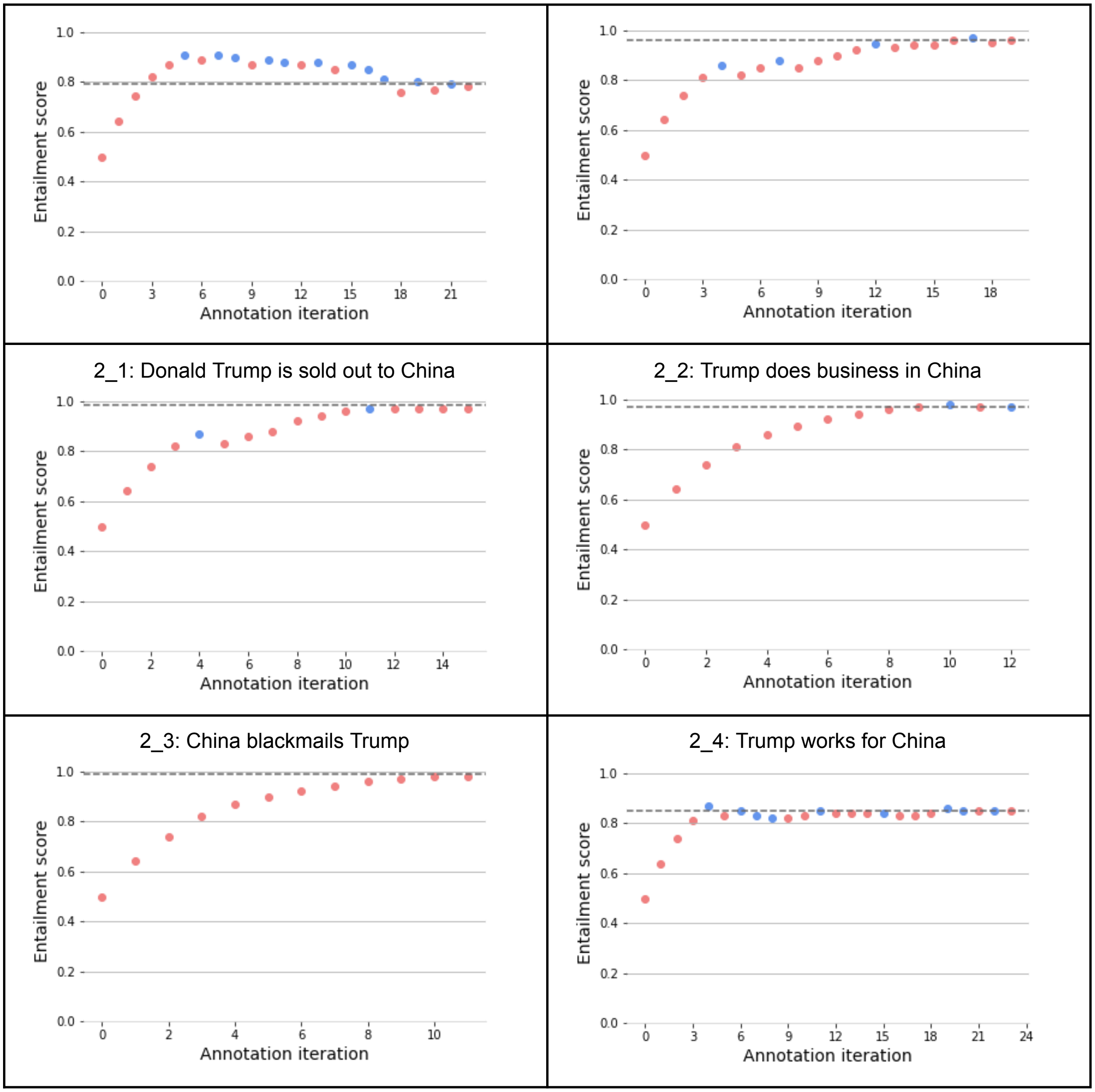
2_1: Donald Trump is sold out to China
2_2: Trump does business in China
2_3: China blackmails Trump
2_4: Trump works for China
Entailment score
Annotation iteration

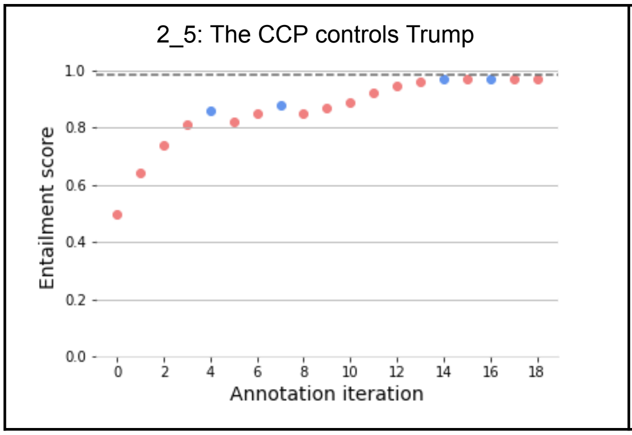

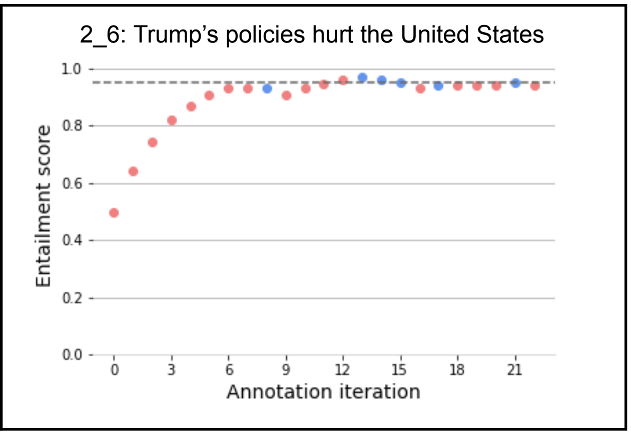

Moreover, the following table shows the values for each of the three performance heuristics for Annotator 3.

Claim	Number of true positives	Weighted accuracy	Proportion of data in 95 CI	Overall
1_1: Biden is sold out to China	0	0.93	0.117	Screen out ❌
1_2: Biden does business in China	0	1.000	0.124	Screen out ❌
1_3: China blackmails Biden	0	0.78	0.040	Screen out ❌
1_4: Biden works for China	3	0.94	0.063	Keep ✅
1_5: The CCP controls Biden	10	0.90	0.089	Keep ✅
1_6: Biden's policies hurt the United States	1	0.97	0.114	Keep ✅
2_1: Trump is sold out to China	0	0.89	0.008	Screen out ❌
2_2: Trump does business in China	2	1.000	0.046	Keep ✅
2_3: China blackmails Trump	0	0.94	0.004	Screen out ❌
2_4: Trump works for China	5	0.96	0.001	Keep ✅
2_5: The CCP controls Trump	0	1.000	0.001	Screen out ❌
2_6: Trump's policies hurt the United States	3	0.76	0.028	Might benefit from further testing AND Pay attention to confidence intervals on visualisations

I. Evaluation: extra material

1. Taxonomy of claims for topic classification task

Sentiment classification task

Given that the positive and negative labels are mutually exclusive for this sentiment task, we apply a softmax function to the entailment scores for both claims so their sum is equal to 1 before the threshold-tuning step. Moreover, we use the threshold-tuning step only for the negative claim: after all, given the binary nature of this classification task, any extract not classified as "negative" can automatically be classified as "positive".

Claim	Sentiment
The economy is performing well overall	Positive
The economy is performing badly overall	Negative

Topic classification task

- Mapping of topic to topic number

Once the thresholds for each claim in this taxonomy is obtained, we use these thresholds to normalise the NLI scores such that, for each claim, scores below the threshold are mapped to the [0, 0.5] range and while the rest are mapped to the [0.5, 1] range. Formally, this is achieved with the following piecewise linear transformation:

$$f(x, t_c) = \begin{cases} \frac{0.5x}{t_c}, & \text{for } s \leq t_c \\ 0.5 + \frac{0.5(x - t_c)}{1 - t_c}, & \text{for } s > s_t \end{cases}$$

…where *x* is the NLI score for an excerpt/claim input pair and t_c is the threshold for that claim. The topic prediction for a given excerpt is then decided by the claim with the highest normalized score. Note however that, while most of the claims for this task map to a single topic, some map to several. For instance, the claim “This quote is about sustainable economic development” maps to both the “Economy” topic and the “Welfare & Quality of Life” topic, which covers issues related to environmental protection. If the claim with the highest normalized score maps to several topics, the next claims are considered in order to decide the topic. For instance, if the highest-scoring claim (after normalization) maps to topics 4 and 5, the second maps to topic 6, and the third to topics 3 and 5, then the final prediction is topic 5 (because out of all the claims with 4 and 5, the second highest-scoring claim maps to 5 and not 4).

Topic number	Topic
1	External Relations
2	Freedom & Democracy
3	Political System
4	Economy
5	Welfare & Quality of Life
6	Fabric of Society
7	Social Groups

- Taxonomy of claims with mapping of each claim to its topic number

Claim	Topic
This quote is about external geopolitical relations	1
This quote is about foreign special relationships or foreign aid	1
This quote is about the military	1
This quote is about peace	1
This quote is about international co-operation	1
This quote is about national independence and sovereignty	1, 6
This quote is about political integration and unions like the EU	1

This quote is about internationalism	1
This quote is about freedom	2
This quote is about human rights	2
This quote is about democracy	2
This quote is about the country's constitution	2
This quote is about state coercion	2
This quote is about democratic safeguards	2, 3
This quote is about referendums	2
This quote is about the political system	3
This quote is about federalism or centralised government	3
This quote is about governmental and administrative efficiency	3
This quote is about political corruption	3
This quote is about the competence of politicians	3
We need a strong and stable government	3
This quote is about pre-democratic elites	3
This quote is about the civic rehabilitation and compensation of politically persecuted people	3, 2
This quote is about local government	3
This political party is incompetent	3
We are a competent political party	3
This quote is about the economy	4
This quote is about market regulations	4
This quote is about governmental economic planning	4
This quote is about free trade	4
This quote is about public demand and social expenditure	5
This quote is about economic growth	4, 5

This quote is about technology and infrastructure	4
This quote is about economic nationalisation	4
This quote is about sustainable economic development	4, 5
This quote is about protecting the environment or animals	5
This quote is about culture, arts and sports	5
This quote is about equality and social justice	5
This quote is about the welfare provision	5
This quote is about the education	5, 6, 4
This quote is about social benefits	5
This claim is about climate change	5
This claim is about protecting the environment	5
This quote is about immigration	6, 7
This quote is about traditional or religious moral values	6
This quote is about the family, divorce, abortion, adoption or marriage	6
This quote is about law enforcement and crime	6
This quote is about solidatiry or volunteering	6
This quote is about activism	6
This quote is about mulitculturalism and cultural diversity	6
This quote is about immigrant integration	6
This quote is about indigenous rights	6
This quote is about safety	6
This quote is about our common identity	6
This quote is about trade unions, the working class or the unemployed	7
This quote is about agriculture or farmers	7
This quote is about the middle class and professional groups	7, 5, 4

This quote is about special interest groups	7
This quote is about jobs	7, 4
This quote is about disabled people	7, 5
This quote is about people from the LGBTQ+ community	7, 2, 6
This quote is about women	7, 5, 6
This claim is about unemployment or wages	7, 4
This claim is about workers rights	7
This claim is about the elderly	7, 5
This claim is about young people	7, 5

2. Accuracy, Precision, Recall and F1-score

For a given label (i.e. either “contains the claim” or “does not contain the claim”), the precision is the proportion of all data predicted to have the label that should indeed have this label. The recall is the proportion of all data that should have the label that was indeed predicted to have it. The f1-score is then the harmonic mean of the precision and recall. The accuracy simply corresponds to the proportion of all predictions that are correct.

The equations for accuracy, precision, recall and f1-score are the following, where “TP” is the number of true positives, “TN” the number of true negatives, “FP” the number of false positives and “FN” the number of false negatives:

$$Accuracy = \frac{TP + TN}{TP + TN + FP + FN}$$

$$Precision = \frac{TP}{TP + FP}$$

$$Recall = \frac{TP}{TP + FN}$$

$$f_1 = \frac{2 \times Precision \times Recall}{Precision + Recall}$$

Using macro-averaging to calculate the precision, recall and f1-score means that, for each task, all labels (e.g. “contains the claim”/“does not contain the claim”, “negative”/“positive”) are given the same importance irrespective of their prevalence in the test set (or in the entire dataset).

3. Performance per claim per annotator

- Ground truth as provided by Annotator 1

Claim	Accuracy	Precision	Recall	F1-score
1_4	0.65	0.66	0.60	0.58
1_5	0.65	0.65	0.65	0.65
1_6	0.77	0.61	0.60	0.60
2_1	0.84	0.56	0.52	0.51
2_2	0.92	0.80	0.61	0.65
2_4	0.72	0.65	0.65	0.65
2_5	0.75	0.64	0.55	0.55
**Avg**	**0.76**	**0.65**	**0.60**	**0.60**

- Ground truth as provided by Annotator 2

Claim	Accuracy	Precision	Recall	F1-score
1_1	0.63	0.73	0.62	0.57
1_3	0.85	0.69	0.58	0.60
1_4	0.70	0.66	0.56	0.55
1_5	0.75	0.75	0.75	0.75
1_6	0.77	0.76	0.74	0.75
2_1	0.86	0.64	0.56	0.57

2_2	0.91	0.95	0.68	0.74
2_4	0.54	0.58	0.56	0.52
2_5	0.76	0.74	0.64	0.65
2_6	0.82	0.77	0.75	0.76
**Avg**	**0.76**	**0.73**	**0.65**	**0.65**

- Ground truth as provided by Annotator 3

Claim	Accuracy	Precision	Recall	F1-score
1_4	0.72	0.64	0.58	0.58
1_5	0.80	0.68	0.70	0.69
1_6	0.92	0.72	0.61	0.65
2_2	0.97	0.99	0.75	0.83
2_4	0.86	0.75	0.73	0.74
2_6	0.97	0.89	0.83	0.86
**Avg**	**0.87**	**0.78**	**0.70**	**0.72**

4. Fine-tuning BERT

For each task, we fine-tuned the “bert-base-uncased” model available via the HuggingFace platform with an AdamW optimizer. This model was fine-tuned to perform a binary classification task. For the US elections task/dataset, the input into the model was formatted as the tweet to be classified followed by the “[SEP]” token (used in the context of fine-tuning BERT models to separate inputs into the model when several inputs are used), and in turn followed by the claim to be detected in the tweet. An exemplar input would therefore look something like this:

> *“A vote for Joe Biden is a vote for China [SEP] Joe Biden works for China”*

The BERT model would then return two scores, together adding to 1: if the first score is greater than the second, the label is “No”, whereas if it is lower, the label is “Yes”. Conceptually, the label would therefore correspond to whether or not the tweet contains, or is making, the claim.

The classification task could have been formulated differently. For instance, one alternative would have been to treat each claim as its own class, such that the model would either return a probability distribution over all these classes (in which case, claims are mutually exclusive) or a binary classification score for each class (in which case, several claims can occur together). We believe that these alternatives would however not perform as well as the one we chose. After all, our set up requires the model to learn a single function between input and output (i.e. the general task of recognising when a tweet contains a claim, independently of the specific claim itself) and provides several hundreds of examples for the model to learn from (312 for Annotator 1, 284 for Annotator 2 and 236 for Annotator 3). Had the claims been treated as separate classes, however, the model would not be required to learn this single function, but rather would need to learn a different function for each specific claim with only around 20 examples to learn from each time.

One thing to note is that the model would normally be "validated" throughout training on an unseen validation set to identify at which epoch (i.e. iteration over the training data) the model stops learning the general pattern underlying the classification task and start simply memorising the labels in the training set (i.e. "overfitting" the training data). The validation set is usually a sample of around 10% of the training set isolated before training is launched. Here, however, due to the already very small size of the training set, we chose not to use a validation set and simply trained for 3 epochs and selected the version of the model from these three epochs that worked best on the unseen examples from the test set. This corresponded to the 3rd epoch for Annotator 1 and 2, and the very first epoch for Annotator 1. The implication of not using a validation set is that the f1-scores obtained on the test set may be overestimates of the real performance, had the number of epochs of training been selected based on an independent sample of unseen data set.

When fine-tuning BERT for the sentiment and topic classification tasks, however, we use a slightly different fine-tuning procedure. Given the availability of extra data, we create a separate validation set of about 10% the size of the training set. We then fine-tune the BERT model for 20 epochs and use the model with the best performance (in terms of loss) on the validation set.

5. Fine-tuning $BART_{MNLI}$

Fine-tuning $BART_{MNLI}$ according to Laurer et al. (2024) requires formulating "hypotheses" (i.e. the equivalent of our "claims") and pre-processing the data such that each text is concatenated with each claim. For instance, in the case of our sentiment classification task, the text "Retail Sales Give Investors Reason to Pause" would generate the following two new texts:

```
"Retail Sales Give Investors Reason to Pause. [SEP] The economy is
performing well overall."
```

```
“Retail Sales Give Investors Reason to Pause. [SEP] The economy is
performing badly overall.”
```

…where “[SEP]” stands for the separator token of the NLI model used. The first of these two newly generated texts would then be annotated with the “contradiction” or “neutral” class while the second would be annotated with the “entailment” class.

In terms of the fine-tuning itself, we follow the recommendations from Laurer et al. (2024) and use a warm up ratio of 0.4 over 20 epochs. The final model used for inference on the test set is the one that performs the best (in terms of loss) on the validation set (10% of the training set). Due to computational constraints, we do not perform grid search to find the optimal warm up ratio or number of epochs.

6. Generative models: prompts and instructions

The BLOOM and Llama 2 models were prompted using the following prompt:

```
Decide whether the claim text implies the claim, answering with YES or
NO. For example:

# Text: {POSITIVE EXAMPLE}
# Claim: {CLAIM}
# Answer: Yes

# Text: {NEGATIVE EXAMPLE}
# Claim: {CLAIM}
# Answer: No

# Text: {TWEET}
# Claim: {CLAIM}
# Answer:
```

…where the {TWEET} placeholder corresponds to the tweet being labeled, {CLAIM} corresponds to the claim it is being labeled with, and {POSITIVE EXAMPLE} and {NEGATIVE EXAMPLE} are pre-defined positive and negative examples for that claim. These examples are shown in the following Tables:

- US elections task

Claim	Positive example	Negative example
1_1: Biden is sold out to China	Trump should only refer to Biden during the debate as "the Big guy" as Biden is referred to in the emails showing the international	@JoeBiden Joe Biden doesn’t deserve to live in America, much less be our president. Move to China!

	money pay offs from China and Ukraine going to Hunter "to hold for the big guy" aka Joe Biden. That would be so great!	
1_2: Biden does business in China	Joe Biden should not be faulted for Hunter's poor decisions BUT Hunter invested in Megvii, a firm providing CCTV cameras for China's Skynet program - a state wide surveillance project. There are reports that both Bidens were to make a profit off this investment.	Devastating Information On Hunter And Joe Biden Come From Top Chinese Official Defected to the US [URL]
1_3: China blackmails Biden	[HANDLE] [HANDLE] multiple videos on Hunter Biden's laptop showing him raping and torturing underage Chinese girls, and that the Chinese Communist Party is using these videos as blackmail against Democrat presidential contender Joe Biden. [URL]	[HANDLE] joe biden works for china not our country
1_4: Biden works for China	[HANDLE] So Joe Biden is now proven to be an unregistered foreign agent of China.	A vote for Joe Biden is a vote for China
1_5: The CCP controls Biden	[HANDLE] [HANDLE] Meet Joe Biden: China's Puppet #Trump2020 [HANDLE] . [URL]	Joe Biden must have paid off China
1_6: Biden's policies hurt the United States	"<HANDLE> Joe Biden China conspiracy, Joe Biden Russia conspiracy,and Joe Biden other country's conspiracy. Joe Biden got payed by each of them. Joe Biden sold out the United States to each of them . Being a traitor to the United States. Joe Biden did treason to the United States."	"Joe Biden upset that Trump closed the border with China due to coronavirus <URL> via <HANDLE>"
2_1: Trump is sold out to China	📢Trump had a secret office in China during his 1st run for president, & is partners with a government-controlled company🤔 📢Trump maintains bank accounts in China—that don't appear on his financial disclosures—because they're held under corporate names [URL]	@realDonaldTrump Donald Trump continues to call covid19 as China virus, because he wants us to believe US has so many cases is totally due to China who detected the virus but not his incompetence. He takes no responsibility for it.
2_2: Trump does business in China	[HANDLE] Oh sounds like donald trumps merchandise made in China and Bangladesh! David Letterman Exposes Donald Trump [URL] via [HANDLE]	[HANDLE] [HANDLE] [HANDLE] Selling US state dept. influence to the Chinese is bad, is corruption at the highest levels of government, and if Joe Biden did it, he needs to burn. If Kushner did anything corrupt, he and everyone involved, including Trump himself, needs to burn. Burn. Them. All.

2_3: China blackmails Trump	Donald Trump personally owes 10s of millions to the Bank of China - so China ‘and’ Russia have the stable genius by the nads. [URL]	Forbes: China Could Force Donald Trump And The Fed To Destroy The U.S. Banking System. [URL] via [HANDLE]
2_4: Trump works for China	[HANDLE] DONALD TRUMP DOES BUSINESS IN CHINA, AND WITH CHINA.	[HANDLE] Donald Trump has been pandering to China
2_5: The CCP controls Trump	[HANDLE] No. Russia, North Korea, Iran, and China kept Donald Trump in their back pocket.	CHINA IN CHARGE: The Chinese Communists are Gloating: "Good Riddance Donald Trump" [URL] via [HANDLE]
2_6: Trump’s policies hurt the United States	"Donald Trump is not working for Russia, he's working for China. This entire trade war is hurting American economic leadership both in the short term and in the long term as they turn away from imports and grow their domestic industry."	'CHINA IN CHARGE: The Chinese Communists are Gloating: "Good Riddance Donald Trump" [URL] via [HANDLE]'

- Sentiment about the economy task

Claim	Positive example	Negative example
1_1: The economy is performing well overall	Reserve Chief Optimistic on the Economy -- Calls Inflation Curb 'Vital'	Airlines Fearing a Vicious Circle Of Weak Finances and Low Morale: Squeezing Labor Costs

- Topic in manifestos task

Claim	Positive example	Negative example
0: The quote is about something other than the topics economy, international relations, society, freedom and democracy, political system, social groups, welfare. It is about none of these topics.	We expressed our opposition to motorway and road tolls.	There is a huge disparity between the numbers granted refugee status at the initial stage and the numbers successful at the appeal stage.

1: The quote is about international relations, or foreign policy, or military.	To revitalize important relationships with democratic countries, we will reclaim Canada’s role in NORAD, NATO, the Commonwealth, La Francophonie, and the Five Eyes.	A thriving cattle industry will provide social and economic benefits through more jobs.
2: The quote is about democracy, or freedom, or human rights, or constitutionalism.	There is a huge disparity between the numbers granted refugee status at the initial stage and the numbers successful at the appeal stage.	They indicate that by not maximizing the use of market forces through proper price signals, the Conservatives are creating new economic distortions and reducing the efficiency of our economy.
3: The quote is about governmental efficiency, or political authority, or decentralisation, or corruption.	With so many redundant, inefficient, and ineffective federal programs, it is no wonder that the American people have so little confidence in Washington to act effectively when federal action is really needed.	Everyone should have the right to social benefits in the case of illness or other involuntary temporary absence from work, unemployment, and disability.
4: The quote is about economy, or technology, or infrastructure, or free market.	They indicate that by not maximizing the use of market forces through proper price signals, the Conservatives are creating new economic distortions and reducing the efficiency of our economy.	Drugs, violent crimes and gangsterism are wreaking havoc in many communities.
5: The quote is about welfare, or education, or environment, or equality, or culture.	Everyone should have the right to social benefits in the case of illness or other involuntary temporary absence from work, unemployment, and disability.	To revitalize important relationships with democratic countries, we will reclaim Canada’s role in NORAD, NATO, the Commonwealth, La Francophonie, and the Five Eyes.
6: The quote is about law and order, or multiculturalism, or national way of life, or traditional morality.	Drugs, violent crimes and gangsterism are wreaking havoc in many communities.	With so many redundant, inefficient, and ineffective federal programs, it is no wonder that the American people have so little confidence in Washington to act effectively when federal action is really needed.
7: The quote is about agriculture, or social groups, or labor groups, or minorities.	A thriving cattle industry will provide social and economic benefits through more jobs.	We expressed our opposition to motorway and road tolls.

7. Comparing to human hand-coding

Our annotators required on average 9 hours to annotate our US elections test set of 1,200 tweets/claim pairs. Our approach using an NLI model with threshold-tuning, on the other hand, only required about 432 hours of data annotation before being deployed on the entire corpus of 1.3 million tweets.The time-saving benefits of using a computational approach such as our own instead of human hand-coding to label large corpora, are thus clear. However, we do not know that the quality of the predicted labels is comparable to human annotations. To evaluate whether this is the case in the context of our US elections test set, we calculate the average of the macro f1-score achieved by each pair of annotators, as benchmarked against the annotations of the third annotator. For instance, taking Annotator 1's annotations as the ground truth, we calculate the macro f1-score achieved by Annotators 2 and 3 and average these. This result can then be directly compared to the macro f1-score achieved by our method when compared to Annotator 1's annotations. This information is summarized in the table below.

As we can see, the labels obtained using our claims detection approach lead to a macro f1-score that is on average slightly lower than those provided by human annotators (0.66 vs 0.68). This difference, however, is rather small (2 percentage points). Moreover, it varies significantly between annotators. Human annotators outperform our system when Annotator 1's annotations are used as ground truth. However, the performance is well within the range of other human annotators when considering Annotator 2's annotations as ground truth, and it even out-performs them when considering Annotator 3's annotations as ground truth.

**Annotator**	**F1-score**
Annotator 1	0.73
Annotator 2	0.68
Annotator 3	0.63
**Average**	**0.68**
